# Inhibition of Classical and Alternative Modes of Respiration in *Candida albicans* Leads to Cell Wall Remodeling and Increased Macrophage Recognition

**DOI:** 10.1128/mBio.02535-18

**Published:** 2019-01-29

**Authors:** Lucian Duvenage, Louise A. Walker, Aleksandra Bojarczuk, Simon A. Johnston, Donna M. MacCallum, Carol A. Munro, Campbell W. Gourlay

**Affiliations:** a Kent Fungal Group, School of Biosciences, University of Kent, Kent, United Kingdom; b MRC Centre for Medical Mycology at the University of Aberdeen, Institute of Medical Sciences, University of Aberdeen, Aberdeen, United Kingdom; c Department of Infection, Immunity & Cardiovascular Disease and Bateson Centre, University of Sheffield, Sheffield, United Kingdom; Monash University; Tel Aviv University

**Keywords:** *Candida albicans*, cell wall, mitochondria, mycology, yeast

## Abstract

The human fungal pathogen Candida albicans requires respiratory function for normal growth, morphogenesis, and virulence. Mitochondria therefore represent an enticing target for the development of new antifungal strategies. This possibility is bolstered by the presence of characteristics specific to fungi. However, respiration in C. albicans, as in many fungal organisms, is facilitated by redundant electron transport mechanisms, making direct inhibition a challenge. In addition, many chemicals known to target the electron transport chain are highly toxic. Here we made use of chemicals with low toxicity to efficiently inhibit respiration in C. albicans. We found that use of the nitric oxide donor sodium nitroprusside (SNP) and of the alternative oxidase inhibitor salicylhydroxamic acid (SHAM) prevents respiration and leads to a loss of viability and to cell wall rearrangements that increase the rate of uptake by macrophages *in vitro* and *in vivo*. We propose that treatment with SNP plus SHAM (SNP+SHAM) leads to transcriptional changes that drive cell wall rearrangement but which also prime cells to activate the transition to hyphal growth. In line with this, we found that pretreatment of C. albicans with SNP+SHAM led to an increase in virulence. Our data reveal strong links between respiration, cell wall remodeling, and activation of virulence factors. Our findings demonstrate that respiration in C. albicans can be efficiently inhibited with chemicals that are not damaging to the mammalian host but that we need to develop a deeper understanding of the roles of mitochondria in cellular signaling if they are to be developed successfully as a target for new antifungals.

## Abstract (precis)

Current approaches to tackling fungal infections are limited, and new targets must be identified to protect against the emergence of resistant strains. We investigated the potential of targeting mitochondria, which are organelles required for energy production, growth, and virulence, in the human fungal pathogen Candida albicans. Our findings suggest that mitochondria can be targeted using drugs that can be tolerated by humans and that this treatment enhances their recognition by immune cells. However, release of C. albicans cells from respiratory inhibition appears to activate a stress response that increases the levels of traits associated with virulence. Our results make it clear that mitochondria represent a valid target for the development of antifungal strategies but that we must determine the mechanisms by which they regulate stress signaling and virulence ahead of successful therapeutic advance.

## INTRODUCTION

Candida albicans is one of the most prevalent fungal pathogens and a major cause of nosocomial infections which have a high mortality rate (1). Current antifungals, although effective, target a limited number of cellular processes, and the development of new therapeutic approaches is essential. C. albicans requires mitochondrial function for normal growth, morphogenesis, and virulence (2–4), but mitochondria had not been exploited as a therapeutic target to date. Given the central role of this organelle in processes essential for growth, maintenance, and adaptability, coupled to the presence of fungal specific characteristics, it may be possible to develop therapies based on mitochondrial inhibition.

**FIG 1:**
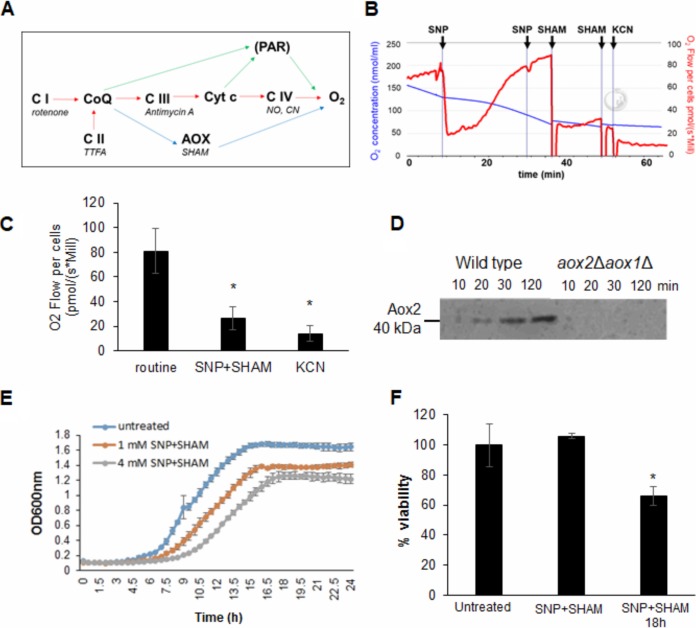
SNP+SHAM treatment inhibits respiration and reduces viability. (A) Schematic of electron transport pathways in C. albicans, representing complexes I to IV of the classical pathway (red arrows), the alternative oxidase pathway (AOX) (blue arrows), and the parallel pathway (PAR) (not shown in detail) (green arrows). Shown in italics are commonly used inhibitors. (B) A representative example of respiration in whole C. albicans cells determined using high-resolution respirometry. SNP and SHAM were added where indicated, resulting in final concentrations of 1 and 2 mM for both. Potassium cyanide (KCN) was added to a final concentration of 2 mM. (C) Respiration was inhibited by SNP+SHAM or 2 mM KCN treatment, and the results were compared to those seen with untreated controls (*n* = 6). *, *P* < 0.01. (D) Aox2 expression was monitored by immunoblotting following addition of 1 mM SNP in samples taken from *aox2*Δ *aox1*Δ or control cells. (E) Growth was monitored over time in the presence of increasing concentrations of SNP+SHAM. (F) Cells were grown in the presence of 1 mM SNP–0.5 mM SHAM for 3 h and 18 h before viability was determined by CFU assay (*n* = 4). Graphs show means ± standard deviations. Student's *t* test was used to compare groups. *, *P* < 0.01.

C. albicans is a Crabtree effect-negative yeast and relies mainly on oxidative phosphorylation for ATP production during growth and morphogenesis. It possesses a classical electron transfer chain (ETC), consisting of complexes I to IV, as well as a cyanide-insensitive alternative oxidase, which permits respiration when the classical chain is inhibited (Fig. 1A) (5). A functional electron transport system has been shown to be important for aspects of C. albicans biology that are linked to virulence. For example, inhibition of respiration in C. albicans and other pathogenic fungi leads to a decreased growth rate (6). Mutants defective in respiration have consistently been shown to affect the hyphal morphological switch, an important determinant of virulence in C. albicans, whether negatively (*goa1*Δ, *ndh51*Δ) (7) or positively (*dbp4*Δ, *hfl1*Δ, *rbf1*Δ) (8). Respiratory inhibition has been linked to reduced activation of Ras1 signaling, a major determinant of morphogenesis, via an uncharacterized mechanism that correlates with reduced ATP levels (9). A link between electron transport and control of Ras1 has also been suggested to regulate a loss of hyphal growth under anaerobic conditions (10).

The cell wall is critical for viability of fungal pathogens and is a major determinant of virulence. Its composition and organization also determine the recognition of C. albicans by the immune system (11–13). Recent work has shown that masking of cell wall components facilitates immune evasion. Changes in surface beta-glucan exposure can occur in response to a variety of stimuli, including changes in carbon sources and pH (14, 15). A number of studies have suggested that mitochondrial function may be linked to the maintenance of the C. albicans cell wall. Loss of the complex I regulator Goa1 revealed a link between respiration and sensitivity to cell wall-damaging agents (16) and cell wall architecture (17). In addition, impairment of mitochondrial function by deletion of *SAM37*, which encodes a component of the mitochondrial outer membrane sorting and assembly machinery (SAM) complex, also resulted in defects in cell wall integrity (CWI) (18). Mitochondrial interaction with the endoplasmic reticulum is also important for maintaining cell wall integrity; deletion of the mitochondrial GTPase Gem1 led to abnormal mitochondrial morphology and susceptibility to cell wall stress through reduced Cek1 activation (19). A better understanding of the links between mitochondrial function and virulence and, indeed, of the consequences of respiration inhibition are crucial if we are to develop mitochondria as a potential drug target.

In an attempt to understand the consequences of respiration inhibition in C. albicans, we adopted a pharmacological approach. We made use of nitric oxide (NO), which inhibits respiration at the level of complex IV and has a safe record for use as a vasodilator (20) and in dermatologic applications (21). NO has been evaluated as a therapy in a variety of bacterial and fungal infections, including infections by Pseudomonas aeruginosa in cases of cystic fibrosis and infections caused by dermatophytes (22–24). NO inhibition of cytochrome *c* oxidase at low concentrations is rapidly reversible by oxygen treatment. However, permanent inhibition of respiration can result at higher NO concentrations (25). In addition, NO causes the formation of reactive nitrogen species (such as peroxynitrite) which can damage mitochondrial function and which have been shown to have strong antifungal activity (26). Several studies reported the efficacy of NO against C. albicans (27–29). The alternative oxidase can be inhibited by hydroxamic acids such as salicylhydroxamic acid (SHAM). The low *in vivo* toxicity of alternative oxidase inhibitors such as SHAM and ascofuranone has been evaluated with respect to their ability to treat trypanosomiasis (30, 31).

We found that C. albicans cells are highly adaptive to classical respiration inhibition but that a combination of SHAM and the NO donor sodium nitroprusside (SNP) (SNP+SHAM) led to fitness defects and loss of viability. In addition, treatment with SNP+SHAM led to cell wall organization defects that unmasked C. albicans cells and to increased immune cell recognition in cell culture and animal models. However, release of cells from SNP+SHAM treatment led to a rapid activation of the hyphal transition program and increased virulence in a mouse model. Our data suggest that mitochondria form part of a complex response network that is imbedded within the cellular responses required for C. albicans virulence.

## RESULTS

### SNP+SHAM inhibits respiration and growth and reduces viability in C. albicans.

Our goal was to identify conditions that would allow the reproducible inhibition of classical and alternative respiration in C. albicans. Complex I can be inhibited by rotenone, complex III by antimycin A, and complex IV by cyanide (Fig. 1A). However, although effective as research tools, these compounds are highly toxic and unsuitable for use in humans. As an alternative to treatment with cyanide compounds, oxygen consumption was measured upon exposure of cells to the nitric oxide donor SNP, which inhibits cytochrome *c* oxidase, and to the alternative oxidase (Aox) inhibitor SHAM. Initial titration experiments suggested that the concentrations of 1 mM SNP and 0.5 mM SHAM were suitable for our purpose. Recovery from NO-induced inhibition of classical respiration by SNP addition was rapid, with full restoration achieved within 30 min (Fig. 1B). Further application of SNP did not lead to a reduction in oxygen consumption, suggesting that cells had switched to alternative respiration upon SNP treatment (Fig. 1B). This was confirmed by subsequent addition of SHAM, which decreased the respiration level significantly (Fig. 1B). Addition of 2 mM cyanide was sufficient to inhibit the remaining respiration. Simultaneous addition of SNP+SHAM led to a significant loss of respiration that was comparable to that observed upon cyanide addition (Fig. 1C; see also Fig. S1A in the supplemental material). In support of the idea of a rapid transition to alternative respiration, an increase in Aox2 protein levels was detectable within 20 min following exposure to SNP (Fig. 1D). Addition of SHAM alone did not lead to a decrease in respiration (Fig. S1B), suggesting that AOX did not contribute significantly except under conditions of induction in response to inhibition of the classical respiratory chain.

10.1128/mBio.02535-18.2 FIG S1 SNP+SHAM treatment induces surface exposure of β(1,3)-glucan in both the wild-type and *upc2*Δ strains. (A) Respiration was assessed in C. albicans cells prior to and following addition of 4 mM SNP+SHAM, and the resultant respiration levels are given (*n* = 3). (B) Respiration in whole C. albicans cells was determined using high-resolution respirometry. SHAM was added at 0.5, 1, and 2 mM concentrations in sequential doses, and the resultant respiration data are given. (C) Wild-type and *upc2*Δ mutant cells were treated with 1 mM SNP–0.5 mM SHAM for 18 h and stained with dectin-1 as described in Materials and Methods. Dectin-1 staining of the cell wall was assessed manually from microscopy images (*n* = 3). Three independent experiments were analyzed. Graphs show means ± standard deviations. Student’s *t* test was used to compare groups. *, *P* < 0.01. Download FIG S1, PDF file, 0.3 MB. Copyright © 2019 Duvenage et al. 2019 Duvenage et al. This content is distributed under the terms of the Creative Commons Attribution 4.0 International license.

Given the effects of SNP+SHAM on respiration, we investigated its effects on growth and viability. Incubation of C. albicans cells with SNP+SHAM led to a decrease in growth and in final biomass in a dose-dependent manner (Fig. 1E). Treatment with SNP+SHAM for 3 h did not have a significant effect on viability; however, prolonged SNP+SHAM exposure resulted in decreased viability compared to the results seen with untreated controls (Fig. 1F). These data suggest that C. albicans possesses a robust and adaptable respiratory system that can be targeted using a SNP+SHAM cotreatment approach.

### SNP+SHAM treatment induces structural rearrangements within the cell wall.

**FIG 2:**
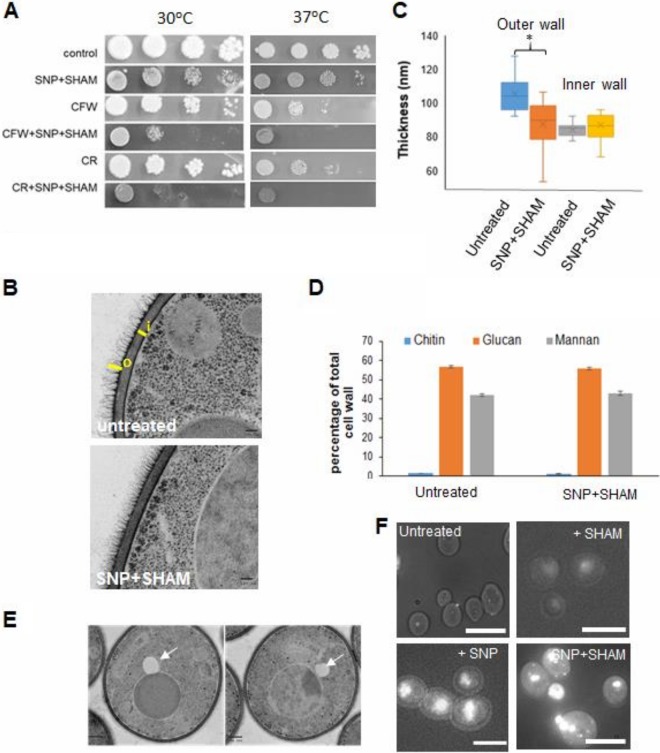
SNP+SHAM treatment leads to cell wall alterations and altered lipid metabolism. (A) Wild-type cells were serially diluted (1:10 dilutions), and equal volumes were spotted onto YPD plates (at 30 or 37°C) containing 1 mM SNP–0.5 mM SHAM and 25 µg/ml calcofluor white (CFW) or 40 µg/ml Congo red (CR). (B) Cells were treated with 1 mM SNP–0.5 mM SHAM for 18 h and processed for transmission electron microscopy (TEM). Representative examples of untreated and SNP+SHAM-treated cells are shown. Bar = 100 nm. (C) Quantification of outer cell wall (o) and inner cell wall (i) thicknesses was carried out from TEM images. Measurements were taken at 5 points along the cell wall for 25 cells per group. Bar = 100 nm. (D) Cell wall material was extracted and acid hydrolyzed from cells treated with 1 mM SNP–0.5 mM SHAM for 18 h, followed by HPLC analysis (*n* = 3). Graphs show means ± standard deviations. (E) TEM images from cells treated with SNP+SHAM. Arrows indicate lipid droplets. Bar = 500 nm. (F) Control and SNP-, SHAM-, or SNP+SHAM-treated cells were stained using LD540 neutral lipid stain and viewed by fluorescence microscopy. Bar = 10 µm. Student's *t* test was used to compare groups. *, *P* < 0.01.

Previous studies suggested a link between respiration and cell wall integrity (8, 16). Our goal was to determine whether chemical inhibition of respiration using SNP+SHAM could negatively impact cell wall integrity. In line with this, SNP+SHAM exposure led to a reduction in growth and to sensitivity to the cell wall-damaging agents calcofluor white (CFW) and Congo red (CR) at both 30 and 37°C (Fig. 2A). We employed transmission electron microscopy to examine cell wall structure following SNP+SHAM treatment (Fig. 2B). This analysis revealed that the outer cell wall, composed of mannans and cell wall proteins, but not the inner cell wall was reduced in thickness compared to untreated cells (Fig. 2B and C). High-performance liquid chromatography (HPLC) analysis of acid-hydrolyzed cell wall components showed that SNP+SHAM treatment caused no significant changes in the relative levels of chitin, glucan, or mannan (Fig. 2D). This suggests that cell wall changes induced by SNP+SHAM may be due to changes in organization rather than to gross changes in the relative levels of cell wall components. Interestingly, we also observed the presence of a large structure adjacent to the vacuole within SNP+SHAM-treated cells (Fig. 2E). We confirmed this to be a lipid droplet by the use of the neutral lipid stain LD540 (Fig. 2F). SNP treatment alone was sufficient to induce lipid droplet accumulation, suggesting a link to inhibition of classical respiration (Fig. 2F). This finding suggests a link between respiratory inhibition and the regulation of lipid metabolism in C. albicans.

### Caspofungin resistance reveals a link between respiration inhibition and Upc2.

**FIG 3:**
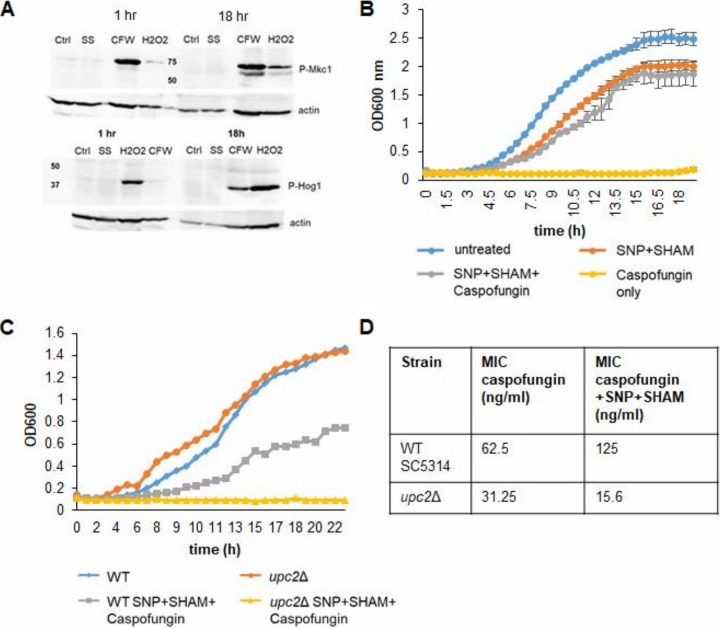
SNP+SHAM-induced cell wall changes do not induce Mkc1 or Hog1 activation but alter caspofungin sensitivity, which requires Upc2. (A) Cells were treated with 1 mM SNP–0.5 mM SHAM (SS) for 1 h or 18 h. Activation of Mkc1 was determined using p44/42 antibody. Cells treated with 25 µg/ml calcofluor white (CFW) were used as a positive control. Activation of Hog1 was determined using p38 antibody, with cells treated with 2 mM hydrogen peroxide used as a positive control. The level of anti-actin on the respective blots is shown as a loading control. (B) Wild-type cells were grown with 1 mM SNP–0.5 mM SHAM and/or 100 ng/ml caspofungin (*n* = 3). (C) The *upc2*Δ mutant and the corresponding wild-type strain were grown in synthetic complete medium with 1 mM SNP–0.5 SHAM or in combination with 100 ng/ml caspofungin. A representative data set is displayed (*n* = 3). (D) Microdilution assays were performed with 3.9 ng/ml to 8 µg/ml caspofungin with or without 1 mM SNP–0.5 mM SHAM. The table shows the MICs for caspofungin for the wild-type strain and the *upc2*Δ mutant with or without SNP+SHAM (*n* = 3).

Cell wall damage can be sensed and repaired by activation of the cell wall integrity (CWI) pathway (32). To determine whether this mechanism was engaged by SNP+SHAM treatment, CWI pathway activity was measured by detection of the phosphorylated forms of Mkc1, a well-characterized marker of pathway activation (33). We also determined whether SNP+SHAM treatment led to the activation of Hog1, a major oxidative stress response regulator that also influences cell wall biosynthesis (34). Unlike the results seen with the positive controls, SNP+SHAM treatment did not cause activation of Hog1 or Mkc1 (Fig. 3A). This suggests either that the major cell wall integrity responses are not involved in SNP+SHAM-induced cell wall changes or that the mitochondrial compartment functions downstream of Hog1 and Mkc1 within the cell wall response.

To identify potential regulators of the SNP+SHAM response that may be important in cell wall organization, we treated C. albicans with a combination of SNP+SHAM and caspofungin, which targets the cell wall (Fig. 3B). We observed that SNP+SHAM treatment led to caspofungin resistance, presumably as a result of the alteration in cell wall structure (Fig. 3B). Using this phenotype, we screened a library of transcription factor deletion mutants (35) by growing the strains in the presence of SNP+SHAM in combination with caspofungin at a level that inhibited growth of the wild-type (WT) strain. A single transcription factor, Upc2, was found to be required for caspofungin resistance upon SNP+SHAM treatment. Deletion of *UPC2*, which plays an important role in the regulation of ergosterol biosynthesis (36), led to a reproducible reduction in caspofungin resistance upon exposure to cotreatment with SNP+SHAM (Fig. 3C). To confirm the results from the initial screen, microdilution growth assays of caspofungin in combination with SNP+SHAM were performed. An independently derived *upc2*Δ mutant (37) showed a caspofungin MIC that was 8-fold lower that than seen with the wild-type strain when SNP+SHAM were added in combination (Fig. 3D). These data suggests a role for Upc2 in regulating cell wall changes in response to SNP+SHAM treatment.

### Analysis of transcriptional changes in response to SNP+SHAM treatment.

**FIG 4:**
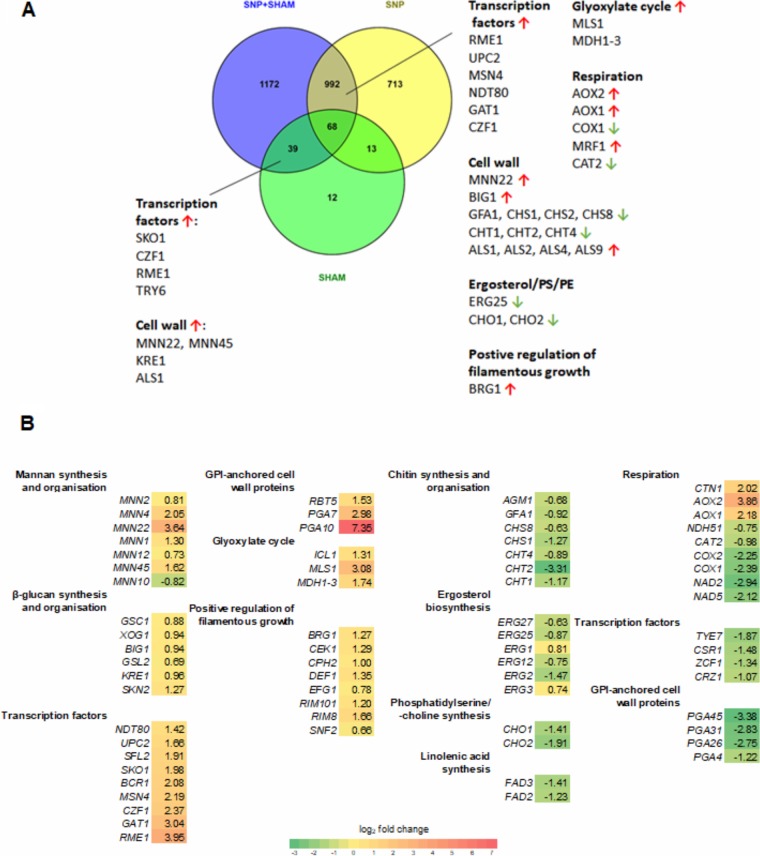
Transcriptome of wild-type C. albicans treated with SNP, SHAM, or SNP+SHAM. (A) Venn diagram showing overlap in differentially expressed genes between SNP, SHAM, and SNP+SHAM treatments by RNA-seq analysis as described in Materials and Methods. PE, phosphatidylethanolamine. (B) A selection of differentially expressed genes in SNP+SHAM-treated cells were grouped by GO term, with log_2_ fold change versus untreated shown. Green indicates downregulated genes, and red/yellow indicates upregulated genes. Differentially expressed genes were identified by Cuffdiff v2.1.1, with false-discovery-rate (*q*) values of <0.05 being considered statistically significant.

To further investigate the changes caused by SNP+SHAM, we examined the transcriptional response of C. albicans to this treatment using RNA sequencing. RNA was extracted from log-phase yeast cells exposed to 1 mM SNP or 0.5 mM SHAM or both for 30 min (Fig. 4; see also Tables S2, S3, and S4 in the supplemental material). A short exposure time was selected in order to capture early alterations rather than profiles from the expansion of adapted cell lineages. SNP and SNP+SHAM treatment led to differential expression of over 1,500 genes, whereas SHAM treatment led to a smaller range of responses, with only 131 genes differentially expressed (Fig. 4A; see also Tables S2, S3, and S4). A significant overlap of differentially expressed genes within the SNP and SNP+SHAM treatment data sets was observed (Fig. 4A). As expected, the *AOX2* and *AOX1* genes encoding the alternative oxidases and genes required for a response to nitric oxide, such as *YHB1*, were upregulated in both the SNP-only group and the SNP+SHAM group. Several genes encoding components of classical respiration were downregulated upon SNP+ SHAM treatment, whereas genes involved in the glyoxylate cycle (*MLS1* and *ICL1*) and glycolysis and gluconeogenesis (*FBP1*, *GPM1*, *GPM2*, *PCK1*, *PGI1*, *PGK1*) (Tables S2 and S4) were upregulated. These changes suggest a shift in carbon metabolism in response to respiration inhibition.

A number of transcriptional regulators were upregulated in response to both classical and alternative respiration inhibition (Fig. 4A). To examine which key regulators were involved in the response to SNP+SHAM treatment, we analyzed the genes on the list of those differentially expressed using PathoYeastract, a tool which ranks transcription factors in the order of the number of genes in the list that they regulate and for which there exists supporting expression evidence (38). This analysis showed that the Sko1 transcription factor has regulatory associations with 33.8% of the differentially expressed genes. *SKO1* itself was upregulated upon SNP+SHAM treatment. Interestingly, the upregulation of *SKO1* seems to have been a SHAM-specific effect, as it was not differentially expressed in the SNP-only data set. Upc2 binding sites were identified within the promoters of 13.4% of the genes differentially regulated upon SNP+SHAM treatment. This, along with the fact that *UPC2* expression was itself upregulated upon SNP+SHAM treatment, further suggests a role for this transcription factor.

Analysis of SNP+SHAM RNA-seq data showed that several genes involved in chitin synthesis and organization were downregulated, including the *CHT1*, *CHT2*, and *CHT4* chitinase genes (Fig. 4B). Mannan biosynthesis and organization genes were upregulated, as were genes involved in β-glucan synthesis and organization. However, as no significant changes in relative glucan, mannan, or chitin levels were detected by HPLC, these changes do not seem to be realized at the protein level. One possibility is that SNP+SHAM-induced transcriptional changes led to alterations in cell wall organization but not to substantial changes in composition. Supporting this hypothesis, we observed that several glycosylphosphatidylinositol (GPI)-anchored cell wall protein genes that play a role in cross-linking cell wall components were also differentially expressed (Fig. 4B).

Genes encoding chitinase Cht2 and GPI-anchored cell wall protein Pga26, previously shown to have roles in the unmasking of cell wall β-glucan, were downregulated (15). This appears to have been a result of inhibition of classical respiration, as SNP treatment alone led to reduced expression of *CHT2* and *PGA26*. The majority of genes in the so-called “common in fungal extracellular membranes” (CFEM) family, including *PGA7*, *PGA10*, *RBT5*, and *CSA2*, were upregulated (39). These genes encode proteins with roles in iron acquisition and adhesion. Expression of several of these genes is controlled by Bcr1, a transcription factor known to be important in the hypoxic response (40), and *BCR1* was itself upregulated. Genes involved in adhesion, including several of the *ALS* genes, as well as the *RIM101* regulator were also upregulated (Fig. 4B).

SNP+SHAM treatment also led to changes in genes involved in lipid metabolism. Upregulation of several lipase genes suggested a mobilization of lipid stores (see Table S4). This is in agreement with our observation of increased formation of lipid droplets upon SNP+SHAM treatment (Fig. 2E and F). A number of genes involved in the ergosterol biosynthesis pathway were downregulated (Fig. 4B). In addition, genes involved in the synthesis of phosphatidylserine (PS) and phosphatidylcholine (PC) (*CHO1* and *CHO2*, respectively) and linoleic acid (*FAD3* and *FAD2*) were also downregulated. Collectively, these data suggest that SNP+SHAM treatment led to changes in lipid metabolism.

### SNP+SHAM treatment increased surface chitin and β-glucan exposure.

**FIG 5:**
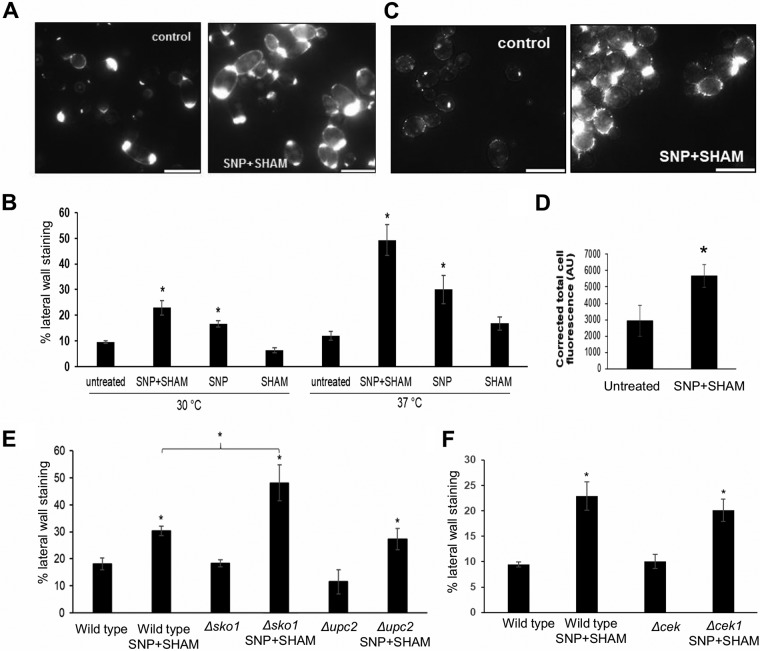
SNP+SHAM treatment induces surface exposure of chitin and β(1,3)-glucan. (A) Representative examples of untreated cells and cells treated with 1 mM SNP–0.5 mM SHAM for 18 h, stained with fluorescein isothiocyanate (FITC)-wheat germ agglutinin (WGA), and analyzed using fluorescence microscopy. (B) Cells treated with SNP, SHAM, or SNP+SHAM at 30 or 37°C were quantified for FITC-WGA staining as described in Materials and Methods (*n* = 3). (C) Representative examples of untreated and SNP+SHAM-treated cells stained with dectin-1. Bar = 10 µm. (D) Cells were quantified as described in Materials and Methods (*n* = 3). AU, arbitrary units. (E and F) Effect of SNP+SHAM treatment on chitin unmasking as determined by WGA staining in *upc2*Δ and *sko1*Δ mutants (E) and in (F) *cek1*Δ mutants. Graphs show means ± standard deviations. At least three independent experiments were performed for each analysis. Student's *t* test was used to compare groups. *, *P* < 0.001.

Our data suggests that inhibition of respiration led to cell wall rearrangement (Fig. 2B to D). To investigate this further, we assessed the exposure of chitin and β-glucan at the cell surface using fluorescent probes. Wheat germ agglutinin (WGA) binds chitin in fungal cell walls but, due to its large size, cannot penetrate the cell wall to stain chitin in the innermost layers where it is usually found (41). WGA therefore typically stains structures such as bud scars where chitin is exposed, and staining of the lateral cell wall indicates that chitin is exposed at the surface of the cell wall. SNP+SHAM-treated cells exhibited greater lateral wall staining than untreated cells (Fig. 5A and B). An increase in chitin exposure was observed upon SNP treatment alone and was enhanced at 37°C compared to 30°C (Fig. 5B). These results suggest that SNP+SHAM treatment caused exposure of the normally hidden chitin in the cell wall but that this effect was linked to the effects of NO upon the classical respiratory chain. This effect was also previously observed in a *cek1*Δ mutant (42), suggesting that SNP+SHAM-induced chitin unmasking is independent of Cek1 signaling (Fig. 5F).

Dectin-1 recognizes β(1,3)-glucan in the inner fungal cell wall and is one of the major receptors involved in the immune response to fungal pathogens (43). To determine whether SNP+SHAM treatment causes a change in β(1,3)-glucan exposure in C. albicans, a soluble form of dectin-1 was used to stain treated cells. SNP+SHAM pretreatment significantly increased the level of dectin-1 staining of the cell wall (Fig. 5C and D). This suggests that the cell wall changes induced by SNP+SHAM treatment resulted in cell wall rearrangements exposing β-glucan at the cell surface.

The observed sensitivity of *upc2*Δ cells to caspofungin in the presence of SNP+SHAM suggested that Upc2 links respiration to cell wall integrity. This led us to examine the effect of SNP+SHAM on chitin and β-glucan exposure in the *upc2*Δ mutant to determine whether Upc2 influences unmasking. The degree of surface chitin exposure in *upc2*Δ and the increase in unmasking upon SNP+SHAM treatment were not significantly different from levels seen with the wild type (Fig. 5E). Similarly, the results of dectin-1 staining for *upc2*Δ were also not significantly different from results seen with the wild-type strain (Fig. S1C). These results suggest that cell wall unmasking and the sensitivity to caspofungin in response to SNP+SHAM treatment may not share a mechanism.

Given that Upc2 was not linked to β(1,3)-glucan or chitin exposure, we tested a number of strains deleted for transcription factors that were identified as upregulated within our RNA-seq data in response to SNP+SHAM treatment. Our investigations did not identify deletions of any single transcription factor that could prevent β(1,3)-glucan or chitin exposure. However, deletion of *SKO1* led to an increase in chitin unmasking following SNP+SHAM exposure relative to the level seen with the wild-type strain (Fig. 5E). This may suggest that Sko1 acts as a repressor of chitin unmasking or simply that it is involved in the repair of cell wall rearrangements caused by this treatment.

We wished to examine whether other forms of respiration deficiency could lead to the same cell wall unmasking phenotype. The *ndh51*Δ mutant (7) does not possess a functional complex I and has a low respiration level. It showed a very high degree of chitin unmasking as determined by WGA staining (Fig. S2A). Dectin-1 staining was also significantly increased, although to a lesser degree than in SNP+SHAM-treated cells (Fig. S2B). These results demonstrate that inhibition of the classical electron transfer chain through the activity of either complex I or complex IV can cause unmasking of chitin and β(1,3)-glucan.

10.1128/mBio.02535-18.3 FIG S2 Surface exposure of chitin and β(1,3)-glucan was increased in the *ndh51*Δ mutant relative to the wild-type strain. Samples from overnight cultures of the wild-type and *ndh51*Δ cells were washed three times in PBS and stained with (A) FITC-WGA or (B) dectin-1. Staining of the cell wall was scored manually from microscope images (*n* = 3). Three independent experiments were analyzed in each case. Graphs show means ± standard deviations. Student’s *t* test was used to compare groups. **, *P* < 0.01; *, *P* < 0.05. Download FIG S2, PDF file, 0.4 MB. Copyright © 2019 Duvenage et al. 2019 Duvenage et al. This content is distributed under the terms of the Creative Commons Attribution 4.0 International license.

### Pretreatment with SNP+SHAM increased phagocytosis by macrophages.

**FIG 6:**
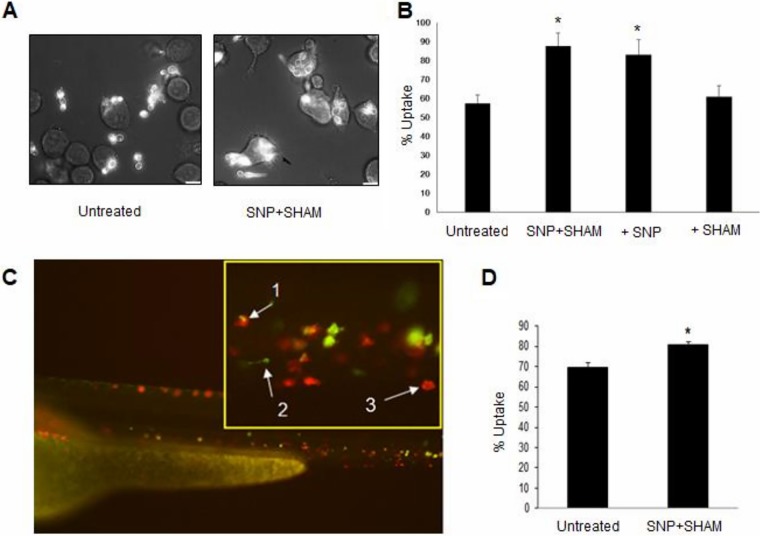
C. albicans pretreated with SNP+SHAM showed increased uptake by murine macrophages and by zebrafish macrophages *in vivo*. (A) Wild-type cells were treated with 1 mM SNP–0.5 mM SHAM for 18 h and then washed in phosphate-buffered saline (PBS) and coincubated with J774.1 murine macrophages (3:1 C. albicans/macrophage ratio). Representative examples of uptake after 1 h coincubation are shown. (B) Uptake of cells treated with SNP, SHAM, or SNP+SHAM was quantified by counting at least 200 C. albicans cells per experiment. *, *P* < 0.001. Five independent experiments were carried out. The percentage of uptake was determined as the number of internalized C. albicans cells relative to the total number of C. albicans cells. (C) Wild-type GFP-labeled C. albicans cells were treated with 1 mM SNP–0.5 mM SHAM for 18 h, washed, and injected into zebrafish larvae. A representative microscopy image of C. albicans (green) being taken up by macrophages (red) *in vivo* is presented (1, phagocytosed C. albicans; 2, single C. albicans cell; 3, single macrophage). (D) Proportions of phagocytosed C. albicans cells were calculated from images taken from three independent experiments (*n* = 3). *, *P* < 0.01. Graphs show means ± standard deviations. Student's *t* test was used to compare groups.

Due to the higher β(1,3)-glucan surface exposure in SNP+SHAM-treated cells, it was hypothesized that these cells may be recognized and engulfed more readily by macrophages. SNP+SHAM-treated cells were coincubated with J774.1 murine macrophages and were examined after 1 h by microscopy and scored for uptake. A higher proportion of the SNP+SHAM-pretreated cells than of untreated cells were taken up by macrophages after 1 h (Fig. 6A and B). In line with unmasking being linked to inhibition of classical respiration, uptake by macrophages was enhanced to the same degree upon SNP pretreatment (Fig. 6B). A similar increase in uptake was observed when cells were pretreated with 1 mM potassium cyanide (KCN) only (Fig. S3A), also suggesting that inhibition of the classical respiratory chain was responsible for this phenotype. In agreement with this, inhibition of the alternative respiration pathway alone by the use of SHAM or of the *aox2*Δ *aox1*Δ mutant had no significant effect on uptake (Fig. S3A). An increase in uptake of *ndh51*Δ cells was also observed, but the degree of increase was not the same as that seen with SNP+SHAM-pretreated cells (Fig. S3B). In order to understand how SNP+SHAM pretreatment would influence systemic candidiasis *in vivo* during infection, we used the zebrafish larva model, in which we could examine phagocytosis of C. albicans *in vivo*. C. albicans expressing green fluorescent protein (GFP) was pretreated with SNP+SHAM for 18 h. The pretreated cells were then injected into zebrafish larvae with fluorescently labeled macrophages. Z-stack images of each fish were taken using confocal microscopy, and uptake by macrophages was assessed manually. The uptake of pretreated cells was approximately 10% higher than the uptake of untreated cells (Fig. 6C and D).

10.1128/mBio.02535-18.4 FIG S3 Inhibition of classical ETC in C. albicans leads to enhanced uptake by murine macrophages. (A) Wild-type and *aox2*Δ *aox1*Δ C. albicans cells were grown in the presence of 1 mM KCN or 0.5 mM SHAM for 18 h as indicated. The cells were washed and coincubated with macrophages as described in Materials and Methods, and uptake was scored manually from microscope images taken after 1 h (*n* = 3). (B) The same procedure was applied to wild-type and *ndh51*Δ cell*s* (*n* = 3). Three independent experiments were analyzed in each case. Graphs show means ± standard deviations. Student’s *t* test was used to compare groups. *, *P* < 0.01. Download FIG S3, PDF file, 0.5 MB. Copyright © 2019 Duvenage et al. 2019 Duvenage et al. This content is distributed under the terms of the Creative Commons Attribution 4.0 International license.

### SNP+SHAM pretreatment increased the virulence of C. albicans in a mouse systemic model of candidiasis.

**FIG 7:**
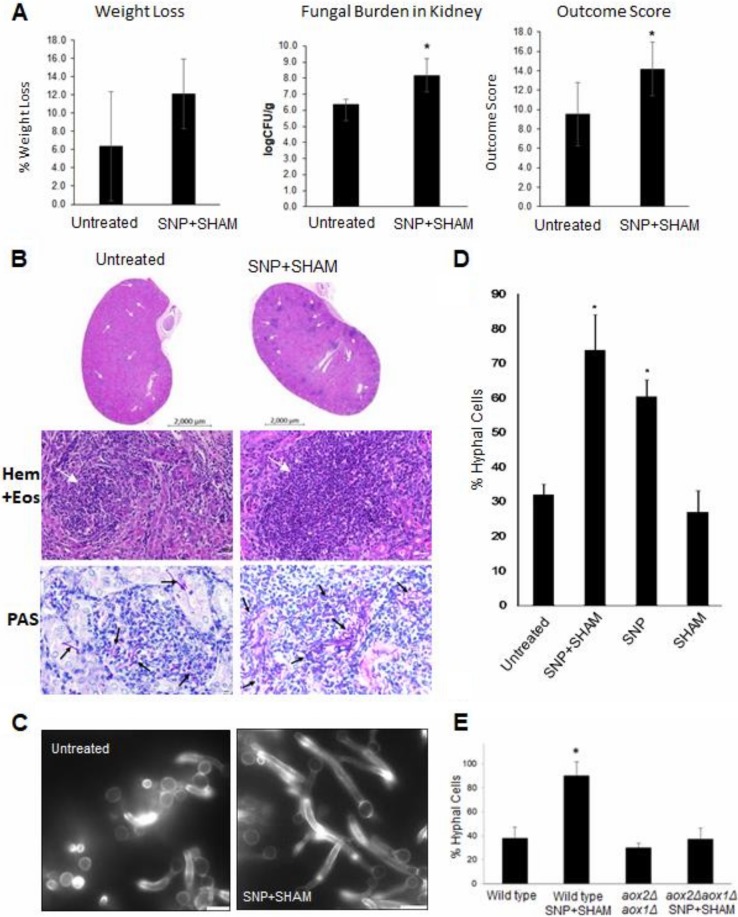
Pretreatment of C. albicans increases virulence in the mouse model and leads to more-rapid filamentation. (A) C. albicans cells were treated with SNP+SHAM for 18 h and then washed in PBS prior to injection via the tail vein. Weight loss, fungal burden, and outcome score were calculated as described in Materials and Methods. *, *P* < 0.05. (B) Cross-section from kidney infected with untreated wild-type or SNP+SHAM-pretreated cells, both stained with hematoxylin and eosin (Hem + Eos; bar, 50 µm) to emphasize locations of immune infiltration (white arrows) or periodic acid-Schiff reagent (PAS; bar, 20 µm) to highlight C. albicans *cells* (black arrows). (C) Wild-type cells were treated with 1 mM SNP–0.5 mM SHAM for 18 h and then washed in PBS and transferred to serum-containing medium at 37°C. Representative examples of filamentation in wild-type untreated and SNP+SHAM-pretreated cells after 90 min. (D) The percentages of filamentous cells were calculated in cells pretreated with SNP, SHAM, or SNP+SHAM, washed, and grown for 90 min as described for panel C (*n* = 3). (E) Wild-type and *aox2*Δ *aox1*Δ mutant cells were treated with 1 mM SNP–0.5 mM SHAM for 18 h and then washed in PBS and transferred to serum-containing medium at 37°C for 90 min before assessment. Graphs show means ± standard deviations. Three independent experiments were performed for each analysis. Student's *t* test was used to compare groups. *, *P* < 0.01.

It was not clear whether the increased uptake of SNP+SHAM-pretreated C. albicans by macrophages would attenuate virulence *in vivo.* To determine this, C. albicans were pretreated with SNP+SHAM before being washed and injected into the tail vein of 6-to-8-week-old female BALB/c mice, and the progression of infection was followed by monitoring weight loss and the condition of the animals. Kidney fungal burden measurements were made at 3 days postinfection to give an “outcome score.” A higher outcome score is indicative of greater weight loss and higher fungal burdens and thus of virulence. It was observed that the weight loss caused by SNP+SHAM-pretreated C. albicans was more rapid than that seen with the controls (Fig. 7A). Infection with SNP+SHAM-treated C. albicans cells also led to significantly higher fungal burdens within the kidney at the time of culling. The combination of these factors led to a significantly higher outcome score for SNP+SHAM-pretreated cells than for untreated cells (Fig. 7A). Histological analysis of kidney tissue from mice infected with cells pretreated with SNP-SHAM revealed an increase in immune infiltrate and fungal cell numbers compared to controls (Fig. 7B). The resulting data suggest that pretreatment with SNP+SHAM did not reduce the fitness of C. albicans in the host but in fact increased virulence in the murine model.

One possibility is that pretreatment of C. albicans with SNP+SHAM may cause a stress response which primes cells to activate traits associated with virulence when the pretreated C. albicans is injected into the host. The rapid induction of filamentation is generally associated with increased escape from macrophages and virulence (44, 45). To investigate this, the incidence of hyphal formation was assessed after 90 min of incubation in serum-containing media at 37°C for cells which had been pretreated with SNP+SHAM for 18 h. The majority of pretreated cells showed filamentation within 90 min, while the untreated cells were slower to transition to hyphae under these conditions (Fig. 7C and D). The activation of rapid filamentation was also observed when cells were treated with SNP alone but was not seen with SHAM-only treatment (Fig. 7D). These results suggest that respiratory inhibition may prime cells for hyphal growth and that this facilitates a rapid hyphal switch when the inhibitors are removed. In agreement with this, a similar effect was observed when cells were pretreated with cyanide (Fig. S4A and S4B in the supplemental material). Since both cyanide and NO induce alternative respiration, germination after SNP+SHAM treatment was examined in an *aox2*Δ *aox1*Δ mutant. The mutant did not show the same level of hyphal formation as the WT, suggesting that the alternative oxidase enzymes are required for the filamentation response upon inhibition of classical respiration (Fig. 7E). It is possible that the inhibition of the respiratory chain could lead to increased reactive oxygen species (ROS) production and that this might provide a signal that promotes hyphal switching. To investigate this, we assessed superoxide production and found that an increase could be detected upon SNP+SHAM treatment (Fig. S4C). The addition of N-acetyl cysteine was sufficient to prevent ROS accumulation but did not prevent the activation of filamentation in SNP+SHAM-treated cells (Fig. S4C and D). These data do not support the idea of a role for ROS production in linking the inhibition of respiration to filamentation.

10.1128/mBio.02535-18.5 FIG S4 Effects of ROS and cyanide on filamentation upon withdrawal of respiratory inhibition. (A) Cells were grown in the presence of 1 mM KCN–YPD for 18 h. Cells were then washed three times in PBS and transferred to Dulbecco’s modified Eagle’s medium (DMEM)–10% fetal bovine serum (FBS) at 37°C. After 90 min of incubation, the cells were examined for hyphal growth by microscopy. Representative examples are shown. (B) Summary of the percentages of filamentous cells from two independent experiments. (C) Wild-type C. albicans was pretreated with N-acetylcysteine (NAC) as described in Materials and Methods and assessed for superoxide production using dihydroethidium (DHE) (*n* = 3). (D) The effects of NAC on SNP+SHAM-induced hyphal induction were assessed as described in Materials and Methods (*n* = 3). Error bars represent standard deviations. *, *P* < 0.01. Download FIG S4, PDF file, 0.3 MB. Copyright © 2019 Duvenage et al. 2019 Duvenage et al. This content is distributed under the terms of the Creative Commons Attribution 4.0 International license.

## DISCUSSION

Mitochondrial function has been studied extensively in the model yeast Saccharomyces cerevisiae but has been a neglected area in the study of pathogenic fungi. Mitochondria have essential roles in pathogenicity (46) and antifungal drug resistance (3). The electron transport chain of C. albicans, consists of an alternative and parallel pathway in addition to the classical ETC (47); this allows electron flow to be rerouted should one of the pathways become blocked. Nevertheless, simultaneous inhibition of the classical and alternative pathways can inhibit respiration by up to 90% and severely restricts growth (48).

We have shown that NO exposure resulting from SNP treatment is effective in inhibiting respiration and activation of an alternative respiration response, as has been shown for other inhibitors of classical respiration (48). Inhibition of both classical and alternative respiration using SNP+SHAM led to sustained inhibition of respiration, suggesting that the level of NO released by SNP in our experiments was sufficient to irreversibly inhibit cytochrome *c* oxidase. Peroxynitrite, which can be formed when NO reacts with endogenous superoxide, has also been shown to cause irreversible inhibition of cytochrome *c* oxidase (49).

SNP+SHAM treatment showed a synergistic effect with cell wall-damaging agents, calcofluor white, and Congo red. C. albicans respiration-deficient mutants *dpb4*Δ, *hfl1*Δ, and *rbf1*Δ (8) and mutants *goa1*Δ and *ndh51*Δ (16) have similarly been shown to be sensitive to CFW and CR. A number of genes found to be differentially expressed upon SNP+SHAM treatment have been reported to be involved in the response to caspofungin. Genes induced in this response include *AGP2*, *KRE1*, *PGA13*, *RHR2*, and *CZF1*; repressed genes include *CHT2*, *PGA4* and *SCW11*, *HAS1*, *CRZ1*, and *ENG1* (50–52). These expression changes may help to explain why SNP+SHAM-treated cells were more resistant to caspofungin.

Growth in the presence of SNP+SHAM caused an increase in the surface exposure of chitin and β(1,3)-glucan. Sherrington et al. showed that unmasking of β(1,3)-glucan and chitin occurred during adaptation to low pH (15).Those authors found no activation of Mkc1 kinase involved in the cell wall integrity response and reported increased uptake by macrophages, both of which were also seen in SNP+SHAM-induced unmasking. Masking of β(1,3)-glucan has been shown to occur during growth on lactate, which aids in immune evasion. (14). It was found that Crz1 was required for β(1,3)-glucan masking and that *pga26*Δ and *cht2*Δ mutants showed elevated β(1,3)-glucan exposure. These and other genes encoding cell wall proteins were found to be downregulated in SNP+SHAM-treated cells, suggesting repression of glucan masking at the level of Crz1 but also a shift in the cell wall protein profile, promoting elevated β(1,3)-glucan exposure (Fig. 4B).

The role of mitochondria in maintaining phospholipid homeostasis was previously shown to be important for cell wall integrity (53). Genes involved in the synthesis of phosphatidylserine (PS) (*CHO1*) and linoleic acid (*FAD3* and *FAD2*) were also downregulated in SNP+SHAM-treated cells. The *cho1*Δ mutant, which is defective in PS synthesis, was shown to exhibit unmasking of β(1,3)-glucan (54), and *CHO1* is also essential for cell wall integrity (55). Treatment of cells with SNP+SHAM also led to the downregulation of a group of ergosterol biosynthesis pathway genes (Fig. 4B). Inhibition of sterol biosynthesis has been shown to lead to an increase in the incidence of lipid droplets (56), which we observed in SNP+SHAM-treated cells. *UPC2*, encoding a transcription factor known to activate ergosterol biosynthesis, especially under hypoxic conditions, was upregulated and required for the observed resistance to caspofungin. Decreased ergosterol production has previously been observed in respiration-deficient C. albicans mutants (57–59). An increase in *UPC2* expression might be a compensatory response to reduced ergosterol production, or, alternatively, SNP+SHAM treatment might mimic hypoxia, a condition that also leads to elevated Upc2 levels. In agreement with this, we observed a significant overlap in differential expression of genes with SNP+SHAM treatment and published microarray data produced under conditions of hypoxia (see Fig. S5 in the supplemental material) (60). However, two recent studies clearly indicated that hypoxic conditions led to alterations in the cell wall that mask C. albicans from host responses (61, 62). This suggests that respiration inhibition and hypoxia lead to changes in cell wall architecture via independent mechanisms. Shorter outer cell wall fibrils were seen in the respiration-deficient *goa1*Δ mutant, similarly to those observed in SNP+SHAM-treated cells. The cell wall alterations in the *goa1*Δ mutant included a reduction in the levels of high- and intermediate-molecular weight mannans (17), but no significant changes in the relative levels of glucan, chitin, or mannan were observed in SNP+SHAM-treated cells. In addition, the reduced recognition of the *goa1*Δ mutant by macrophages, which contrasts with the results seen in SNP+SHAM-treated cells, suggested that the cell wall changes resulting from transient respiration inhibition were distinct from those seen in the *goa1*Δ mutant.

10.1128/mBio.02535-18.6 FIG S5 Comparison of transcriptomes of SNP+SHAM-treated cells and cells in early hypoxia. Differentially expressed genes induced by SNP+SHAM treatment were compared to those identified within microarray data by Sellam et al. (60) by examining the early response to hypoxia, using data from the 30-min time point. Selected genes common to both datasets are highlighted. Up and down arrows indicate their upregulation and downregulation, respectively. Download FIG S5, PDF file, 0.3 MB. Copyright © 2019 Duvenage et al. 2019 Duvenage et al. This content is distributed under the terms of the Creative Commons Attribution 4.0 International license.

Despite unmasking leading to an increase in immune cell recognition, SNP+SHAM pretreatment led to enhanced virulence in a mouse model, with a clear increase in immune cell infiltrate and yeast cell numbers within the kidney (Fig. 7B). Exposure of cell wall β-glucan has been shown to increase the inflammation response (63). One possibility, therefore, is that SNP+SHAM pretreatment may have led to an increased and damaging inflammation response. Another possibility rests with the activation of hyphal switching that we observed upon removal of SNP+SHAM. Enhanced germination may have facilitated escape from macrophages as well as damage to epithelial barriers, aiding dissemination of the infection. It might have been the case that release from respiratory inhibition (as the SNP+SHAM-treated cells were washed prior to injection) led to an increase in activation of Ras1, a key regulator of hyphal transition. This hypothesis is supported by observations revealing that inhibition of respiration decreases Ras1 activation, which prevents hyphal switching (9). Reduced Ras1 activity in response to inhibition of respiration is correlated with a reduction in ATP levels; this did not appear to be sensed by AMPK (9). The mechanism connecting mitochondrial inhibition and Ras1 activation is unknown. The absence of an increase in hyphal switching in SNP+SHAM-pretreated *aox2*Δ *aox1*Δ mutant cells suggests that an increased level of Aox2 could be important for the increased switch to hyphae in response to respiratory stress. This observation, together with previous evidence that the alternative pathway is more active in hyphal cells (64), warrants further investigation into the precise role of Aox2 in morphogenetic switching.

In addition to hyphal growth regulators, several other virulence-related genes were upregulated by SNP+SHAM treatment. For example, Tsa1 (thioredoxin peroxidase 1), which provides resistance to reactive nitrogen species and has also been shown to contribute to fungal virulence (65), was among the most highly upregulated genes following SNP+SHAM treatment. Other upregulated genes associated with pathogenesis included *SAP6* (66), *GLK1* and *PHO112* (67), and *PLB1* (68) and the transcription factor *GAT1* as well as its regulation targets *DUR1*,*2* and *MEP2* (69). The induction of these genes, several of which are normally associated with the hyphal form of C. albicans, suggests that SNP+SHAM treatment may prime cells for increased virulence, which could manifest upon withdrawal of the treatment.

In summary, our study results suggest that inhibition of respiration as an antifungal strategy in C. albicans is a complex proposition. Respiration inhibition with SNP+SHAM causes cell wall unmasking, which leads to enhanced recognition by macrophage cells but primes a stress response leading to enhanced filamentation and increased virulence when inhibition is removed. We would argue that the development of a mitochondrial inhibition strategy, while still an attractive goal given the importance of the organelle for C. albicans growth, requires a better understanding of the signaling properties of this organelle under conditions of stress.

## MATERIALS AND METHODS

A detailed description of the strains and of the materials and methods used in this study can be found in Text S1 in the supplemental material.

10.1128/mBio.02535-18.1 TEXT S1 Supplemental Materials and Methods. Download Text S1, DOCX file, 0.04 MB. Copyright © 2019 Duvenage et al. 2019 Duvenage et al. This content is distributed under the terms of the Creative Commons Attribution 4.0 International license.

### C. albicans growth conditions and chemicals.

The C. albicans strains used in this study are identified in Table S1 in the supplemental material. Growth and manipulation conditions are detailed within Text S1. The *aox2*Δ *aox1*Δ and *aox2*Δ mutant strains used were constructed as detailed within Text S1.

10.1128/mBio.02535-18.7 TABLE S1 Strains used in this study. All yeast strains used in this study and their origins are listed. Download Table S1, PDF file, 0.1 MB. Copyright © 2019 Duvenage et al. 2019 Duvenage et al. This content is distributed under the terms of the Creative Commons Attribution 4.0 International license.

10.1128/mBio.02535-18.8 TABLE S2 List of genes differentially expressed upon SNP treatment. Cells were treated with 1.0 mM SNP for 30 min and processed for RNA sequencing and downstream analysis as described in Materials and Methods. A full list of differentially expressed genes is provided. Download Table S2, PDF file, 0.1 MB. Copyright © 2019 Duvenage et al. 2019 Duvenage et al. This content is distributed under the terms of the Creative Commons Attribution 4.0 International license.

10.1128/mBio.02535-18.9 TABLE S3 List of genes differentially expressed upon SHAM treatment. Cells were treated with 0.5 mM SHAM for 30 min and processed for RNA sequencing and downstream analysis as described in Materials and Methods. A full list of differentially expressed genes is provided. Download Table S3, PDF file, 1.3 MB. Copyright © 2019 Duvenage et al. 2019 Duvenage et al. This content is distributed under the terms of the Creative Commons Attribution 4.0 International license.

10.1128/mBio.02535-18.10 TABLE S4 List of genes differentially expressed upon SNP+ SHAM treatment. Cells were treated with 1.0 mM SNP and 0.5 mM SHAM for 30 min and processed for RNA sequencing and downstream analysis as described in Materials and Methods. A full list of differentially expressed genes is provided. Download Table S4, PDF file, 1.1 MB. Copyright © 2019 Duvenage et al. 2019 Duvenage et al. This content is distributed under the terms of the Creative Commons Attribution 4.0 International license.

### Whole-cell respirometry.

Respirometry was carried out in real time using an Oxygraph-2k respirometer (Oroboros Instruments, Austria) as detailed in Text S1. Data were analyzed using Datlab 6 software (Oroboros Instruments). Six independent experiments were performed.

### Viability and susceptibility assays.

Analysis of viability and sensitivity to cell wall-disrupting agents or antifungal drugs was carried out as described in Text S1. In all cases, three independent experiments were performed.

### RNA isolation and RNA-seq.

C. albicans SC5314 was grown for 5 h in yeast extract-peptone-dextrose (YPD) at 30°C in a shaking incubator. SNP and SHAM were added to reach final concentrations of 1 mM and 0.5 mM, respectively, and the cells were returned to the incubator for a further 30 min. RNA was then extracted and prepared for RNA sequencing as described in Text S1. Raw RNA-seq data were analyzed using the suite of tools available on the Galaxy platform; for full details, see Text S1.

### Cell wall staining and analysis.

Cells from an overnight culture in YPD were diluted in YPD to a final optical density at 600 nm (OD_600_) of 0.2 and grown for 5 h at 30°C. SNP (1 mM) and SHAM (0.5 mM) were added to the cultures, and they were grown for a further 18 h. The cells were processed for wheat germ agglutinin or dectin-1 staining as described in Text S1. At least 400 cells were counted for each experiment, and three independent experiments were performed per analysis. Cell wall composition was analyzed by high-performance anion-exchange chromatography as described in Text S1.

### Western blotting.

Western blotting was carried out as described in Text S1. Phosphorylated Hog1 or Mkc1 was detected using p44/p42 (1:2,000) or p38 (1:1,000) monoclonal antibodies (Cell Signaling Technology catalog no. 4695 and 8690, respectively). A monoclonal antibody (AS10 699, Agrisera, Sweden) (1:100) against *Sauromatum guttatum* Aox which recognizes C. albicans Aox2 was used for Aox immunoblotting. Actin was detected using an antibody raised against S. cerevisiae actin (a kind gift from John Cooper, University of Washington) at a 1:1,000 dilution.

### Phagocytosis assay.

Uptake of C. albicans by J774.1 murine macrophages was carried out as described in Text S1. The percentage of uptake was determined as the number of internalized C. albicans cells relative to the total number of C. albicans cells. At least 200 C. albicans cells were counted for each experiment. Five independent experiments were performed.

### Hyphal induction.

C. albicans hyphal induction assays were carried out as described in Text S1. The percentage of hyphal cells was determined manually using ImageJ. A total of least 500 cells were counted for each experiment.

### Zebrafish *in vivo* imaging.

Imaging of C. albicans uptake within 2-day-old zebrafish was carried out as described in Text S1. Assessment of C. albicans uptake by macrophages was performed manually using NIS-Elements Viewer 4.20 (Nikon, Richmond, United Kingdom). Three independent experiments were performed using 10 larvae per condition.

### Electron microscopy.

Imaging of C. albicans sections by transmission electron microscopy was carried out as described in Text S1. ImageJ was used to manually measure the thicknesses of the inner (i) and outer (o) cell walls (as indicated in Fig. 2B). Five measurements were taken along the cell wall for each cell, for a total of 25 cells per group.

### Lipid droplet staining.

LD540 lipid droplet stain was used as described in Text S1.

### Murine C. albicans systemic infection model.

The effect of SHAM+SNP pretreatment of C. albicans was evaluated using a murine intravenous challenge assay as described in the supplemental materials. Mouse kidneys were cryosectioned and analyzed as described in Text S1.

### Data accessibility.

The data discussed in this publication have been deposited in NCBI's Gene Expression Omnibus and are accessible through GEO Series accession number GSE114531 (https://www.ncbi.nlm.nih.gov/geo/query/acc.cgi?acc=GSE114531).
