# Supplementary material for: Inhibition of Classical and Alternative Modes of Respiration in Candida albicans Leads to Cell Wall Remodeling and Increased Macrophage Recognition
Source: mBio. 2019 Jan 29;10(1):e02535-18. doi: 10.1128/mBio.02535-18 (PMC6355986; doi:10.1128/mBio.02535-18)
Supplement: FIG S1 [file mBio.02535-18-sf001.pdf]

Supplementary Figure S1

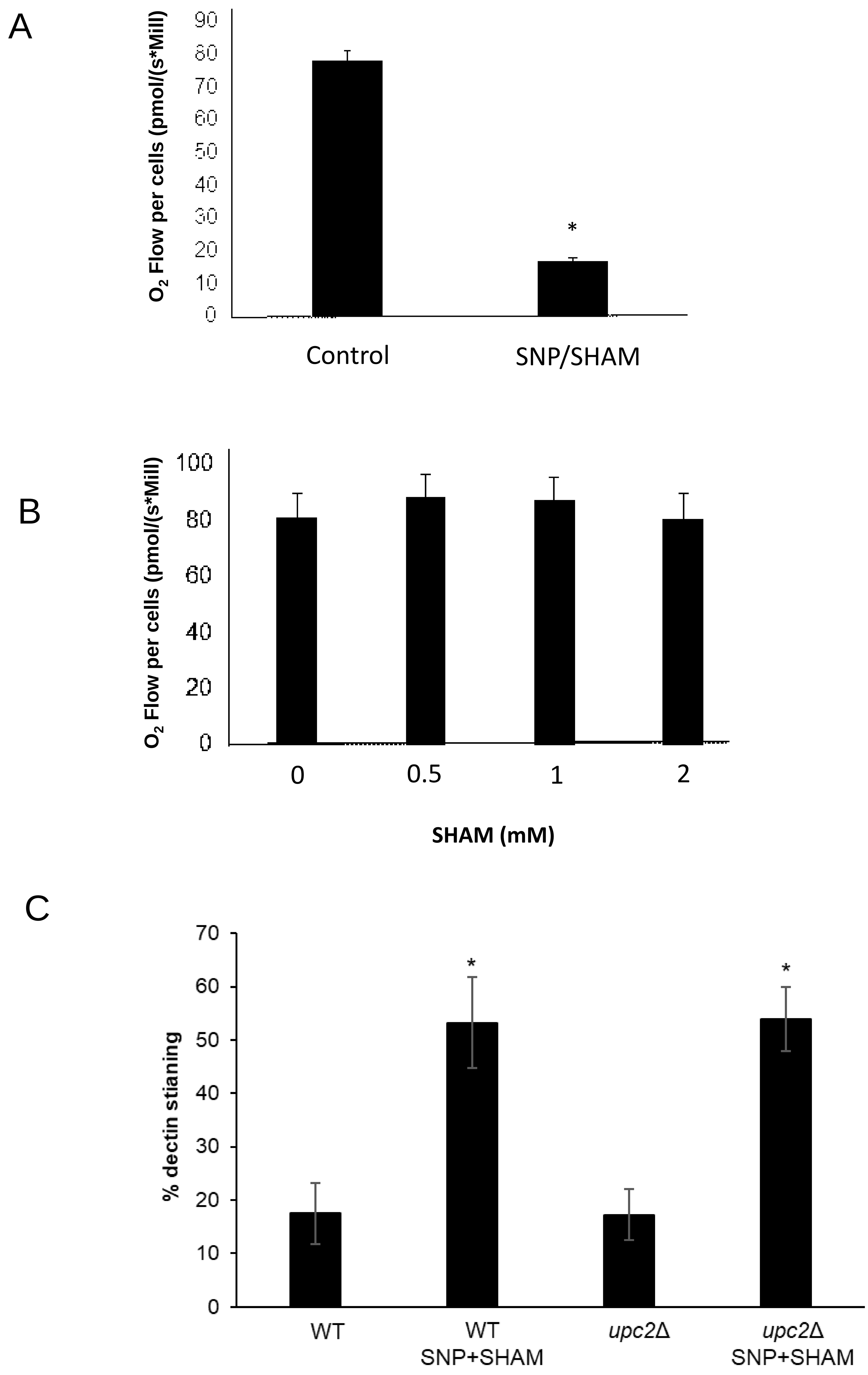

Fig S1. SNP+SHAM induces surface exposure of β(1,3)-glucan in both the wild-type and *upc2Δ*

(A) Respiration was assessed in *C. albicans* cells prior to and following addition of 4mM SNP + SHAM and the resultant respiration levels are given, n=3 . (B) Respiration in whole *C. albicans* cells was determined using high-resolution respirometry. SHAM was added at 0.5, 1 and 2 mM concentrations in sequential doses and the resultant respiration is given. (C) Wild type and *upc2Δ* mutant cells were treated with 1 mM SNP + 0.5 mM SHAM for 18 h and stained with dectin-1 as described in materials and methods. Dectin-1 staining of the cell wall was assessed manually from microscopy images, n=3. Three independent experiments were analysed. Graphs show means ± standard deviation. Student's t-test was used to compare groups, * $p<0.01$
