# Supplementary material for: Inhibition of Classical and Alternative Modes of Respiration in Candida albicans Leads to Cell Wall Remodeling and Increased Macrophage Recognition
Source: mBio. 2019 Jan 29;10(1):e02535-18. doi: 10.1128/mBio.02535-18 (PMC6355986; doi:10.1128/mBio.02535-18)
Supplement: FIG S2 [file mBio.02535-18-sf002.pdf]

Supplementary Figure S5

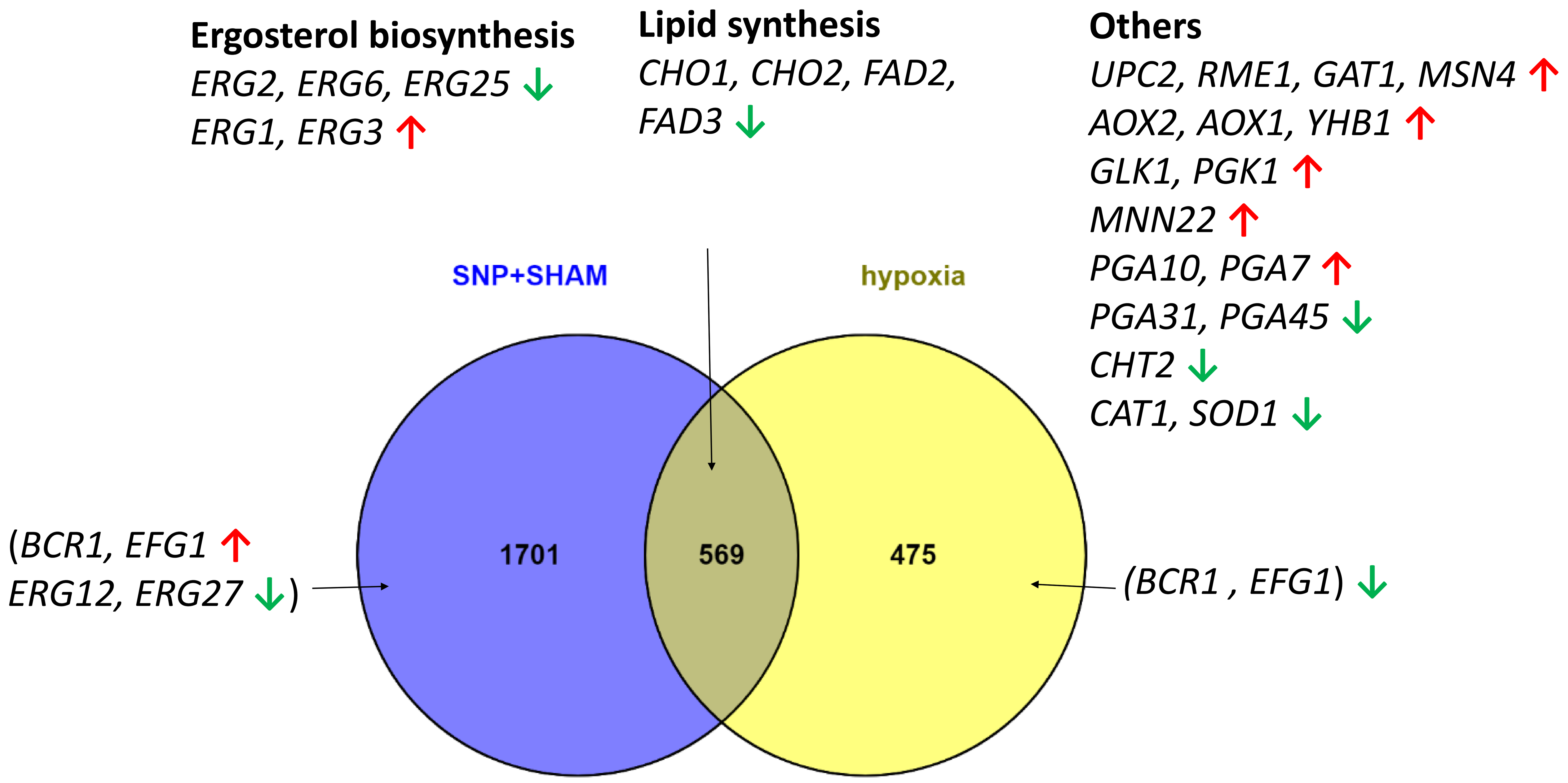

Fig S5. Comparison of transcriptomes of SNP+SHAM treated cells and cells in early hypoxia.

Differentially expressed genes induced by SNP+SHAM treatment were compared to those identified within microarray data by Sellam et al. [69] examining the early response to hypoxia, using data from the 30 min time point. Selected genes common to both datasets are highlighted. Up- and down arrows indicate their up- or downregulation respectively.
