# Supplementary material for: Inhibition of Classical and Alternative Modes of Respiration in Candida albicans Leads to Cell Wall Remodeling and Increased Macrophage Recognition
Source: mBio. 2019 Jan 29;10(1):e02535-18. doi: 10.1128/mBio.02535-18 (PMC6355986; doi:10.1128/mBio.02535-18)
Supplement: FIG S3 [file mBio.02535-18-sf003.pdf]

Supplementary Figure S4

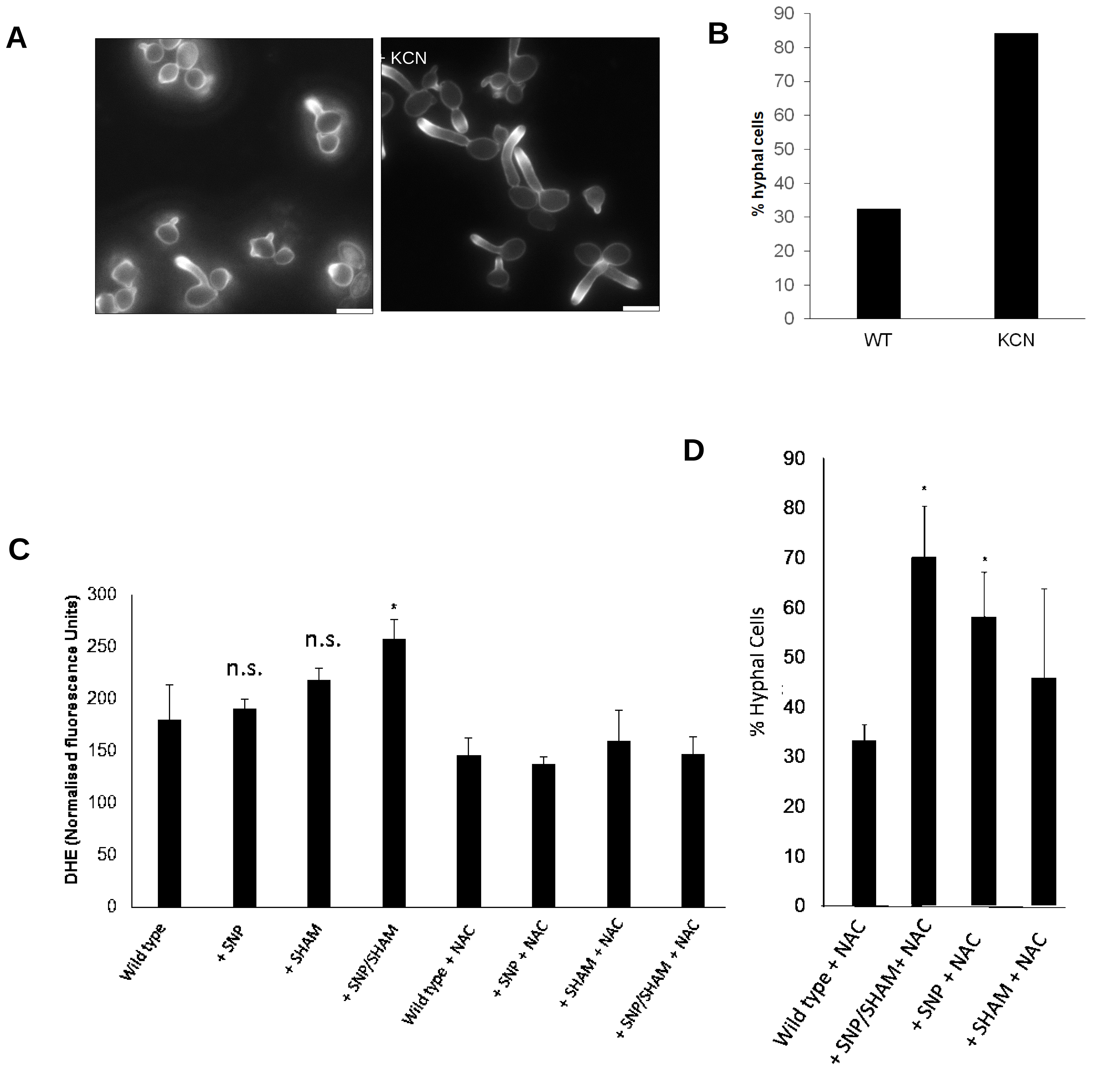

Fig S4. Effects of ROS and cyanide upon filamentation upon withdrawal of respiratory inhibition
(A) Cells were grown in the presence of 1 mM KCN in YPD for 18 h. Cells were then washed three times in PBS and transferred to DMEM + 10% FBS at 37 °C. After 90 min incubation, the cells were examined for hyphal growth by microscopy. Representative examples are shown**. (B)** Summary of the percentage of filamentous cells from two independent experiments. **(C)** Wild-type *C. albicans* were pre-treated with N-acetylcysteine (NAC) as described in materials and methods and assessed for superoxide production using DHE, n =3. **(D)** The effects of NAC upon SNP/SHAM induced hyphal induction was assessed as described in materials and methods, n =3. Error bars represent standard deviation, * = P<0.01.
