# Supplementary material for: Inhibition of Classical and Alternative Modes of Respiration in Candida albicans Leads to Cell Wall Remodeling and Increased Macrophage Recognition
Source: mBio. 2019 Jan 29;10(1):e02535-18. doi: 10.1128/mBio.02535-18 (PMC6355986; doi:10.1128/mBio.02535-18)
Supplement: FIG S4 [file mBio.02535-18-sf004.pdf]

Supplementary Figure S3

A

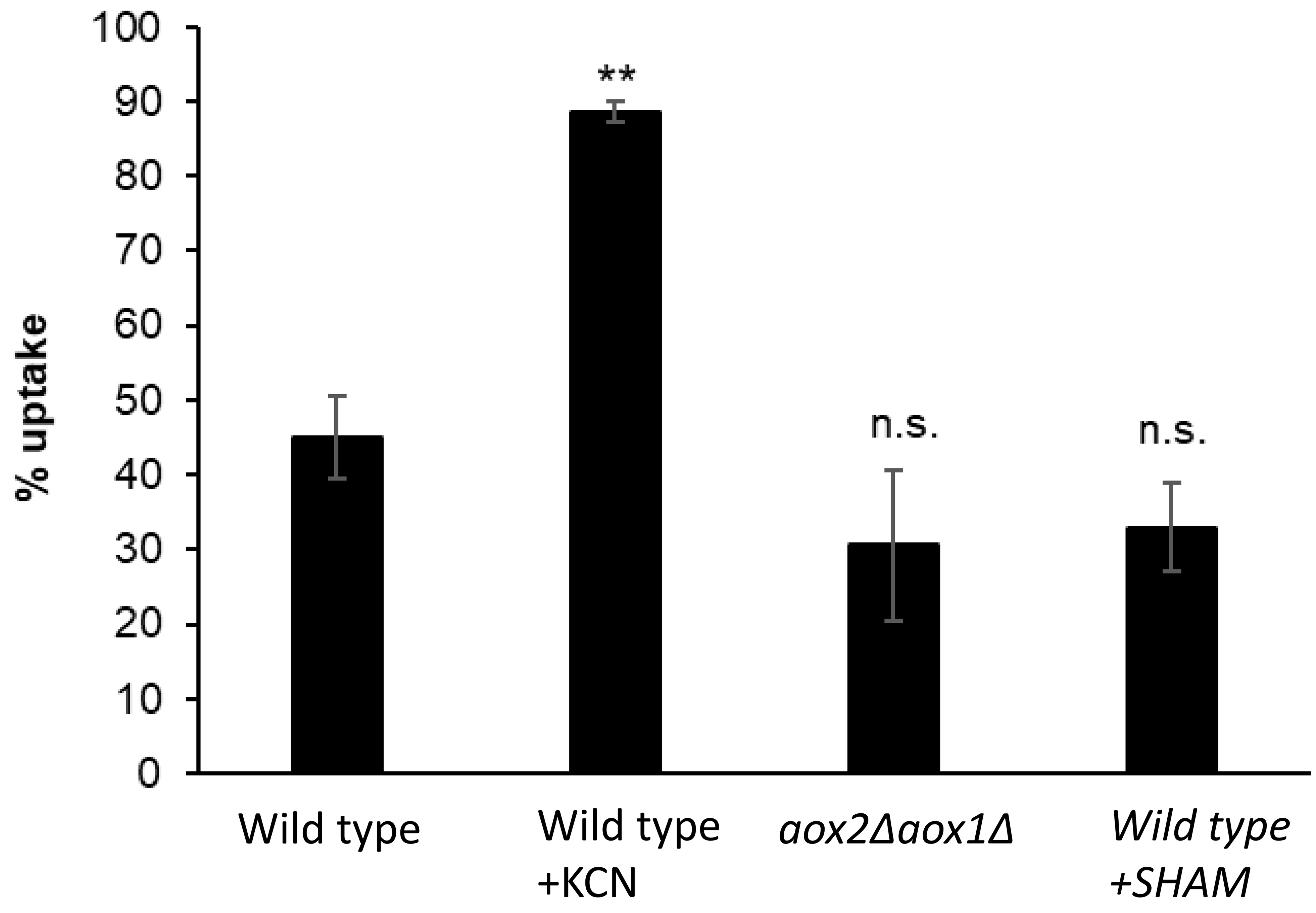

B

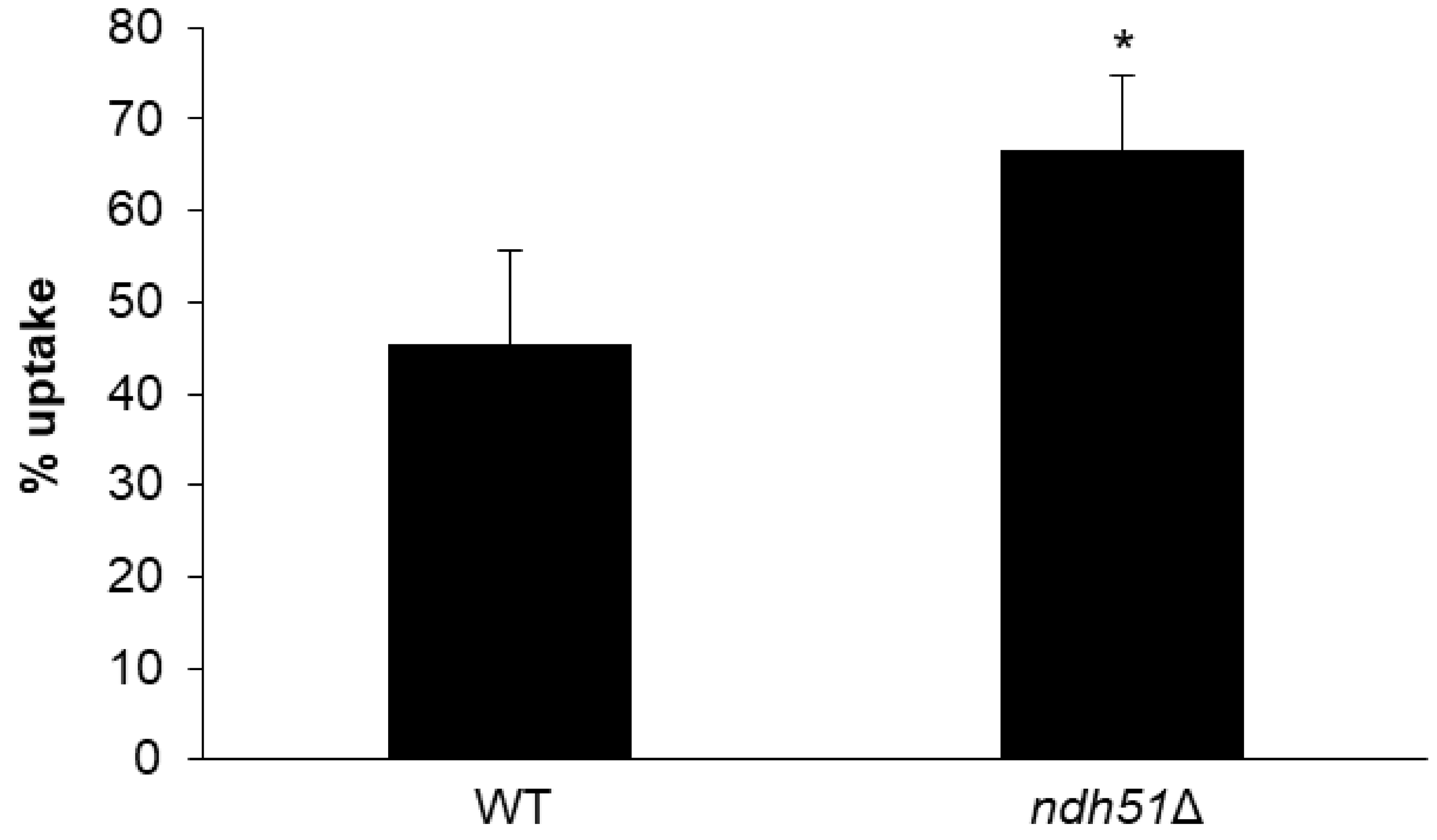

Fig S3. Inhibition classical ETC in *C. albicans* leads to enhanced uptake by murine macrophages

(A) Wild-type and *aox2-aox1*Δ *C. albicans* cells were grown in the presence of 1 mM KCN or 0.5 mM SHAM for 18 h as indicated. The cells were washed and co-incubated with macrophages as described in materials and methods and uptake scored manually from microscope images taken after 1 h, n=3. **(B)** The same procedure was applied to wild type and *ndh51*Δ cell*s,* n=3. Three independent experiments were analysed in each case. Graphs show means ± standard deviation. Student's t-test was used to compare groups, * $p<0.01$
