# Supplementary material for: Inhibition of Classical and Alternative Modes of Respiration in Candida albicans Leads to Cell Wall Remodeling and Increased Macrophage Recognition
Source: mBio. 2019 Jan 29;10(1):e02535-18. doi: 10.1128/mBio.02535-18 (PMC6355986; doi:10.1128/mBio.02535-18)
Supplement: FIG S5 [file mBio.02535-18-sf005.pdf]

Supplementary Figure S2

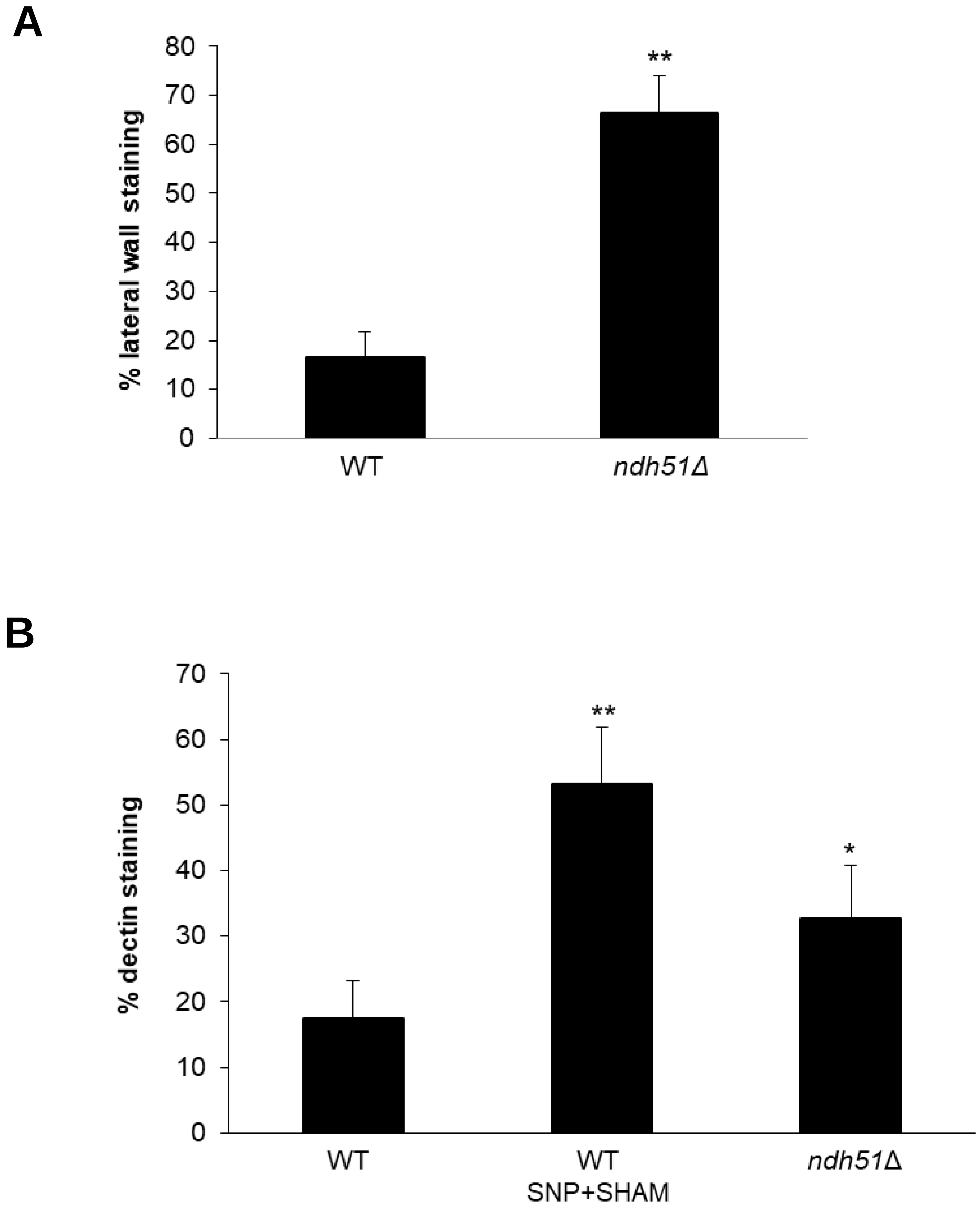

Fig S2. Surface exposure of chitin and β(1,3)-glucan is increased in *ndh51*Δ relative to the wild-type strain.

Samples from overnight cultures of the wild-type and *ndh51*Δ cells were washed three times in PBS and stained with **(A)** FITC-WGA or **(B)** dectin-1. Staining of the cell wall was scored manually from microscope images, n=3. Three independent experiments were analysed in each case. Graphs show means ± standard deviation. Student's t-test was used to compare groups, $^{**}p<0.01$, $^{*}p<0.05$
