# Supplementary material for: Inhibition of Classical and Alternative Modes of Respiration in Candida albicans Leads to Cell Wall Remodeling and Increased Macrophage Recognition
Source: mBio. 2019 Jan 29;10(1):e02535-18. doi: 10.1128/mBio.02535-18 (PMC6355986; doi:10.1128/mBio.02535-18)
Supplement: TEXT S1 [file mBio.02535-18-s0001.docx]

**Materials and methods**

***C. albicans* growth conditions and chemicals**

*C. albicans* strains were maintained on YPD agar plates and grown in YPD in a 30 °C shaking incubator unless stated otherwise. The concentrations of sodium nitroprusside dihydrate (SNP) and salicylhydroxamic acid (SHAM) (Cat. No. 1.06541 and S607, Sigma-Aldrich, Dorset, UK) used for inhibition were 1 mM and 0.5 mM respectively, added to log phase cells followed by 18 h growth unless stated otherwise. SNP was dissolved in water and SHAM was dissolved in ethanol. Potassium cyanide, Calcofluor White, Congo Red and caspofungin diacetate (Cat. No. SML0425) were obtained from Sigma-Aldrich.

The transcription factor deletion library used in this study was constructed by Homann et al. (1). Most of the mutants screened, including the *sko1*Δ mutant, were derived from this library unless stated otherwise. The *cek1*Δ mutant was obtained from the deletion library described in (2). The *upc2*Δ mutant was kindly provided by Prof. Joachim Morschhäuser (3). The *aox2*Δ*aox1*Δ mutant was constructed from SN87 using the strategy described in (4). Briefly, *LEU2* and *HIS1* were amplified from plasmids pSN40 and pSN52 respectively, using universal primers with 80 bases homologous to the 5’ end of *AOX2* and the 3’ end of *AOX1* ORF’s. This strategy was designed to delete both *AOX2* and *AOX1* simultaneously as well as the region between these two adjacent genes. The primers used were AOX2-UP2: 5’ atgcttactgcttcgctttacaaacaattaccggtgttaaccaccacagctacttcaacatattctttcattagattatcACCAGTGTGATGGATATCTGC 3’ and AOX1-UP5: 5’ CtaaagatacaaatcctttctttcccatccttggggtctagttacatctaaattgtaatttggttgtggcttgtctgaatAGCTCGGATCCACTAGTAACG 3’. The PCR products were then transformed sequentially into *C. albicans* SN87 using an electroporation protocol (5), followed by selection on agar plates containing Yeast Nitrogen Base without amino acids supplemented with 2% glucose and -His or -Leu dropout powder (Formedium, UK) as appropriate. A summary of strains used in this study is shown in Supplementary Table S1.

**Whole cell respirometry**

Respirometry was carried out in real time using an Oxygraph-2k respirometer (Oroboros Instruments, Austria) which was calibrated at 30 °C as per the manufacturer’s instructions. Cells from an overnight culture in YPD were added to 3 ml fresh YPD to a final optical density at 600 nm (OD_600_) of 0.2. The cells were incubated at 30 °C with shaking for 2 h. The cells were then counted and diluted in YPD to give a final cell concentration of 1 x 10^6^ cells/ml, of which 2.5 ml was added to each chamber in the respirometer. Routine respiration refers to the respiration level immediately prior to the addition of inhibitors. SNP was added to a final concentration of 1 mM, followed by a second addition to a final concentration of 2 mM after 20 min. SHAM was added to a final concentration of 1 mM followed by a second addition 10 min later to give a final concentration of 2 mM. Lastly, potassium cyanide was added to give a final concentration of 2 mM. Data was analysed using Datlab 6 software (Oroboros Instruments). Six independent experiments were performed.

**Viability assays**

Cells from an overnight culture in YPD were diluted in YPD to a final OD_600_ of 0.2. The cells were grown for 5 hours at 30 °C with shaking. SNP and SHAM were added to a final concentration of 1 mM and 0.5 mM respectively and cultures were grown for a further 18 h. The cells were collected, washed three times in PBS and counted using a haemocytometer, then diluted in PBS. Four hundred cells of each suspension were plated on YPD agar plates. The plates were incubated for 24 h and the number of colony forming units (CFUs) were counted and compared to untreated controls. Three independent experiments were performed.

**Cell wall agent susceptibility assay**

YPD plates were prepared containing either 1 mM SNP, 0.5 mM SHAM, 25 µg/ml Calcofluor White, 50 µg/ml Congo Red, or combinations of these as stated. Cells from an overnight culture in YPD were washed three times in PBS and diluted in PBS to a final OD_600_ of 0.2. Cells were serially diluted (1:10 dilutions) and equal volumes were spotted on the plates using a replica plating tool. The plates were incubated for 48 h at 30 °C and photographed.

**Antifungal susceptibility assays**

A library of transcription factor mutants (1) was screened using a combination of 100 ng/ml caspofungin, 1 mM SNP and 0.5 mM SHAM in synthetic complete medium, 2 % glucose, 50 mM MOPS pH 6. Each strain was grown overnight in YPD at 30 °C, then washed 3 times in PBS. The OD_600_ was measured and used to inoculate 0.5 ml in 48 well plates to achieve a final OD_600_ of 0.1. Growth of the strains was monitored at 30 °C using a BMG labtech SPECTROstar Nano plate reader with orbital shaking at 400 rpm.

Microdilution assays were carried out in 96-well plates with caspofungin, with or without 1 mM SNP + 0.5 mM SHAM, using synthetic complete medium, 2 % glucose, 50 mM MOPS pH 6. A dilution series of caspofungin was made using the appropriate diluent between 3.9 ng/ml – 8 µg/ml. *C. albicans* cells from an overnight YPD culture were washed three times in PBS. The OD_600_ was measured and adjusted to 2.0. This cell suspension was diluted 1:100 into the appropriate diluent. One hundred microliters of cell suspension was added to the 100 µl in each well of the dilution series. The plates were incubated at 30 °C for 24 h without shaking before determining caspofungin MIC_80_ (the drug concentration needed to decrease the OD_600_ by 80% compared to untreated cells). Three independent experiments were performed.

**RNA isolation and RNAseq**

*C. albicans* SC5314 was grown for 5 h in YPD at 30 °C in a shaking incubator. SNP and SHAM were added to final concentrations of 1 mM and 0.5 mM respectively and the cells were returned to the incubator for a further 30 min. RNA was then extracted using a E.Z.N.A.® Yeast RNA Kit (Omega Bio-tek, Norcross, GA) following the manufacturer’s instructions, for three biological replicates per group. RNA was sent to the Centre for Genome Enabled Biology and Medicine (Aberdeen, UK), who performed preparation of stranded TruSeq mRNA libraries, QC/quantification and equimolar pooling, and sequencing on an Illumina NextSeq500 with 1x75bp single reads and average depth of 30M reads per sample. Raw RNAseq data was analysed using the suite of tools available on the Galaxy platform (6). Briefly, reads were aligned to Assembly 21 of the *C. albicans* genome (*Candida* Genome Database (7)) using HISAT2. Differentially expressed genes between untreated and treated samples were identified using Cuffdiff v2.1.1 (8). The p-values generated by Cuffdiff’s statistical algorithm were adjusted using Benjamini-Hochberg correction for multiple-testing to generate the q-value (allowed false discovery rate of 0.05). A q-value less than 0.05 was considered statistically significant. The data discussed in this publication have been deposited in NCBI's Gene Expression Omnibus and are accessible through GEO Series accession number GSE114531 (<https://www.ncbi.nlm.nih.gov/geo/query/acc.cgi?acc=GSE114531>).

**Cell wall staining**

Cells from an overnight culture in YPD were diluted in YPD to a final OD_600_ of 0.2 and grown for 5 h at 30 °C. 1 mM SNP and 0.5 mM SHAM were added to the cultures and they were grown for a further 18 h. The cells were collected and washed three times in PBS. Cells were then stained with 25 µg/ml Wheat Germ Agglutinin, Alexa Fluor™ 594 Conjugate (Cat. No. W11262, Thermo Fisher Scientific, Waltham, MA) in PBS at room temperature for 1 h in the dark. After washing three times in PBS, the cells were examined using a RFP filter with low-level brightfield illumination with an Olympus IX81 inverted microscope illuminated using a CoolLED pE-4000 unit and captured using an ANDOR Zyla 4.2 CMOS camera. For dectin-1 staining, the cells were incubated with 5 µg/ml Dectin-1-Fc (a kind gift from Prof. Gordon Brown, MRC Centre for Medical Mycology, at the University of Aberdeen) in PBS, 2% BSA for 1 hour at 4 °C. The cells were washed three times in PBS and incubated with sheep anti-human antibody-FITC (Thermo Fisher Scientific, Cat. No. PA5-16924) for 2 h at 4 °C. After washing three times in PBS, the cells were examined by microscopy using a GFP filter with low-level brightfield illumination. WGA and dectin-1 staining of the cell wall were manually assessed using ImageJ v1.50 (NIH, Bethesda, MD). To calculate the percentage of cells with lateral wall staining by WGA, staining of bud scars was ignored. Dectin-1 staining of the cell wall was either scored manually per cell or analysed based on mean fluorescence. To calculate this corrected total cell fluorescence, the background measurement was subtracted from the total integrated density for each whole microscopy image. This result was then divided by the total number of cells in the image. At least 400 cells were counted for each experiment and three independent experiments were performed per analysis.

**Western blotting**

To monitor the activation of Hog1 or Mkc1 by SNP+SHAM treatment, cells from an overnight culture in YPD were diluted in YPD to a final OD_600_ of 0.2. The cells were grown for 5 h at 30 °C with shaking. SNP and SHAM were added to the cultures to a final concentration of 1 mM and 0.5 mM respectively, or 25 µg/ml Calcofluor White (CFW) or 2 mM hydrogen peroxide as positive controls. Samples were taken to obtain cell pellets of 30 mg (fresh weight), after 1 h and after 18 h following drug addition.

To monitor Aox2 expression in response to NO, 1 mM SNP was added to log-phase wild-type or *aox2*Δ*aox1*Δ *C. albicans* cultures in YPD and samples were taken at 10, 20, 30 and 120 min to obtain cell pellets of 30 mg fresh weight. The cell pellets were snap frozen at -80 °C. Total protein was extracted by homogenisation at 4 °C with glass beads in the presence of 125 mM Tris-HCl, 2% SDS, 2 % glycerol, 0.14 M 2-mercaptoethanol, bromophenol blue buffer at 4 °C. Samples were run on a 5% stacking, 12.5% resolving SDS-polyacrylamide gel. Proteins were transferred to PVDF membrane using a semi-dry transfer system (Bio-Rad, Watford, UK). Phosphorylated Hog1 or Mkc1 were detected using p44/p42 (1:2000) or p38 (1:1000) monclonal antibodies (Cat. No. 4695 and 8690 respectively, Cell Signalling Technology, Netherlands) followed by a goat anti-rabbit-HRP secondary antibody (1:5000) (A0545, Sigma-Aldrich). A monoclonal antibody against *Sauromatum guttatum* Aox which recognises *C. albicans* Aox2 was used for Aox immunoblotting (1:100) (AS10 699, Agrisera, Sweden). Secondary binding of goat anti-mouse-HRP antibody (1:5000) (Sigma-Aldrich A9917) was detected by ECL and images captured using a Syngene GBox Chemi XX6 system. Blots were stripped using Restore™ Western Blot Stripping Buffer (Thermo Fisher Scientific, Cat. No. 21059) and re-probed for actin using an antibody raised against *S. cerevisiae* actin (a kind gift from Prof. John Cooper, University of Washington) at a 1:1000 dilution.

**Cell wall isolation and HPLC**

Cell wall material was isolated and acid hydrolysed in 2M trifluoroacetic acid from untreated cells and cells grown in the presence of 1 mM SNP and 0.5 mM SHAM for 18 h in triplicate (9). To determine the relative amounts of chitin, glucan and mannan, the hydrolysates were analysed by high-performance anion-exchange chromatography (10).

**Phagocytosis assay**

J774.1 murine macrophages (a kind gift from the lab of Prof. Carol Munro, Institute of Medical Sciences, University of Aberdeen) were maintained in DMEM with 10% FBS, 200 U/ml penicillin/streptomycin (respectively, Cat No. 10569010, 10082147, 15070063, Gibco, Thermo Fisher) at 37 °C, 5% CO_2_. Cells were counted and diluted in fresh medium to give 5 x 10^4^ cells in 0.3 ml then added to the wells of a 8 well µ-Slide (ibidi GmbH, Germany). The cells were then incubated overnight. *C. albicans* was grown in the presence of 1 mM SNP and 0.5 mM SHAM for 18 h. *C. albicans* cells were washed three times in PBS and counted, then diluted to 1.5 x 10^5^ cells in 0.3 ml in macrophage culture medium with 10 µg/ml Calcofluor White and vortexed briefly. This cell suspension was then added to macrophages (3:1 *C. albicans* to macrophage ratio). The cells were then co-incubated for 1 h and examined by microscopy. Uptake of *C. albicans* was manually assessed using ImageJ. The percentage uptake was determined as the number of internalised *C. albicans* relative to the total number of *C. albicans* cells. At least 200 *C. albicans* cells were counted for each experiment. Five independent experiments were performed.

**Hyphal induction**

*C. albicans* grown in the presence of 1mM SNP and 0.5 mM SHAM for 18 h were washed three times in PBS and the OD_600_was measured. Cells were added to DMEM + 10 % FBS with 10 µg/ml Calcofluor White to a final OD of 0.1 and added to a 8 well µ-Slide (0.3 ml per well). Following a 90 min incubation at 37 °C, 5% CO_2_, the cells were examined by microscopy using a DAPI filter. The percentage of hyphal cells was determined manually using ImageJ. A total of least 500 cells were counted for each experiment.

To determine the potential involvement of reactive oxygen species on hyphal formation, cells from an overnight culture in YPD were used to inoculate YPD, 0.1 % glucose, followed by incubation at 30 °C with shaking for 5 h. Five hundred microliters of culture was transferred to microcentrifuge tubes and N-Acetyl-L-cysteine (Sigma-Aldrich, A7250) was added to a final concentration of 5 mM. Cells were incubated at 30 °C for a further 30 min. Media was replaced with fresh YPD, 0.1 % glucose and cells were treated with 1 mM SNP, 0.5 mM SHAM or a combination for 1 h at 30 °C. Cells were washed three times in PBS and resuspended in YPD, 0.1 % glucose + 10% FBS and incubated at 37 °C for 90 min after which hyphal switching was evaluated by microscopy.

The effect of respiration inhibitors on reactive oxygen species generation was evaluated using a dihydroethidium assay. Cells were subjected to the same procedure as described for hyphal induction above, with or without N-acetyl cysteine pre-treatment, and were treated with 1 mM SNP, 0.5 mM SHAM or a combination for 1 h at 30 °C. Cells were then washed three times in PBS and resuspended in 250 μl of 2.5 μg/ml dihydroethidium (Thermo Fisher Scientific, D23107) in PBS, and incubated in the dark for 5 min. Relative fluorescence units (RFU) were determined using a BMG fluorescence reader and then normalized to OD_600._

**Zebrafish *in vivo* imaging**

Two-day old zebrafish of the *Tg(mpeg1:mCherryCAAX)sh378)* transgenic strain were used in this study. Zebrafish were maintained according to standard protocols. Adult fish were maintained on a 14:10 – hour light / dark cycle at 28 ^o^C in UK Home Office approved facilities in the Bateson Centre aquaria at the University of Sheffield. *C. albicans* grown in the presence of 1 mM SNP and 0.5 mM SHAM for 18 h were washed three times in PBS, counted and adjusted to give 500 colony forming units (CFU) in 1 nl and pelleted by centrifugation. Pellets were resuspended in 10% Polyvinylpyrrolidinone (PVP), 0.5% Phenol Red in PBS. The injection and imaging of zebrafish larvae was performed as described in (11) with the exception that the larvae were not immobilised in agar channels and were instead aligned manually in E3 containing 0.168 mg/mL tricaine in glass-bottomed, 96-well plates for imaging. Assessment of *C. albicans* uptake by macrophages was performed manually using NIS-Elements Viewer 4.20 (Nikon, Richmond, UK). Three independent experiments were performed using 10 larvae per condition.

**Electron microscopy**

*C. albicans* was grown in the presence of 1mM SNP and 0.5 mM SHAM for 18 h. A 1 ml sample of each culture was centrifuged and the cell pellet frozen under pressure using a Leica EM AFS2 automatic freeze substitution system and EM FSP freeze substitution processor (Leica Microsystems). Samples were dehydrated in anhydrous acetone containing 1% OsO_4_ for 10 h, sequentially warmed to -30°C over 8 h in acetone/OsO4, to 20 °C over 3 h in acetone and then embedded in increasing concentrations of Spurr’s (epoxy) resin over 24 h. Survey sections of 0.5 mm thickness were stained with toluidine blue to verify optimal cell density. All sections were cut with a 45° diamond knife (Diatome US, Hatfield, PA) using a Leica UC6 ultramicrotome. Ultrathin (100 nm) sections were adhered to 300 nm copper mesh grids for examination. Sections were stained with uranyl acetate and lead citrate at 20°C using a Leica AC20 EM automated staining machine. The freeze substitution and embedding, sectioning and staining was carried out by the Microscopy and Histology Facility of the Institute of Medical Sciences, University of Aberdeen. Examination of ultrathin sections was done with a JEOL 1400 Plus transmission microscope (JEOL UK Ltd.) operating at 80 kV and images were recorded using an AMT ActiveVu XR16M camera (Deben UK Ltd.). The AMT v602 camera software was used to visualise, edit and save the images. ImageJ was used to manually measure thicknesses of the inner (i) and outer (o) cell wall (as indicated in Figure 2B). Five measurements were taken along the cell wall for each cell, for a total of 25 cells per group.

**Lipid droplet staining**

The lipid droplet stain LD540 was a kind gift from C. Thiele (12). Wild-type *C. albicans* in log phase was treated with 1 mM SNP and 0.5 mM SHAM for 18 h. A sample of the culture was washed three times in PBS and stained with 0.1 µg/ml LD540 for 30 min at room temperature. The cells were washed three times in PBS and examined using an RFP filter with low-level brightfield illumination.

**Murine *C. albicans* systemic infection model**

The effect of SHAM+SNP pre-treatment of *C. albicans* was evaluated using a murine intravenous challenge assay. Pre-treatment of *C. albicans* was performed as follows: cells from an overnight culture in YPD were diluted in YPD to a final OD_600_ of 0.2. The cells were grown for 5 hours at 30 °C with shaking. SNP and SHAM were added to a final concentration of 1 mM and 0.5 mM respectively and cultures were grown for a further 18 h. BALB/c female mice (6-8 weeks old, Envigo UK) were randomly assigned into groups of 6, with group size determined by power analyses using data previously obtained using this infection model. Mice were acclimatized for 5 days prior to the experiment. Mice were weighed and tail-marked using a surgical marker pen to allow for identification. Food and water was provided *ad libitum*. *C. albicans* cells were washed twice with sterile saline and diluted in sterile saline to produce an inoculum of 4 x10^4^ CFU/g mouse body weight in 100 µl PBS. Inoculum level was confirmed by viable plate count on Sabouraud Dextrose agar. Mice were weighed and checked daily until day 3 post-infection when all mice were culled by cervical dislocation. The kidneys were removed aseptically for fungal burden determination, with kidneys weighed and homogenised in 0.5 ml sterile saline. Dilutions were plated on Sabouraud Dextrose agar and incubated overnight at 35 °C. Colonies were counted and expressed as colony forming units (CFU) per g of kidney. Change in weight was calculated as percentage weight change relative to starting weight. An outcome score was calculated based upon kidney burdens and weight loss at time of culling [13].

**Tissue processing and histology**

Mouse kidneys were cryosectioned using a Leica CM 1850 cryostat (Leica Biosystems, Newcastle Upon Tyne, UK). The distribution of fungal and immune cells was assessed with the periodic acid Schiff reagent and hematoxylin and eosin staining [14]. Optical images of 12 µm thick tissue cryosections were acquired with a NikonCoolScan, V ED slide scanner (Nikon, Kingston upon Thames, UK).

**Ethics statement**

Mouse experiments were carried out under licence PPL70/9027 awarded by the UK Home Office to Dr Donna MacCallum at the University of Aberdeen. All experiments conform to the UK Animals (Scientific Procedures) Act (ASPA) 1986 and EU Directive 2010/63/EU. Zebrafish work was performed following UK law: Animal (Scientific Procedures) Act 1986, under Project License PPL 40/3574 and P1A4A7A5E. Ethical approval was granted by the University of Sheffield Local Ethical Review Panel under project licenses 40/3574 and P1A4A7A5E.

**References**

1. Homann OR, Dea J, Noble SM, Johnson AD. 2009. A phenotypic profile of the *Candida albicans* regulatory network. PLoS Genet 5. https://doi.org/10.1371/journal.pgen.1000783.

2. Noble SM, French S, Kohn L a, Chen V, Johnson AD. 2010. Systematic screens of a *Candida albicans* homozygous deletion library decouple morphogenetic switching and pathogenicity. Nat Genet 42:590–598. https://doi.org/10.1038/ng.605.

3. Dunkel N, Liu TT, Barker KS, Homayouni R, Morschhäuser J, Rogers PD. 2008. A gain-of-function mutation in the transcription factor Upc2p causes upregulation of ergosterol biosynthesis genes and increased fluconazole resistance in a clinical *Candida albicans* isolate. Eukaryot Cell 7:1180–1190. https://doi.org/10.1128/EC.00103-08.

4. Noble SM, Johnson AD. 2005. Strains and strategies for large-scale gene deletion studies of the diploid human fungal pathogen *Candida albicans*. Eukaryot Cell 4:298–309. https://doi.org/10.1128/EC.4.2.298-309.2005.

5. Thompson JR, Register E, Curotto J, Kurtz M, Kelly R. 1998. An improved protocol for the preparation of yeast cells for transformation by electroporation. Yeast 14:565–571. https://doi.org/10.1002/(SICI)1097-0061(19980430)14:6<565::AID-YEA251>3.0.CO;2-B.

6. Afgan E, Baker D, van den Beek M, Blankenberg D, Bouvier D, Čech M, Chilton J, Clements D, Coraor N, Eberhard C, Grüning B, Guerler A, Hillman-Jackson J, Von Kuster G, Rasche E, Soranzo N, Turaga N, Taylor J, Nekrutenko A, Goecks J. 2016. The Galaxy platform for accessible, reproducible and collaborative biomedical analyses: 2016 update. Nucleic Acids Res 44:W3–W10. https://doi.org/10.1093/nar/gkw343.

7. Skrzypek MS, Binkley J, Binkley G, Miyasato SR, Simison M and SG. *Candida* Genome Database.

8. Schirmer RH, Adler H, Pickhardt M, Mandelkow E. 2011. “Lest we forget you - methylene blue...” Neurobiol Aging 32:2325.e7-2325.e16. https://doi.org/10.1016/j.neurobiolaging.2010.12.012.

9. Mora-Montes HM, Bates S, Netea MG, Díaz-Jiménez DF, López-Romero E, Zinker S, Ponce-Noyola P, Kullberg BJ, Brown AJP, Odds FC, Flores-Carreón A, Gow NAR. 2007. Endoplasmic reticulum α-glycosidases of *Candida albicans* are required for N glycosylation, cell wall integrity, and normal host-fungus interaction. Eukaryot Cell 6:2184–2193. https://doi.org/10.1128/EC.00350-07.

10. Lee KK, MacCallum DM, Jacobsen MD, Walker LA, Odds FC, Gow NAR, Munro CA. 2012. Elevated cell wall chitin in *Candida albicans* confers echinocandin resistance in vivo. Antimicrob Agents Chemother 56:208–217. https://doi.org/10.1128/AAC.00683-11.

11. Bojarczuk A, Miller KA, Hotham R, Lewis A, Ogryzko N V., Kamuyango AA, Frost H, Gibson RH, Stillman E, May RC, Renshaw SA, Johnston SA. 2016. *Cryptococcus neoformans* Intracellular Proliferation and Capsule Size Determines Early Macrophage Control of Infection. Sci Rep 6:21489. https://doi.org/10.1038/srep21489.

12. Spandl J, White DJ, Peychl J, Thiele C. 2009. Live cell multicolor imaging of lipid droplets with a new dye, LD540. Traffic 10:1579–1584. https://doi.org/10.1111/j.1600-0854.2009.00980.x.

13. MacCallum DM, Coste A, Ischer F, Jacobsen MD, Odds FC, Sanglard D. Genetic dissection of azole resistance mechanisms in *Candida albicans* and their validation in a mouse model of disseminated infection. Antimicrob Agents Chemother. 2010;54: 1476–1483. doi:10.1128/AAC.01645-09

14. McManus JF, Cason JE (1950) Carbohydrate histochemistry studied by acetylation techniques. J Exp Med 91: 651–654.
