# Supplementary material for: Inhibition of Classical and Alternative Modes of Respiration in Candida albicans Leads to Cell Wall Remodeling and Increased Macrophage Recognition
Source: mBio. 2019 Jan 29;10(1):e02535-18. doi: 10.1128/mBio.02535-18 (PMC6355986; doi:10.1128/mBio.02535-18)
Supplement: TABLE S1 [file mBio.02535-18-st001.pdf]

## Untreated vs SHAM: differentially expressed genes

| Assembly 21 Identifier | Systematic Name | Standard Name | Log[2]Fold Change |
|---|---|---|---|
| orf19.2061 | C2_00670C_A | | -3.39599 |
| CaalfMp08 | CM_00210W | COX1 | -3.1282 |
| CaalfMr17 | CM_00360W | RRNS | -3.11168 |
| orf19.2584 | CR_01860W_A | OPT9 | -3.0144 |
| CaalfMp06 | CM_00160C | ATP6 | -2.9761 |
| CaalfMr16 | CM_00010W | RRNL | -2.55001 |
| orf19.2158 | C6_04610C_A | NAG3 | -2.52398 |
| orf19.2060 | C2_00680C_A | SOD5 | -2.50865 |
| orf19.3902 | C5_04190W_A | MRV2 | -2.494 |
| orf19.2607 | CR_02060W_A | | -2.35466 |
| orf19.6899 | C7_01170C_A | | -2.32245 |
| orf19.4789 | C1_09300C_A | | -2.20674 |
| orf19.2948 | C1_02600W_A | SNO1 | -2.18285 |
| orf19.789 | C4_03940C_A | PYC2 | -2.15772 |
| orf19.2846 | CR_02880W_A | | -2.15462 |
| orf19.4737 | C1_08790W_A | TPO3 | -2.15285 |
| orf19.2602 | CR_02020C_A | OPT1 | -2.10906 |
| orf19.1449 | C2_01450C_A | | -1.91623 |
| orf19.5245 | C1_12190W_A | BUL4 | -1.83847 |
| orf19.386 | C1_08410C_A | SAM4 | -1.83333 |
| orf19.6249 | C1_06610C_A | HAK1 | -1.80297 |
| orf19.5805 | C2_02980C_A | DLD1 | -1.78042 |
| orf19.2284 | C2_07270W_A | | -1.74578 |
| orf19.797 | C2_04230W_A | BAT21 | -1.7348 |
| orf19.288 | C3_02950C_A | MET13 | -1.7279 |
| orf19.6500 | C7_02150C_A | ECM42 | -1.71408 |
| orf19.1789.1 | C4_05320W_A | LYS1 | -1.66507 |
| orf19.2970 | C1_02820W_A | LYS2 | -1.66236 |
| orf19.6222.1 | C1_06870C_A | | -1.65452 |
| orf19.4691 | C4_00980C_A | MRV1 | -1.65273 |
| orf19.5720 | C6_03540W_A | | -1.62911 |
| orf19.6277 | C1_06340W_A | | -1.61195 |
| orf19.1344 | C7_03310W_A | | -1.59969 |
| orf19.4506 | C2_04460W_A | LYS22 | -1.59524 |
| orf19.5097 | C1_08190C_A | CAT8 | -1.58662 |
| orf19.7637 | CR_10570C_A | YHB4 | -1.57012 |
| orf19.2839 | CR_02810W_A | CIRT4B | -1.54365 |
| orf19.7469 | CR_00620C_A | ARG1 | -1.5218 |
| orf19.1857 | CR_06770C_A | | -1.52004 |
| orf19.4788 | C1_09290C_A | ARG5,6 | -1.51423 |
| orf19.2525 | CR_01400W_A | LYS12 | -1.5099 |
| orf19.5505 | C7_03720C_A | HIS7 | -1.50456 |
| orf19.346 | C3_03480C_A | ALT1 | -1.49238 |
| orf19.2991 | C1_03000W_A | HOL1 | -1.45261 |
| orf19.6660 | C5_03510C_A | | -1.45162 |

| | | | |
|---|---|---|---|
| orf19.309 | C3_03120C_A | DAL5 | -1.43749 |
| orf19.3365 | C4_03380C_A | DAO2 | -1.43162 |
| orf19.2351 | C1_10700C_A | NIT3 | -1.42217 |
| orf19.1077 | C6_04210C_A | ATM1 | -1.40067 |
| orf19.1340 | C7_03350C_A | | -1.37031 |
| orf19.1596 | C4_03910W_A | FGR28 | -1.36801 |
| orf19.1832 | C1_10610W_A | FCY23 | -1.36477 |
| orf19.1585 | C2_02590W_A | ZRT2 | -1.34617 |
| orf19.3591 | C2_08800C_A | APE3 | -1.32689 |
| orf19.3221 | CR_01330W_A | CPA2 | -1.31772 |
| orf19.2283 | C2_07260C_A | DQD1 | -1.27902 |
| orf19.7436.1 | C3_06480C_A | ECM15 | -1.27657 |
| orf19.5504 | C7_03730C_A | | -1.27429 |
| orf19.6837 | C1_04450C_A | FMA1 | -1.27226 |
| orf19.4780 | C1_09220W_A | | -1.23865 |
| orf19.7095 | C7_00270W_A | | -1.18187 |
| orf19.5431 | C3_00360W_A | | 1.15577 |
| orf19.2933 | C1_02440C_A | | 1.25107 |
| orf19.6983 | C3_05450C_A | | 1.28433 |
| orf19.6178 | C3_07830W_A | FBP1 | 1.33015 |
| orf19.4438 | C1_07330W_A | RME1 | 1.35565 |
| orf19.4690 | C4_00990W_A | | 1.36177 |
| orf19.938 | C5_00510W_A | | 1.36406 |
| orf19.4377 | CR_03790C_A | KRE1 | 1.36985 |
| orf19.3395 | C6_01870C_A | | 1.37541 |
| orf19.4843 | C1_09780C_A | | 1.42726 |
| orf19.6992 | C3_05570W_A | QDR2 | 1.42759 |
| orf19.2823 | CR_02640W_A | RFG1 | 1.45092 |
| orf19.842 | C2_03790C_A | ASR3 | 1.45541 |
| orf19.2762 | C4_02410C_A | AHP1 | 1.48277 |
| orf19.1028 | C1_03810C_A | ELA1 | 1.51543 |
| orf19.5749 | C6_03750C_A | SBA1 | 1.51814 |
| orf19.6548 | C7_01760C_A | ISU1 | 1.55759 |
| orf19.2778 | C1_07660W_A | | 1.58285 |
| orf19.2244 | C2_06890C_A | | 1.58692 |
| orf19.1120 | C5_03750W_A | FAV2 | 1.59529 |
| orf19.4041 | C5_05430W_A | PEX4 | 1.612 |
| orf19.6077 | C1_00310W_A | | 1.61965 |
| orf19.2467 | C1_05840W_A | PRN1 | 1.62746 |
| orf19.6486 | C7_02270W_A | LDG3 | 1.64863 |
| orf19.3803 | C4_04770C_A | MNN22 | 1.65444 |
| orf19.5741 | C6_03700W_A | ALS1 | 1.671 |
| orf19.1149 | C1_11700C_A | MRF1 | 1.69104 |
| orf19.6487 | C7_02260W_A | | 1.69235 |
| orf19.4833 | C1_09690W_A | MLS1 | 1.70537 |
| RPR1 | C2_08055W_A | RPR1 | 1.73416 |
| orf19.6838 | C1_04460C_A | | 1.75729 |
| orf19.5437 | C3_00320W_A | RHR2 | 1.80083 |

| | | | |
|---|---|---|---|
| orf19.4527 | C1_01980W_A | HGT1 | 1.81485 |
| orf19.1979 | C5_00880C_A | GIT3 | 1.83369 |
| orf19.5447 | C3_00220W_A | HGT19 | 1.84128 |
| orf19.6993 | C3_05580C_A | GAP2 | 1.94224 |
| orf19.158 | C3_01150C_A | | 1.95118 |
| orf19.2496 | C3_00930W_A | ATO2 | 1.95119 |
| orf19.6398 | CR_08310C_A | | 1.96247 |
| orf19.2457 | C1_05920W_A | | 2.00038 |
| orf19.3337 | C1_01510W_A | | 2.01447 |
| orf19.1325 | C4_03540C_A | ECM38 | 2.05399 |
| orf19.2048 | C2_00760C_A | | 2.07704 |
| orf19.882 | C2_03390C_A | HSP78 | 2.07716 |
| orf19.5557 | C6_02830W_A | MNN4-4 | 2.09666 |
| orf19.1032 | C1_03770W_A | SKO1 | 2.10775 |
| orf19.5713 | C6_03480W_A | YMX6 | 2.12297 |
| orf19.3483 | C6_02420W_A | | 2.17019 |
| RDN58 | CR_08790W_A | RDN58 | 2.17985 |
| orf19.7503 | CR_00300W_A | CDA2 | 2.2002 |
| orf19.2344 | C1_10740C_A | ASR1 | 2.26868 |
| orf19.3127 | C4_06820C_A | CZF1 | 2.40683 |
| orf19.308 | C3_03110W_A | | 2.42025 |
| orf19.6840 | C1_04470C_A | | 2.42829 |
| orf19.6408 | CR_08420W_A | | 2.4604 |
| orf19.1599 | C2_09410W_A | | 2.4788 |
| orf19.125 | C6_01180C_A | EBP1 | 2.6396 |
| orf19.3433 | C6_01510W_A | OYE23 | 2.66457 |
| orf19.849 | C2_03710W_A | | 2.68066 |
| ITS2 | CR_08800W_A | ITS2 | 2.85343 |
| orf19.5663 | C4_00360C_A | RCH1 | 2.90348 |
| orf19.5759 | C6_03840C_A | SNQ2 | 2.90695 |
| orf19.251 | C3_02610C_A | GLX3 | 2.91869 |
| orf19.6824 | C3_06790W_A | TRY6 | 3.18418 |
| ITS1 | CR_08780W_A | ITS1 | 3.21801 |
| orf19.6391 | CR_08270W_A | | 3.2377 |
| orf19.5760 | C6_03850C_A | IHD1 | 3.59277 |
| orf19.6209 | C1_06980C_A | | 4.72892 |
| orf19.2350 | C1_10710C_A | | 5.07974 |
| orf19.7495 | CR_00380W_A | | 5.46286 |
| orf19.1438 | C4_03340C_A | | 9.93254 |
