# Supplementary material for: Inhibition of Classical and Alternative Modes of Respiration in Candida albicans Leads to Cell Wall Remodeling and Increased Macrophage Recognition
Source: mBio. 2019 Jan 29;10(1):e02535-18. doi: 10.1128/mBio.02535-18 (PMC6355986; doi:10.1128/mBio.02535-18)
Supplement: TABLE S2 [file mBio.02535-18-st002.pdf]

Supplementary Table S1

Table S1 – Strains used in this study

Strain	Parent	Genotype	Source
SC5314		Typed strain	
SN250	SN152	arg4/arg4 leu2/leu2::LEU2 his1/his1::HIS1 URA3/ura3::λimm434 IRO1/iro1:: λimm434	[35]
*aox2*Δ*aox1*Δ	SN87	his/his1 leu2/leu2 URA3/ura3::imm434 iro1::IRO1/iro1:: imm434 aox2-aox1::LEU2/aox2-aox1::HIS1	This work
*upc2*Δ	SC5314	upc2::FRT/upc2::FRT	[37]
*sko1*Δ	SN152	arg4/arg4 leu2/leu2 his1/his1 URA3/ura3::λimm434 IRO1/iro1Δ:: λimm434 sko1::LEU2/sko1::HIS1	[35]
*cek1*Δ	SN152	arg4/arg4 leu2/leu2 his1/his1 URA3/ura3::λimm434 IRO1/iro1:: λimm434 cek1::LEU2/cek1::HIS1	[42]
*ndh51*Δ	BWP17	arg4/arg4 his1/his1 ura3::λimm434/ura3::λimm434 ndh51::ARG4/ndh51::URA3	[7]
BWP17-mtGFP	BWP17	ura3::imm434/ura3::imm434 iro1/iro 1::imm434 his1::hisG/his1::hisG arg4 /arg4 RPS10/rps10::pACT1-mtGFP	This work
