# Supplementary material for: Inhibition of Classical and Alternative Modes of Respiration in Candida albicans Leads to Cell Wall Remodeling and Increased Macrophage Recognition
Source: mBio. 2019 Jan 29;10(1):e02535-18. doi: 10.1128/mBio.02535-18 (PMC6355986; doi:10.1128/mBio.02535-18)
Supplement: TABLE S3 [file mBio.02535-18-st003.pdf]

Untreated vs SNP+SHAM: differentially expressed genes

Assembly 21 Identifier	Systematic Name	Standard Name	Log[2]Fold Change
orf19.4653	C4_01340W_A		-4.68547
orf19.1774	C2_10070W_A		-4.56171
orf19.2633.1	CR_07740W_A		-4.2655
orf19.265	C3_02750W_A		-4.13214
orf19.6249	C1_06610C_A	HAK1	-3.64427
orf19.2060	C2_00680C_A	SOD5	-3.46733
orf19.2451	C1_05960W_A	PGA45	-3.38661
orf19.3895	C5_04130C_A	CHT2	-3.31134
orf19.4654	C4_01330W_A		-3.29706
orf19.3721	CR_02440W_A		-3.27372
orf19.183	C2_04810W_A	HIS3	-3.23322
orf19.4342	C5_03130W_A	SUT1	-3.21657
orf19.7514	CR_00200W_A	PCK1	-3.17769
orf19.4921.1	C1_12910W_A		-3.16504
orf19.2061	C2_00670C_A		-3.03546
CaalfMp09	CM_00310W	NAD2	-2.93663
orf19.5302	C4_04080C_A	PGA31	-2.83305
orf19.5850	CR_05520W_A	NOC2	-2.7821
orf19.2475	C1_05760C_A	PGA26	-2.75668
orf19.3115	C4_06940C_A		-2.74801
orf19.6886	C2_05750W_A		-2.73394
orf19.1030	C1_03790C_A		-2.69107
orf19.4793	C1_09330W_A		-2.641
orf19.6766	C3_07300W_A	NOP13	-2.62829
orf19.7098	C7_00240W_A		-2.62742
orf19.1815	CR_07080W_A		-2.62091
orf19.670.2	C1_11320C_A		-2.59541
orf19.6828	C3_06760W_A		-2.53144
orf19.6245	C1_06660W_A		-2.51066
orf19.715	CR_06510W_A		-2.50198
CaalfMr17	CM_00360W	RRNS	-2.48904
orf19.6906	C7_01250W_A	ASC1	-2.47569
orf19.1964	C5_01070C_A		-2.43863
orf19.7384	C3_06030W_A	NOG1	-2.42883
orf19.3547	C2_05160C_A		-2.4056
orf19.1306	C4_03720C_A		-2.40239
CaalfMp05	CM_00140C	ATP9	-2.39431
CaalfMp08	CM_00210W	COX1	-2.38899
orf19.716	CR_06500C_A		-2.37884
orf19.7107	C7_00160C_A		-2.37827
orf19.4447	C1_07200W_A	YMC1	-2.36105
orf19.8278	C1_11410C_A		-2.34706
orf19.6090	C1_00160C_A		-2.33503

orf19.4506	C2_04460W_A	LYS22	-2.31591
orf19.3015	C1_03230C_A	ARX1	-2.31511
orf19.9	CR_07120C_A		-2.30835
orf19.5905	C3_04510W_A		-2.30192
orf19.500	CR_04160C_A		-2.29883
orf19.4167	C4_00750C_A		-2.29725
orf19.809	C2_04120C_A		-2.29377
orf19.4093	C2_09320C_A	PES1	-2.26617
orf19.3352	C1_01620C_A		-2.26405
orf19.3540	C2_05090W_A	MAK5	-2.25646
CaalfMp01	CM_00030W	COX2	-2.2518
orf19.494	CR_04110W_A		-2.23745
orf19.2917	C4_06210C_A		-2.22612
orf19.7650	CR_10650W_A	LTV1	-2.2208
orf19.4813	C1_09490C_A	GUA1	-2.1975
orf19.1517	C2_02030W_A	ARO3	-2.19541
orf19.3978	C5_04910W_A		-2.19462
orf19.501	CR_04170W_A		-2.18001
orf19.3114	C4_06950W_A		-2.16591
orf19.1566	C2_02430W_A	UTP21	-2.15956
orf19.661	C1_11420W_A	KRR1	-2.15826
orf19.5049	C1_07790C_A		-2.15433
orf19.59	C1_05060W_A	REI1	-2.15243
orf19.7094	C7_00280W_A	HGT12	-2.14971
orf19.5010	C1_13730C_A	DIM1	-2.13727
orf19.2185	C2_07960C_A	NSA1	-2.13532
orf19.4191	C4_00510C_A	RLP24	-2.13233
orf19.3722	CR_02430C_A		-2.12507
orf19.4384.1	CR_03580C_A		-2.12462
orf19.5507	C7_03700C_A	ENP1	-2.1217
CaalfMp13	CM_00380W	NAD5	-2.1155
orf19.7154	C7_04190C_A	UTP18	-2.11542
orf19.3962	C5_04750C_A	HAS1	-2.10741
orf19.1388	C2_09660W_A		-2.09669
orf19.7062	C7_00570W_A	RPA135	-2.08536
orf19.6686	C7_03540C_A	ENP2	-2.07388
orf19.6902	C7_01200C_A	DBP7	-2.05789
orf19.4587	C4_02050W_A	HGH1	-2.05442
orf19.2712	C4_02830C_A	HCA4	-2.04749
orf19.787	C4_03950C_A		-2.02193
orf19.4760	C1_09040C_A		-2.01364
orf19.6417	CR_08490W_A	TSR1	-2.0123
orf19.2489	C1_05630C_A		-2.00779
orf19.5949	C3_04830C_A	FAS2	-2.0052
orf19.4640	C4_01450W_A	PWP1	-2.00023
CaalfMp06	CM_00160C	ATP6	-1.99763

orf19.3113	C4_06960W_A		-1.99566
orf19.1502	C2_01910W_A		-1.99419
orf19.4896	C1_10260C_A	RPA34	-1.99368
orf19.3810	C4_04720W_A		-1.99357
orf19.2564	CR_01710W_A		-1.99089
orf19.3676	C1_02190W_A	ABP140	-1.98487
orf19.7664	CR_10750C_A		-1.98444
orf19.7569	CR_09950C_A	SIK1	-1.97913
orf19.1642	C3_02040C_A		-1.97884
orf19.3902	C5_04190W_A	MRV2	-1.97759
orf19.6955	C3_03650W_A	HBR3	-1.97673
orf19.3669	C1_02120C_A	SHA3	-1.96868
orf19.7634	CR_10540C_A	MCD1	-1.95716
orf19.2362	CR_07030C_A		-1.94681
orf19.2633	CR_07730W_A	HGT14	-1.94566
orf19.7635	CR_10550W_A	DRS1	-1.93869
orf19.76	C6_04160C_A	SPB1	-1.93201
orf19.1609	C3_02350W_A		-1.9242
orf19.3037	C1_03370W_A		-1.92341
orf19.2043	C2_00810C_A		-1.91822
orf19.1633	C3_02130W_A	UTP4	-1.91764
orf19.5198	C1_04390C_A	NOP4	-1.9159
CaalfMr16	CM_00010W	RRNL	-1.91501
orf19.169	CR_02540W_A	CHO2	-1.91363
orf19.1409.1	C4_04390W_A		-1.91101
orf19.4669	C4_01200C_A	AAT22	-1.90979
orf19.4870	C1_10030W_A	DBP3	-1.90959
orf19.7593	CR_10170C_A		-1.90615
orf19.3291	C1_01030W_A	HMT1	-1.90414
orf19.124	C6_01170W_A	CIC1	-1.88204
orf19.4941	C1_13140C_A	TYE7	-1.86998
orf19.5299	C4_04100C_A	ECM1	-1.86663
orf19.3905	C5_04220W_A	MRV5	-1.86424
orf19.1578	C2_02540W_A		-1.86413
orf19.576	C5_00740W_A	CTF8	-1.86296
orf19.3778	C4_05010W_A		-1.86121
orf19.6648	CR_05660W_A	SDA1	-1.85702
orf19.6355	C1_12680W_A		-1.85345
orf19.7422	C3_06370C_A		-1.84924
orf19.3564	C2_05340C_A	RPC40	-1.84591
orf19.3287	CR_00680W_A	BUD22	-1.83866
orf19.4789	C1_09300C_A		-1.83747
orf19.4311	C5_02890W_A	YNK1	-1.83683
orf19.4273	C5_02590C_A		-1.82825
orf19.1902	C2_07340W_A	NOC4	-1.82356
orf19.2717	C4_02790C_A	SAS10	-1.81993

orf19.6785	C3_07150C_A	RPS12	-1.81868
orf19.3478	C6_02360W_A	NIP7	-1.81403
orf19.1747	C2_10310C_A	KIP2	-1.79746
orf19.1342	C7_03330C_A	SHM1	-1.79436
orf19.7050	C7_00690W_A	NOP15	-1.79404
orf19.5504	C7_03730C_A		-1.78663
orf19.2998	C1_03060C_A	TSR2	-1.77999
orf19.4698	C4_00960W_A	PTC8	-1.77904
orf19.2859	CR_02980C_A	SRP40	-1.77841
orf19.596.1	CR_08020C_A	NOP10	-1.77088
orf19.2934	C1_02450C_A		-1.76631
orf19.4492	C2_04570W_A		-1.76628
orf19.6236	C1_06740C_A	NOP6	-1.76231
orf19.7011	C7_01030C_A		-1.76201
orf19.2385	CR_03370C_A	KTI12	-1.7553
orf19.962	C5_00310C_A		-1.7546
orf19.3504	C6_02070C_A	RPL23A	-1.74041
orf19.5912	C3_04560W_A	MAK21	-1.73921
orf19.2948	C1_02600W_A	SNO1	-1.73403
orf19.1164	C1_11550W_A	GAR1	-1.72939
orf19.1539	C2_02220C_A		-1.72748
orf19.1833	C1_10620W_A		-1.72714
orf19.944	C5_00450C_A	IFG3	-1.72672
orf19.5207	C2_05840W_A		-1.72507
orf19.5216	C2_05960C_A		-1.72341
orf19.6002	C3_05240C_A	RPL8B	-1.72259
orf19.2250	C2_06960W_A	SPE3	-1.71634
orf19.526	CR_04360C_A	NHP2	-1.71219
orf19.6702	C7_03670W_A	DED81	-1.70826
orf19.7534	CR_00080W_A	MIS12	-1.70808
orf19.1047	C1_04130W_A	ERB1	-1.70443
orf19.693	CR_06580W_A		-1.70271
orf19.1966	C5_01060C_A	BUD23	-1.70126
orf19.5608	C6_03210C_A		-1.69553
orf19.3334	C1_01480C_A	RPS21	-1.69467
orf19.1789.1	C4_05320W_A	LYS1	-1.69459
orf19.6298	C5_01600C_A	SPB4	-1.69127
orf19.4177	C4_00650W_A	HIS5	-1.69122
orf19.4873	C1_10060C_A		-1.6891
orf19.6014	C1_00900W_A	RRS1	-1.68436
orf19.7197	C7_03850W_A		-1.67155
orf19.6317	CR_04740C_A	ADE6	-1.67116
orf19.2314	C1_11000C_A		-1.66871
orf19.7657	CR_10690W_A	POP3	-1.66809
orf19.4450.2	C1_07150W_A		-1.66681
orf19.1791	C4_05330C_A		-1.66428

orf19.1708	C3_01430W_A		-1.66404
orf19.2992	C1_03010W_A	RPP1A	-1.6578
orf19.665	C1_11380W_A	NEP1	-1.65609
orf19.7618	CR_10410C_A		-1.65498
orf19.1700	C3_01490W_A	RPS7A	-1.65166
orf19.3276	CR_00800C_A	PWP2	-1.64732
orf19.5232	C1_12310C_A	CSI2	-1.64377
orf19.6541	C7_01790C_A	RPL5	-1.6364
orf19.4746	C1_08870C_A	JIP5	-1.6354
orf19.6302	CR_04900C_A	PGA39	-1.63475
orf19.2709	C4_02870C_A	ZUO1	-1.63174
orf19.6418	CR_08500W_A		-1.62911
orf19.563	C2_09380W_A	RRP15	-1.6243
orf19.2546	CR_01590C_A	TRP2	-1.6195
orf19.6779	C3_07220C_A	PRO2	-1.61898
orf19.1415	C4_04320W_A	FRE10	-1.61476
orf19.4479	C1_04040C_A		-1.60943
orf19.3690.2	C1_02330C_A		-1.60844
orf19.5550	C6_02770W_A	MRT4	-1.60469
orf19.1886	C2_07450C_A	RCL1	-1.59823
orf19.1082	C6_04240W_A		-1.59754
orf19.1986	CR_07710W_A	ARO2	-1.59699
orf19.5267	C1_11990W_A		-1.59595
orf19.6265	C1_06460C_A	RPS22A	-1.59559
orf19.2959.1	C1_02700C_A		-1.5926
orf19.2594	CR_01950W_A		-1.59238
orf19.6234	C1_06760C_A		-1.59168
orf19.4401	CR_03570C_A	YVH1	-1.59022
orf19.603	CR_07950W_A	IMP4	-1.58465
orf19.6862	C4_05230C_A		-1.58261
orf19.4143	C5_01480W_A	FYV5	-1.5816
orf19.1344	C7_03310W_A		-1.57872
orf19.1646	C3_02020W_A		-1.57829
orf19.198	C2_09060C_A	ASN1	-1.57589
orf19.2654	C5_03250W_A	RMS1	-1.57532
orf19.270	C3_02790W_A		-1.57315
orf19.2770.1	C4_02320C_A	SOD1	-1.56875
orf19.3003.1	C1_03110W_A	RPL6	-1.55915
orf19.236	C3_02470C_A	RPL9B	-1.55864
orf19.5500	C2_06360C_A	MAK16	-1.55855
orf19.7489.3	CR_00430C_A		-1.55836
orf19.1915	C2_00070C_A	MPP10	-1.55719
orf19.6375	CR_08150W_A	RPS20	-1.55382
orf19.3393	C6_01890C_A		-1.55297
orf19.2309.2	C1_11060C_A	RPL2	-1.551
orf19.6975	C3_05370C_A	YST1	-1.54938

orf19.3553	C2_05230C_A	RPF2	-1.54193
orf19.5927	C3_04670C_A	RPS15	-1.54044
orf19.1124.2	C5_03700C_A		-1.5355
orf19.5341	C2_10620W_A	RPS4A	-1.53344
orf19.7332	CR_09370W_A	ELF1	-1.53094
orf19.4029	C5_05340W_A		-1.5255
orf19.2320	C1_10950C_A		-1.52362
orf19.5959	C3_04900W_A	NOP14	-1.52125
orf19.1701	C3_01480C_A	RKI1	-1.52099
orf19.1746	C2_10320C_A		-1.52025
orf19.2158	C6_04610C_A	NAG3	-1.51965
orf19.7160	C7_04140C_A		-1.51774
orf19.300	C3_03040W_A	AIP2	-1.51572
orf19.2059	C2_00690W_A		-1.51418
orf19.1449	C2_01450C_A		-1.50947
orf19.2386	CR_03360W_A		-1.50815
orf19.1091	C6_04330W_A	NOP8	-1.50357
orf19.386	C1_08410C_A	SAM4	-1.50064
orf19.6220.4	C1_06890C_A		-1.50028
orf19.6589	CR_09730C_A	SSF1	-1.49974
orf19.5364	C2_10810W_A		-1.49642
orf19.1199	C6_00370C_A	NOP5	-1.49616
orf19.2246	C2_06920C_A		-1.49191
orf19.2951	C1_02620C_A	HOM6	-1.48961
orf19.840	C2_03810C_A	RPL21A	-1.48728
orf19.6315	CR_04770C_A		-1.48621
orf19.493	CR_04100C_A	RPL15A	-1.48617
orf19.1624.1	C3_02200W_A		-1.48307
orf19.1090	C6_04320C_A		-1.48249
orf19.3794	C4_04850C_A	CSR1	-1.47911
orf19.2319	C1_10970W_A		-1.47702
orf19.4336	C5_03070W_A	RPS5	-1.47684
orf19.4349	C5_03170C_A		-1.47654
orf19.5498	C2_06340W_A	EFH1	-1.47549
orf19.7048.1	C7_00710W_A	RPS28B	-1.47526
orf19.2673	C4_03170W_A		-1.47454
orf19.2237.1	C2_06850W_A		-1.47272
orf19.6026	C1_00800C_A	ERG2	-1.47084
orf19.926	C5_00630C_A	EXO1	-1.46988
orf19.7599	CR_10240W_A	UTP5	-1.46559
orf19.2232	C2_06810C_A	RPL11	-1.45685
orf19.1274	C4_05870C_A		-1.45566
orf19.2504	C3_01000W_A	BMS1	-1.45473
orf19.5486.1	C2_06240W_A	SMD2	-1.45392
orf19.5466	C3_00090W_A	RPS24	-1.45271
orf19.5212	C2_05900W_A		-1.45042

orf19.1927	C5_01390C_A	SNM1	-1.44895
orf19.5505	C7_03720C_A	HIS7	-1.44791
orf19.1635	C3_02110W_A	RPL12	-1.44572
orf19.5243	C1_12210W_A	TRP3	-1.44492
orf19.4931.1	C1_13050W_A	RPL14	-1.44437
orf19.6882.1	C2_05710C_A		-1.44424
orf19.2196	C2_07860W_A		-1.44291
orf19.2795	C1_07500C_A	LHP1	-1.44258
orf19.5991	C3_05160C_A		-1.44057
orf19.5996.1	C3_05200W_A	RPS19A	-1.4399
orf19.813	C2_04080W_A		-1.43984
orf19.5885	C3_04380C_A		-1.43827
orf19.6085	C1_00180W_A	RPL16A	-1.43735
orf19.2237	C2_06840W_A	SPR1	-1.43636
orf19.6899	C7_01170C_A		-1.43467
orf19.477	CR_03950W_A		-1.43329
orf19.6916	C7_01350C_A		-1.43183
orf19.475	CR_03940W_A		-1.42949
orf19.3870	CR_06150C_A	ADE13	-1.42938
orf19.3159	C3_01200W_A	UTP20	-1.41945
orf19.7594	CR_10180W_A		-1.41759
orf19.2831	CR_02720C_A	RPC31	-1.41742
orf19.4909.1	C1_10390C_A	RPL42	-1.41444
orf19.4691	C4_00980C_A	MRV1	-1.41384
orf19.3788.1	C4_04900W_A	RPL30	-1.41195
orf19.4963	C1_13370W_A		-1.41058
orf19.7015	C7_00990W_A	RPP0	-1.40896
orf19.4933	C1_13070C_A	FAD3	-1.40781
orf19.541	CR_04500C_A		-1.40727
orf19.1064	C1_04290C_A	ACS2	-1.40627
orf19.677	C1_11240C_A	CHO1	-1.40566
orf19.269	C3_02780W_A	SES1	-1.40498
orf19.1446	C2_01410C_A	CLB2	-1.3987
orf19.7387	C3_06060W_A	ELP3	-1.3981
orf19.7552	CR_09800C_A		-1.39217
orf19.512	CR_04240C_A		-1.38982
orf19.2794	C1_07510W_A		-1.38981
orf19.3554	C2_05250C_A	AAT1	-1.38923
orf19.7018	C7_00960W_A	RPS18	-1.38769
orf19.5926	C3_04660C_A	ARG11	-1.387
orf19.2667	C4_03270W_A	RPF1	-1.38574
orf19.687.1	C6_01970C_A	RPL25	-1.38402
orf19.4563	C4_02260C_A		-1.38241
orf19.706	CR_06720W_A	NMD3	-1.38201
orf19.5263	C1_12030W_A	SER33	-1.38138
orf19.5293	C4_04160W_A		-1.38042

orf19.94	C6_00930C_A		-1.37713
orf19.1051	C1_04170C_A	HTA2	-1.37584
orf19.6022	C1_00840C_A	NRM1	-1.37212
orf19.3955	C5_04700C_A	MES1	-1.37084
orf19.2287	C2_07300C_A	RPA12	-1.36898
orf19.4268	C5_02550C_A	UTP13	-1.3683
orf19.7069	C7_00490C_A		-1.36414
orf19.3789	C4_04890C_A	RPL24A	-1.36368
orf19.172	CR_02520W_A	RPC19	-1.36288
orf19.2329.1	C1_10870W_A	RPS17B	-1.36261
orf19.4490	C2_04600C_A	RPL17B	-1.35873
orf19.3626	C2_08530C_A		-1.35692
orf19.2044	C2_00800C_A	PGA27	-1.35518
orf19.6477	C7_02340C_A		-1.3504
orf19.3300	C1_01110C_A	ZPR1	-1.34651
orf19.2179.2	C2_08040C_A	RPS10	-1.34645
orf19.3161	C5_02070C_A		-1.34392
orf19.255	C3_02640C_A	ZCF1	-1.34376
orf19.3415.1	C6_01700W_A	RPL32	-1.34376
orf19.6175	C3_07800C_A		-1.34101
orf19.3271	CR_00840C_A		-1.33962
orf19.6730	C3_07600W_A		-1.33535
orf19.1687	C3_01560W_A		-1.33499
orf19.4055	C5_05510C_A		-1.33214
orf19.95	C6_00940C_A		-1.3314
orf19.827.1	C2_03960W_A	RPL39	-1.32845
orf19.7188	C7_03920C_A	RPP1B	-1.32446
orf19.2846	CR_02880W_A		-1.32431
orf19.4193.1	C6_00650C_A	RPS13	-1.32406
orf19.5904	C3_04500C_A	RPL19A	-1.32144
orf19.2832	CR_02740W_A	INN1	-1.32016
orf19.4190	C4_00520W_A	PAM18	-1.32003
orf19.2042	C2_00820W_A		-1.3194
orf19.7161	C7_04130C_A	SUI3	-1.31872
orf19.2723	C4_02750W_A	HIT1	-1.31537
orf19.3756	C1_12600C_A	CHR1	-1.31369
orf19.6903	C7_01210C_A		-1.31272
orf19.3942.1	C5_04590C_A	RPL43A	-1.31219
orf19.5225.2	C1_12390C_A	RPL27A	-1.31072
orf19.2864.1	CR_03030C_A	RPL28	-1.30999
orf19.5976	C3_05060W_A		-1.30974
orf19.6938	C3_03790W_A	MEU1	-1.30746
orf19.2994	C1_03020C_A	RPL13	-1.30633
orf19.3341	C1_01530C_A		-1.30395
orf19.7564	CR_09900C_A	DPB2	-1.30353
orf19.1305	C4_03730C_A		-1.30242

orf19.5987	C3_05140C_A		-1.30138
orf19.5567	C6_02910W_A	POP4	-1.30063
orf19.1832	C1_10610W_A	FCY23	-1.3001
orf19.2306	C1_11100W_A		-1.29914
orf19.6265.1	C1_06450C_A	RPS14B	-1.2949
orf19.6415.1	CR_08480C_A		-1.29314
orf19.5779	C2_03160C_A	RNR1	-1.29231
orf19.3477	C6_02350C_A		-1.29144
orf19.1202	C6_00400C_A		-1.29109
orf19.2090	C2_00410C_A		-1.2887
orf19.4835	C1_09710C_A		-1.28477
orf19.967	C5_00280C_A		-1.28329
orf19.1631	C3_02150C_A	ERG6	-1.28122
orf19.3244	CR_01100C_A		-1.27293
orf19.5746	C6_03720W_A	ALA1	-1.27226
orf19.3990	C5_04990W_A		-1.27051
orf19.5061	C1_07890C_A	ADE5,7	-1.26989
orf19.4171	C4_00710W_A		-1.26951
orf19.1507	C2_01950C_A	AMN1	-1.26886
orf19.5188	C7_02770W_A	CHS1	-1.26752
orf19.6316.4	CR_04750W_A		-1.2657
orf19.6328	C6_00090W_A		-1.26416
orf19.930	C5_00590W_A	PET9	-1.26395
orf19.4931	C1_13030C_A		-1.26357
orf19.2830	CR_02710W_A	RRP9	-1.2632
orf19.4987	C1_13550C_A	NUP49	-1.26085
orf19.7466	CR_00640W_A	ACC1	-1.26038
orf19.6679	C5_03670C_A		-1.25858
orf19.5832	C2_02740C_A	HPT1	-1.25748
orf19.5351	C2_10710W_A	TIF11	-1.25501
orf19.5750	C6_03760C_A	SHM2	-1.25017
orf19.3475	C6_02330W_A		-1.24965
orf19.979	C5_00190C_A	FAS1	-1.2483
orf19.568	C5_00700C_A	SPE2	-1.24773
orf19.3812	C4_04700W_A	SSZ1	-1.24511
orf19.4399	CR_03550W_A	NCS2	-1.24268
orf19.5430	C3_00370C_A	BUD21	-1.24191
orf19.518	CR_04300W_A		-1.23905
orf19.2711.1	C4_02840C_A	MED20	-1.23847
orf19.667.1	C1_11360W_A	RPL37B	-1.23769
orf19.4651	C4_01360W_A	PGA53	-1.23678
orf19.1697	C3_01520C_A		-1.23672
orf19.3354	C1_01640W_A	RPS42	-1.23515
orf19.3249	CR_01060W_A	LAG1	-1.23327
orf19.2183	C2_08000C_A	KRE30	-1.23218
orf19.6652	CR_05630W_A	DBP8	-1.23082

orf19.1559	C2_02370C_A	HOM2	-1.23072
orf19.6710	C3_07750W_A	UTP9	-1.23001
orf19.1839	C1_10670C_A	RPA190	-1.22975
orf19.3724	CR_02420W_A		-1.22919
orf19.2263	C2_07080C_A		-1.2289
orf19.4324	C5_02990W_A		-1.22862
orf19.118	C6_01110W_A	FAD2	-1.22852
orf19.3798	C4_04810C_A		-1.22834
orf19.4660	C4_01270W_A	RPS6A	-1.22817
orf19.2885	C4_06490C_A	PRI2	-1.22802
orf19.1318	C4_03580W_A		-1.22557
orf19.1448	C2_01430W_A	APT1	-1.22249
orf19.6776	C3_07250W_A	GCD2	-1.22166
orf19.4035	C5_05390C_A	PGA4	-1.22151
orf19.5873	C3_04300C_A	POL1	-1.22017
orf19.2478.1	C1_05720W_A		-1.21891
orf19.6264.4	C1_06470W_A		-1.21835
orf19.3609	C2_08670C_A	UTP15	-1.21833
orf19.4632	C4_01520C_A	RPL20B	-1.21744
orf19.1404	C2_09500W_A		-1.21709
orf19.2796	C1_07490C_A		-1.21652
orf19.1352	C2_08310W_A	TIM22	-1.21509
orf19.4635	C4_01490W_A	NIP1	-1.21449
orf19.102	C6_01000C_A		-1.21436
orf19.6461	C7_02480W_A		-1.21426
orf19.1533	C2_02170W_A		-1.21421
orf19.8	CR_07110C_A		-1.21362
orf19.4161	C4_00800W_A		-1.20987
orf19.2994.1	C1_03030W_A	RPS16A	-1.20942
orf19.5685	C5_00110C_A	THS1	-1.2091
orf19.6873	C2_05610C_A	RPS8A	-1.20879
orf19.1470	C2_01610C_A	RPS26A	-1.2074
orf19.1626	C3_02180C_A		-1.20655
orf19.5083	C1_08100W_A	DRG1	-1.20619
orf19.1250	C4_05650W_A		-1.20552
orf19.6403.1	CR_08360C_A	RPP2A	-1.20372
orf19.5220	C1_12440W_A		-1.20275
orf19.3867	CR_06120W_A	RPL7	-1.20004
orf19.3938	C5_04530W_A		-1.19859
orf19.3710	CR_07810W_A	YHB5	-1.1971
orf19.2960	C1_02710W_A	FRS2	-1.19673
orf19.3667	C1_02100W_A		-1.19639
orf19.4060	C1_05110C_A	ARO4	-1.19538
orf19.6074	C1_00340W_A	HBR1	-1.19412
orf19.7546	CR_09740W_A		-1.19313
orf19.4212	C6_00470C_A	FET99	-1.19291

orf19.4718	C4_06110C_A	TRP5	-1.19242
orf19.2711	C4_02850W_A		-1.19118
orf19.3034	C1_03350C_A	RLI1	-1.18985
orf19.2256	C2_07000W_A		-1.18894
orf19.3268	CR_00860C_A	TMA19	-1.18851
orf19.3998	C5_05060C_A		-1.1885
orf19.5066	C1_07950C_A		-1.1885
orf19.4885	C1_10160W_A	MIR1	-1.18795
orf19.3325.3	C1_01370C_A	RPS21B	-1.18727
orf19.5791	C2_03080W_A	IDH2	-1.18718
orf19.1575	C2_02510W_A	PRS1	-1.18659
orf19.339	C3_03420C_A	NDE1	-1.18643
orf19.5484	C2_06210C_A	SER1	-1.18606
orf19.5783	C2_03130W_A		-1.18582
orf19.2227	C2_06760C_A		-1.18557
orf19.6025	C1_00810W_A		-1.18512
orf19.4815	C1_09510W_A	YTM1	-1.18371
orf19.3539	C2_05080C_A		-1.18258
orf19.389	C1_08440C_A		-1.18048
orf19.2688	C4_03030C_A	NAN1	-1.17956
orf19.6297	C5_01610W_A		-1.17832
orf19.2018	C2_01060C_A		-1.17734
orf19.2111.2	C2_00210W_A	RPL38	-1.17727
orf19.6917	C7_01360C_A		-1.17612
orf19.3002	C1_03090W_A	RPS1	-1.17529
orf19.1304	C4_03740W_A		-1.17527
orf19.7517	CR_00180C_A	CHT1	-1.17465
orf19.6511	C7_02060W_A	LIG1	-1.17405
orf19.3977	C5_04900C_A		-1.17368
orf19.4026	C5_05320C_A	HIS1	-1.17334
orf19.5254	C1_12110C_A		-1.16847
orf19.4796	C1_09350W_A		-1.16819
orf19.6994	C3_05590C_A	BAT22	-1.16736
orf19.4365	CR_03660C_A		-1.16367
orf19.7127	C7_04360C_A	TLO16	-1.16349
orf19.5964.2	C3_04960W_A	RPL35	-1.16281
orf19.850	C2_03700W_A		-1.16232
orf19.2378	CR_03430W_A		-1.15983
orf19.5106	C1_08290C_A	DIP2	-1.15983
orf19.5814.1	C2_02910W_A		-1.15957
orf19.1109	C5_03850W_A		-1.15821
orf19.4884	C1_10150W_A	WOR1	-1.1577
orf19.2560	CR_01690C_A	CDC60	-1.15524
orf19.3331	C1_01450W_A	ABC1	-1.15451
orf19.3687	C1_02300W_A		-1.15415
orf19.6286.2	CR_07630C_A	RPS27	-1.15398

orf19.2222	C2_08270C_A		-1.14866
orf19.7645	CR_10620C_A		-1.14693
orf19.6663	C5_03540C_A	RPS25B	-1.14644
orf19.5052	C1_07820W_A		-1.14366
orf19.7291	CR_08940W_A		-1.14189
orf19.3206	C5_01690C_A	CCT7	-1.14114
orf19.3630	C2_08480W_A	RRP8	-1.1367
orf19.5838	C2_02690W_A	SER2	-1.13458
orf19.2422	CR_03060W_A	ARC1	-1.13455
orf19.6901	C7_01190W_A		-1.13403
orf19.117	C6_01100W_A	SEC9	-1.13073
orf19.4704	C4_00890W_A	ARO1	-1.12665
orf19.4022	C5_05280C_A	SDH4	-1.1266
orf19.1847	CR_06860C_A	ARO10	-1.12365
orf19.1390	C2_09640W_A	PMI1	-1.1233
orf19.4684.2	C4_01050C_A	RPL40B	-1.12283
orf19.2487	C1_05650W_A		-1.12225
orf19.773	C1_04710C_A		-1.12114
orf19.3836	C4_04500C_A		-1.12055
orf19.58	C1_05050C_A	RRP6	-1.1204
orf19.1295	C2_06640C_A	VAS1	-1.12002
orf19.2575	CR_01780W_A		-1.11797
orf19.5235	C1_12280C_A		-1.11764
orf19.2970	C1_02820W_A	LYS2	-1.11602
orf19.1613	C3_02320W_A	ILV2	-1.1146
orf19.7382	C3_06010W_A	CAM1	-1.11371
orf19.4517	C2_04370W_A		-1.11233
orf19.6337	C6_00030W_A	TLO13	-1.11104
orf19.1117	C5_03770C_A		-1.11102
orf19.5137.1	C7_03180C_A	HHO1	-1.11054
orf19.1335	C7_03400C_A		-1.10956
orf19.1029	C1_03800W_A	RPP1	-1.10741
orf19.3099	C4_07090C_A	TRP4	-1.10645
orf19.3087	C4_07180W_A	UBI3	-1.10565
orf19.1340	C7_03350C_A		-1.10515
orf19.3110	C4_06990W_A		-1.10473
orf19.3960	C5_04740C_A	HYS2	-1.10436
orf19.2527	CR_01410C_A		-1.10374
orf19.5732	C6_03640W_A	NOG2	-1.09827
orf19.7112	C7_00100W_A	FRP2	-1.09815
orf19.1052	C1_04180W_A		-1.0966
orf19.6326	C6_00110C_A		-1.0964
orf19.4801	C1_09390W_A		-1.09577
orf19.2310.1	C1_11040W_A	RPL29	-1.09441
orf19.4283	C5_02660C_A		-1.09419
orf19.3066	C1_03680W_A	ENG1	-1.09097

orf19.3304	C1_01160C_A		-1.0888
orf19.4634	C4_01500W_A		-1.08729
orf19.4244	C5_02360C_A		-1.08348
orf19.7063	C7_00560C_A	THG1	-1.08275
orf19.7624	CR_10470C_A		-1.08229
orf19.4811	C1_09470C_A		-1.08202
orf19.1367.1	C2_09830C_A		-1.08148
orf19.3518	CR_05480W_A		-1.081
orf19.596.2	CR_08010W_A	DAD4	-1.08039
orf19.3435	C6_01490C_A		-1.07955
orf19.4630	C4_01550C_A	CPA1	-1.0793
orf19.6378	CR_08180C_A	TRM9	-1.07552
orf19.2017	C2_01070W_A		-1.07514
orf19.7488	CR_00460C_A		-1.07409
orf19.2479	C1_05710C_A	UGA4	-1.0736
orf19.4101	C2_06160W_A		-1.07309
orf19.6749	C3_07410C_A	KRS1	-1.07227
orf19.6213	C1_06960W_A	SUI2	-1.07219
orf19.3541	C2_05100C_A	ERF1	-1.07105
orf19.4932	C1_13060C_A		-1.07001
orf19.3691	C1_02350W_A	TIM21	-1.06804
orf19.3463	C6_02230W_A		-1.067
orf19.7359	C3_05780C_A	CRZ1	-1.067
orf19.6530	C7_01910C_A		-1.06356
orf19.5100	C1_08210C_A	MLT1	-1.06345
orf19.2209	C2_07730W_A	YVC1	-1.0606
orf19.3396	C6_01860C_A	HCH1	-1.06036
orf19.28	C2_06520C_A		-1.06023
orf19.2529.1	CR_01440C_A		-1.05927
orf19.6696	C7_03630C_A	TIM9	-1.05429
orf19.2715	C4_02800W_A	RPC53	-1.05272
orf19.4983	C1_13520C_A	MCU1	-1.05223
orf19.597	CR_08000C_A		-1.05219
orf19.2573	CR_01760C_A	FRS1	-1.05098
orf19.4400	CR_03560W_A		-1.05093
orf19.6062.3	C1_00450C_A		-1.05045
orf19.81	C2_06660W_A		-1.04754
orf19.5928	C3_04680W_A	RPP2B	-1.04671
orf19.5639	C4_00140C_A	HIS4	-1.04635
orf19.7290	CR_08930C_A		-1.04629
orf19.1061	C1_04260W_A	HHT21	-1.04412
orf19.6869	C2_05570C_A		-1.04408
orf19.4826	C1_09630W_A	IDH1	-1.04296
orf19.649	CR_05030W_A		-1.04282
orf19.3138	C4_06720W_A	NOP1	-1.0428
orf19.1830	C1_10580C_A		-1.04224

orf19.1108	C5_03860W_A	HAM1	-1.04184
orf19.2351	C1_10700C_A	NIT3	-1.04139
orf19.7238	C1_14280C_A	NPL3	-1.04133
orf19.4666	C4_01230C_A		-1.04076
orf19.4375.1	CR_03770C_A	RPS30	-1.03892
orf19.5488	C2_06260W_A		-1.03822
orf19.3974	C5_04880C_A	PUT2	-1.03815
orf19.4600	C4_01930C_A		-1.03689
orf19.3265	CR_00900W_A	TRM1	-1.03561
orf19.2018.2	C2_01050W_A		-1.03541
orf19.6781	C3_07200C_A	ZFU2	-1.03488
orf19.2284	C2_07270W_A		-1.03429
orf19.5133	C7_03220C_A	ZCF29	-1.03276
orf19.527	CR_04370W_A		-1.03246
orf19.7057	C7_00620W_A	GUS1	-1.03228
orf19.2500	C3_00960W_A		-1.03177
orf19.107	C6_01040C_A		-1.0305
orf19.405	C1_08580C_A	VCX1	-1.03039
orf19.5771	C6_03940C_A	PBP2	-1.02891
orf19.1735	CR_04710W_A		-1.02836
orf19.2462	C1_05870W_A	PRN3	-1.02469
orf19.5789	C2_03090C_A	ADE8	-1.02435
orf19.334	C3_03370C_A		-1.0233
orf19.639	CR_05160C_A		-1.02293
orf19.4188	C4_00540C_A	NMD5	-1.02281
orf19.6000	C3_05220W_A	CDR1	-1.02222
orf19.18	C2_06390C_A	IMH3	-1.02113
orf19.1716	C3_01350C_A	URA3	-1.02101
orf19.2581	CR_01810C_A		-1.01879
orf19.2091	C2_00400C_A		-1.01382
orf19.5684	C5_00130C_A		-1.0124
orf19.1420	C4_04280C_A		-1.01206
orf19.5211	C2_05890C_A	IDP1	-1.01132
orf19.2066	C2_00620C_A		-1.00839
orf19.6435	CR_08610W_A		-1.00836
orf19.252	C3_02620C_A		-1.00686
orf19.5597	C6_03120W_A	POL5	-1.0067
orf19.2416.1	CR_03090C_A	MLC1	-1.00666
orf19.2847	CR_02890C_A		-1.00641
orf19.1361	C2_09900C_A	TIM23	-1.00578
orf19.1611	C3_02330C_A		-1.00572
orf19.5566	C6_02900C_A		-1.00504
orf19.3358	C1_01690C_A	LSC1	-1.00131
orf19.7375	C3_05940C_A		-0.996071
orf19.154	C2_04700C_A		-0.994412
orf19.5824	C2_02820C_A		-0.993787

orf19.4451	C1_07140C_A	RIA1	-0.993694
orf19.2775	C4_02280W_A	IDI1	-0.990959
orf19.4205.1	C6_00530C_A		-0.990433
orf19.6066	C1_00410C_A		-0.990172
orf19.4005	C5_05130C_A		-0.989682
orf19.6630	CR_05800C_A		-0.989546
orf19.2488	C1_05640C_A	FAL1	-0.989008
orf19.7218	C1_14120C_A	RBE1	-0.987587
orf19.2115	C2_00170C_A		-0.986928
orf19.4591	C4_02020W_A	CAT2	-0.98463
orf19.423	C1_05420W_A		-0.98303
orf19.3010	C1_03180W_A		-0.98082
orf19.3406	C6_01780C_A		-0.980311
orf19.168	CR_02550C_A		-0.980239
orf19.5012	C1_13750C_A		-0.979126
orf19.3001	C1_03080C_A	TEM1	-0.978369
orf19.4582	C4_02090C_A		-0.978047
orf19.3042	C1_03410W_A		-0.977805
orf19.1565	C2_02420C_A		-0.977798
orf19.498	CR_04140W_A		-0.97568
orf19.7460	CR_00670C_A		-0.974813
orf19.6010	C1_00950C_A	CDC5	-0.97165
orf19.6259	C1_06540C_A		-0.966347
orf19.7504	CR_00290W_A		-0.966335
orf19.2266	C2_07110C_A		-0.965348
orf19.3455	C6_02150C_A		-0.965249
orf19.6925	C3_03900C_A	HTB1	-0.963959
orf19.2687.1	C4_03040W_A		-0.961968
orf19.7473	CR_00600C_A		-0.957694
orf19.4223	C5_02170C_A	GCD11	-0.956553
orf19.657	C1_11450C_A	SAM2	-0.956502
orf19.3087.1	C4_07170C_A		-0.955096
orf19.5271	C1_11940C_A		-0.954947
orf19.5906	C3_04520C_A	ADE2	-0.95387
orf19.3221	CR_01330W_A	CPA2	-0.953447
orf19.2309	C1_11070W_A	PET127	-0.953437
orf19.2299	C1_11160C_A		-0.953324
orf19.4176	C4_00660W_A		-0.95195
orf19.558	CR_05220C_A	GUT1	-0.950314
orf19.7236	C1_14260C_A	TIF35	-0.94992
orf19.2829	CR_02700W_A		-0.946569
orf19.4172	C4_00700C_A		-0.944127
orf19.6239	C1_06710W_A		-0.942478
orf19.989	C1_10470W_A		-0.940528
orf19.5684.1	C5_00120W_A		-0.939372
orf19.287	C3_02940C_A	NUO2	-0.938448

orf19.6374	CR_08130W_A		-0.938287
orf19.6632	CR_05790C_A	ACO2	-0.937736
orf19.6718	C3_07690C_A		-0.937562
orf19.3572.3	C2_05410W_A		-0.937338
orf19.7658	CR_10700W_A	RFC4	-0.937244
orf19.3986	C5_04970C_A	PPR1	-0.937047
orf19.7035	C7_00820W_A	RFC2	-0.936973
orf19.234	C3_02460C_A	PHA2	-0.93537
orf19.24	C2_06470W_A	RTA2	-0.935343
orf19.3471	C6_02300C_A		-0.934267
orf19.2746	C4_02550C_A	EMP70	-0.933939
orf19.150	C2_04680W_A	TIM17	-0.931939
orf19.1258	C4_05730W_A		-0.931797
orf19.1673	C3_01710C_A	PPT1	-0.930639
orf19.7064	C7_00550C_A	GLN4	-0.926845
orf19.4962	C1_13360C_A	RMP1	-0.926522
orf19.5038	C4_03830W_A		-0.924772
orf19.658	C1_11440C_A	GIN1	-0.924722
orf19.5407	C3_00560C_A	SOF1	-0.923702
orf19.319	C3_03200C_A		-0.922497
orf19.4537	C1_01860W_A		-0.922248
orf19.7386	C3_06050C_A		-0.921849
orf19.4805	C1_09430W_A		-0.921114
orf19.2075	C2_00520W_A	DFG5	-0.919035
orf19.520	CR_04330W_A		-0.918907
orf19.955	C5_00340W_A		-0.918877
orf19.1760	C2_10210C_A	RAS1	-0.91763
orf19.3311	C1_01240W_A	IFD3	-0.916927
orf19.3838	C4_04480C_A	EFB1	-0.916236
orf19.1618	C3_02280C_A	GFA1	-0.916112
orf19.825	C2_03990W_A	GCD7	-0.914943
orf19.4341	C5_03120W_A		-0.914751
orf19.3956	C5_04710W_A		-0.914218
orf19.1968.1	C5_01020C_A		-0.913353
orf19.3061.1	C1_03620C_A		-0.911559
orf19.1115	C5_03790W_A	GUK1	-0.911508
orf19.6230	C1_06800W_A		-0.911403
orf19.5985	C3_05120C_A		-0.910516
orf19.6136	CR_07320C_A		-0.907974
orf19.4657	C4_01300W_A		-0.907772
orf19.2819	CR_02600W_A		-0.907477
orf19.6500	C7_02150C_A	ECM42	-0.90656
orf19.6685	C7_03530C_A	ISY1	-0.906045
orf19.5467	C3_00060W_A	TLO7	-0.905706
orf19.22	C2_06440C_A		-0.905015
orf19.2499	C3_00950C_A		-0.904423

orf19.1260	C4_05750C_A	LEA1	-0.904349
orf19.6264.3	C1_06480C_A		-0.903972
orf19.4363	CR_03650W_A	SGD1	-0.903447
orf19.3757	C1_12590W_A	ATP20	-0.90324
orf19.2128	C6_04450W_A		-0.90189
orf19.3755	C1_12610W_A		-0.901028
orf19.514	CR_04260W_A	SNP3	-0.899575
orf19.6612	CR_09510C_A		-0.899014
orf19.7366	C3_05860C_A		-0.89673
orf19.2461	C1_05880W_A	PRN4	-0.895244
orf19.2167	C2_08180C_A		-0.895204
orf19.1317	C4_03590C_A	OSH3	-0.894676
orf19.1375	C2_09750W_A	LEU42	-0.894189
orf19.6113	C1_00030C_A		-0.892545
orf19.1515	C2_02010C_A	CHT4	-0.892362
orf19.6900	C7_01180W_A	MDM12	-0.892312
orf19.5279	C1_11880W_A		-0.892032
orf19.4825	C1_09620C_A		-0.888743
orf19.4160	C4_00810C_A		-0.888024
orf19.3941	C5_04570C_A	URA7	-0.887621
orf19.755	C1_04880C_A	MRPL37	-0.887543
orf19.6524	C7_01970C_A	TOM40	-0.887391
orf19.2192	C2_07900W_A	GDH2	-0.887097
orf19.3954.1	C5_04690C_A		-0.886398
orf19.6853	C1_04600C_A		-0.885939
orf19.5455	C3_00170C_A		-0.884002
orf19.1303	C4_03750C_A		-0.883915
orf19.515	CR_04270C_A		-0.881694
orf19.5081	C1_08090C_A	FUN12	-0.8809
orf19.1255	C4_05700W_A	ZCF5	-0.877049
orf19.1264	C4_05780C_A	CFL2	-0.876456
orf19.2541	CR_01550C_A		-0.876117
orf19.6904	C7_01220W_A	GCN3	-0.8759
orf19.2283	C2_07260C_A	DQD1	-0.875716
orf19.4266	C5_02540C_A	SPR28	-0.875054
orf19.6283	CR_07670W_A		-0.87486
orf19.6156	C3_00840C_A		-0.873664
orf19.5660.1	C4_00330C_A		-0.873316
orf19.6112	C1_00040W_A	CTA2	-0.871433
orf19.6060	C1_00480C_A	GCN20	-0.870216
orf19.3704	CR_07750C_A		-0.870037
orf19.754	C1_04890W_A	YBN5	-0.869511
orf19.3423	C6_01630W_A	TIF3	-0.868512
orf19.3530	C2_04980C_A	CKA2	-0.867151
orf19.4028	C5_05330C_A	RER2	-0.867147
orf19.3732	CR_02370W_A	ERG25	-0.866834

orf19.3533	C2_05020W_A		-0.864948
orf19.3759	C1_12570C_A		-0.863903
orf19.714	CR_06520C_A		-0.863702
orf19.5970	C3_05010C_A		-0.863685
orf19.1394	C2_09600C_A		-0.863297
orf19.3945	C5_04610W_A		-0.862778
orf19.7255	C1_14390W_A	RPC10	-0.86154
orf19.3675	C1_02180W_A	GAL7	-0.861319
orf19.839	C2_03830W_A		-0.860939
orf19.2446	C1_05990C_A		-0.860396
orf19.7626	CR_10490W_A	EIF4E	-0.860198
orf19.6958	C3_03630W_A	ECM18	-0.859336
orf19.4340.1	C5_03110C_A		-0.859323
orf19.3549	C2_05190W_A	CDC21	-0.859085
orf19.6662	C5_03530C_A		-0.858901
orf19.1707	C3_01440C_A		-0.8586
orf19.4516	C2_04380C_A		-0.858274
orf19.3255	CR_01010W_A	TEN1	-0.857546
orf19.5574	C6_02960W_A		-0.857285
orf19.5767	C6_03910C_A		-0.857183
orf19.6788	C3_07120W_A		-0.855159
orf19.5168	C7_02930C_A		-0.853401
orf19.5564	C6_02880W_A		-0.853
orf19.3327	C1_01380C_A	TRM2	-0.852211
orf19.3175	C5_01960C_A		-0.850781
orf19.4265	C5_02530W_A	UAP1	-0.850317
orf19.1249	C4_05640C_A		-0.849861
orf19.4002	C5_05100C_A	DUN1	-0.849494
orf19.119	C6_01120C_A		-0.848047
orf19.3275	CR_00810W_A		-0.848013
orf19.5719	C6_03530C_A		-0.846995
orf19.3957	C5_04720C_A		-0.846445
orf19.4233	C5_02270W_A	THR4	-0.845374
orf19.3556	C2_05270W_A		-0.84505
orf19.4655	C4_01320C_A	OPT6	-0.843815
orf19.3204	C5_01710C_A		-0.842911
orf19.2694	C4_02980W_A	TYS1	-0.842245
orf19.6920	C7_01390W_A		-0.841952
orf19.7361	C3_05800W_A		-0.841828
orf19.4192.1	C6_00680C_A		-0.839854
orf19.4207	C6_00520W_A		-0.839399
orf19.288	C3_02950C_A	MET13	-0.839163
orf19.1730	C3_01210C_A		-0.838697
orf19.3831	C4_04520W_A		-0.838393
orf19.2214	C2_07680W_A		-0.837696
orf19.5428	C3_00390W_A		-0.836701

orf19.5921	C3_04630W_A		-0.836515
orf19.4538	C1_01850C_A		-0.836277
orf19.3740	CR_02280W_A	PGA23	-0.836079
orf19.1247	C4_05620C_A		-0.835635
orf19.680	C1_11220C_A	TIM50	-0.835277
orf19.6173	C3_07790W_A	STD1	-0.833404
orf19.6855	C1_04620W_A		-0.831147
orf19.4727	C1_08690W_A		-0.829869
orf19.7065	C7_00530C_A	PSF3	-0.82875
orf19.5238	C1_12250C_A		-0.827335
orf19.2555	CR_01650W_A	URA5	-0.826161
orf19.3787	C4_04920W_A		-0.825819
orf19.5658	C4_00310C_A	MNN10	-0.824559
orf19.2936	C1_02470W_A		-0.824307
orf19.4036	C5_05400W_A	APM1	-0.823311
orf19.6691	C7_03590C_A		-0.823097
orf19.2031	C2_00930C_A	VPS24	-0.822317
orf19.3142	C4_06670W_A		-0.819993
orf19.5143	C7_03120W_A	TIM54	-0.819498
orf19.1059	C1_04240C_A	HHF1	-0.819462
orf19.2143	C6_04530C_A		-0.818782
orf19.3100	C4_07080C_A		-0.818197
orf19.1296	C2_06650C_A		-0.817399
orf19.155	C2_04710C_A	URE2	-0.817151
orf19.5444	C3_00250C_A	TIM44	-0.817059
orf19.548	CR_04570C_A	CDC10	-0.815444
orf19.3365	C4_03380C_A	DAO2	-0.814155
orf19.7592	CR_10160W_A	FAA4	-0.813727
orf19.6369	CR_08100C_A	RIO2	-0.813383
orf19.182	C2_04820W_A		-0.812889
orf19.6345	C1_12770W_A	RPG1A	-0.812078
orf19.1853	CR_06810W_A	HHT2	-0.810379
orf19.4261	C5_02490C_A	TIF5	-0.809772
orf19.804	C2_04180C_A		-0.808876
orf19.3348	C1_01580W_A		-0.807573
orf19.7456	C3_06670C_A		-0.807455
orf19.506	CR_04200W_A	YDJ1	-0.807139
orf19.1753	C2_10270W_A	PUS7	-0.806339
orf19.7453	C3_06640W_A		-0.806338
orf19.145	C2_04650C_A	RPB4	-0.805972
orf19.4787	C1_09280W_A		-0.805667
orf19.6246	C1_06650W_A		-0.805665
orf19.2676	C4_03140C_A		-0.805354
orf19.6248	C1_06620C_A		-0.801282
orf19.4258	C5_02480W_A		-0.801208
orf19.5244	C1_12200W_A	MCD4	-0.800688

orf19.2639	C5_03410C_A		-0.800373
orf19.5255	C1_12100C_A	PXA2	-0.799787
orf19.7601	CR_10260W_A		-0.799002
orf19.2514	C3_01120W_A		-0.79638
orf19.4966	C1_13400C_A	AGC1	-0.79595
orf19.6436	CR_08620C_A		-0.795734
orf19.4164	C4_00770C_A		-0.795286
orf19.3170	C5_02010C_A		-0.794503
orf19.6769	C3_07290W_A		-0.794464
orf19.5682	C5_00150C_A		-0.794463
orf19.2599	CR_01980C_A	CRC1	-0.794149
orf19.2848	CR_02910W_A		-0.793941
orf19.6586	C7_01430C_A		-0.793794
orf19.652	CR_05010W_A		-0.791201
orf19.2798	C1_07470C_A		-0.78916
orf19.446.2	C1_05180C_A		-0.788321
orf19.1666	C3_01800C_A		-0.787983
orf19.7613	CR_10370W_A	HCR1	-0.787762
orf19.3931	C5_04440C_A	SFC1	-0.787344
orf19.7646	CR_10630W_A		-0.78573
orf19.5747	C6_03730C_A		-0.784747
orf19.3912	C5_04280C_A	GLN3	-0.783235
orf19.5958	C3_04890W_A	CDR2	-0.783056
orf19.6247	C1_06630W_A		-0.782434
orf19.2368	CR_06970C_A		-0.781986
orf19.5226	C1_12380C_A	WRS1	-0.781529
orf19.7486	CR_00480W_A	MRPL6	-0.781098
orf19.326	C3_03270W_A		-0.780479
orf19.939	C5_00500W_A	NAM7	-0.780009
orf19.3983	C5_04950C_A		-0.779681
orf19.3733	CR_02360W_A	IDP2	-0.779337
orf19.2643	C5_03360W_A	RPO26	-0.779211
orf19.7342	CR_09470W_A	AXL1	-0.779002
orf19.1238	C4_05570C_A	TUB4	-0.778504
orf19.4168	C4_00740W_A		-0.777715
orf19.3782.2	C4_04970C_A		-0.777432
orf19.1528	C2_02120W_A		-0.776284
orf19.4532	C1_01910W_A		-0.776167
orf19.6653	CR_05620C_A	MTG2	-0.77607
orf19.5485	C2_06220C_A	MEC3	-0.774617
orf19.5161	C7_03000C_A		-0.774524
orf19.5553	C6_02800W_A		-0.774253
orf19.3370	C4_03440C_A	DOT4	-0.773355
orf19.5294	C4_04150C_A	PDB1	-0.771558
orf19.585	C5_00820W_A		-0.770981
orf19.3207	C5_01680C_A	CCN1	-0.770586

orf19.4211	C6_00480C_A	FET31	-0.768563
orf19.6741	C3_07470W_A		-0.767947
orf19.7088	C7_00330C_A		-0.767909
orf19.6698	C7_03640C_A		-0.767385
orf19.2270	C2_07160W_A	SMF12	-0.767063
orf19.7610	CR_10340W_A	PTP3	-0.766956
orf19.7436.1	C3_06480C_A	ECM15	-0.766542
orf19.923	C5_00650C_A	THR1	-0.764918
orf19.5206	C2_05830C_A		-0.762855
orf19.570	C5_00710W_A	IFF8	-0.761961
orf19.6496	C7_02180C_A	TRS33	-0.761408
orf19.4788	C1_09290C_A	ARG5,6	-0.761239
orf19.2720	C4_02780W_A		-0.761082
orf19.7394	C3_06120C_A	GDA1	-0.759687
orf19.700	CR_06660W_A	SEO1	-0.758587
orf19.4301	C5_02780W_A		-0.757861
orf19.1624	C3_02210C_A		-0.757159
orf19.4292	C5_02720W_A	PEP12	-0.756185
orf19.7308	CR_09120C_A	TUB1	-0.755808
orf19.6939	C3_03770C_A		-0.755734
orf19.6751	C3_07400W_A		-0.755506
orf19.3894	C5_04120C_A		-0.755159
orf19.1801	C4_05450C_A	CBR1	-0.755012
orf19.4888	C1_10190W_A		-0.753847
orf19.4495	C2_04550C_A	NDH51	-0.752741
orf19.4809	C1_09460W_A	ERG12	-0.750226
orf19.1403	C2_09510C_A		-0.74988
orf19.702	CR_06680C_A		-0.749258
orf19.836.1	C2_03880C_A		-0.748612
orf19.6713	C3_07730W_A	WOR4	-0.748571
orf19.6029	C1_00770C_A	ROT1	-0.748362
orf19.2622	CR_07520C_A	YPT31	-0.748103
orf19.7012	C7_01020C_A		-0.746799
orf19.4705	C4_00880W_A		-0.74676
orf19.6242	C1_06690W_A	CYK3	-0.746576
orf19.4529	C1_01940C_A		-0.746365
orf19.4136	C5_01460W_A	YBL053	-0.742687
orf19.3893	C5_04110W_A	SCW11	-0.742362
orf19.1301	C4_03770W_A		-0.742335
orf19.4252	C5_02440C_A		-0.742074
orf19.6693	C7_03610C_A		-0.741337
orf19.3421.1	C6_01640W_A	MED19	-0.740438
orf19.7327	CR_09320C_A	PHO88	-0.740249
orf19.2820	CR_02610C_A		-0.738058
orf19.7663	CR_10740W_A	CSM1	-0.738014
orf19.2898	C4_06370C_A		-0.737245

orf19.7104	C7_00190W_A		-0.73445
orf19.2318.1	C1_10960W_A		-0.733869
orf19.1077	C6_04210C_A	ATM1	-0.731925
orf19.5870	C3_04270C_A	CTP1	-0.731905
orf19.5628	C6_03390W_A		-0.731616
orf19.1002	C1_10560C_A		-0.730781
orf19.6285	CR_07650W_A	GLC7	-0.730406
orf19.5305	C4_04050C_A	RHD3	-0.730344
orf19.3696	C7_02680W_A	TOM22	-0.729897
orf19.760	C1_04820C_A		-0.729709
orf19.1969	C5_01010W_A	CCW14	-0.729522
orf19.1849	CR_06840W_A		-0.72875
orf19.2369	CR_06960W_A		-0.728695
orf19.1522	C2_02050C_A		-0.728462
orf19.401	C1_08560W_A	TCP1	-0.728282
orf19.3357	C1_01680C_A		-0.727442
orf19.5231.2	C1_12320C_A	ATP19	-0.725954
orf19.3243	CR_01110W_A	SRP54	-0.725304
orf19.7223	C1_14160W_A		-0.725253
orf19.685.1	C6_01940W_A		-0.724335
orf19.4004	C5_05120W_A	CCT3	-0.721742
orf19.1491	C2_01820C_A		-0.721615
orf19.3419	C6_01670W_A	MAE1	-0.721597
orf19.4456	C1_07120W_A	GAP4	-0.72154
orf19.3684	C1_02270C_A		-0.720308
orf19.3457	C6_02170C_A	SWD3	-0.720086
orf19.2257	C2_07010W_A		-0.719444
orf19.7565	CR_09910W_A	GNP3	-0.719386
orf19.4867	C1_10010C_A	SWE1	-0.718517
orf19.3920	C5_04340W_A		-0.718118
orf19.171	CR_02530W_A	DBP2	-0.718042
orf19.5118	C1_08370W_A	SDS24	-0.716388
orf19.2799	C1_07460C_A	GPI8	-0.716114
orf19.3449.2	C6_01340C_A		-0.715788
orf19.5230	C1_12340C_A	MRPS9	-0.715788
orf19.5591	C6_03080C_A	ADO1	-0.715221
orf19.5073	C1_08010W_A	DPM1	-0.714506
orf19.4297	C5_02760W_A	CKB2	-0.713985
orf19.2198	C2_07830W_A	FLC3	-0.713946
orf19.3582	C2_05520W_A		-0.712672
orf19.2471	C1_05810W_A	GIM5	-0.712632
orf19.1954	C5_01180W_A	PUS4	-0.712124
orf19.7182	C7_03990C_A		-0.712041
orf19.3063	C1_03640C_A	HFL1	-0.711728
orf19.2506	C3_01020W_A		-0.711611
orf19.6924	C3_03910W_A	HTA1	-0.711014

orf19.6507	C7_02100W_A		-0.710925
orf19.3516	CR_05450C_A		-0.710896
orf19.2407	CR_03170W_A	DPS1-1	-0.710729
orf19.1792	C4_05340W_A		-0.710725
orf19.2782	C1_07630W_A		-0.708985
orf19.2788	C1_07570C_A		-0.708916
orf19.688	C6_01980C_A		-0.708494
orf19.3899	C5_04160W_A	SDC1	-0.708452
orf19.7186	C7_03940C_A	CLB4	-0.707623
orf19.5722	C6_03550C_A		-0.705631
orf19.863	C2_03560C_A		-0.704737
orf19.4133	C5_01430C_A		-0.702467
orf19.1272	C4_05850C_A		-0.702166
orf19.4182	C4_00610W_A		-0.700631
orf19.3088	C4_07150W_A		-0.700544
orf19.871	C2_03500W_A		-0.700279
orf19.3752	CR_02200C_A	RAD51	-0.697951
orf19.3103	C4_07060W_A		-0.69601
orf19.2914	C4_06240W_A		-0.695992
orf19.6463	C7_02460C_A		-0.69529
orf19.4180	C4_00630C_A	SEC72	-0.693396
orf19.2582	CR_01820W_A		-0.692975
orf19.3223.1	CR_01300W_A		-0.692496
orf19.6636	CR_05760C_A		-0.691775
orf19.3322	C1_01330C_A	DUT1	-0.691738
orf19.1711	C3_01400W_A	END3	-0.690162
orf19.4271	C5_02580W_A		-0.689087
orf19.2297	C1_11190W_A	ARL3	-0.688816
orf19.2538	CR_01520W_A	PTC2	-0.688418
orf19.443	C1_05230W_A		-0.688394
orf19.1793	C4_05350W_A		-0.687574
orf19.6346	C1_12760W_A		-0.686532
orf19.2954	C1_02650W_A		-0.686293
orf19.4018	C5_05250C_A		-0.685509
orf19.6752	C3_07390C_A		-0.684738
orf19.6240	C1_06700W_A		-0.68458
orf19.4600.1	C4_01920W_A	DPM3	-0.683537
orf19.6192	C3_07980C_A		-0.681941
orf19.2417	CR_03100W_A	SMC5	-0.680862
orf19.5720	C6_03540W_A		-0.680452
orf19.2520	CR_01370C_A		-0.679949
orf19.3288	C1_00990C_A	NMA111	-0.678986
orf19.3856	CR_06050W_A	CDC28	-0.678915
orf19.1625	C3_02190C_A		-0.678719
orf19.3129	C4_06800W_A		-0.67847
orf19.5085	C1_08110W_A		-0.67812

orf19.7485	CR_00490W_A		-0.677275
orf19.2236	C2_06830C_A	FHL1	-0.676304
orf19.5013	C1_13760W_A	AGM1	-0.675706
orf19.7673	CR_10820W_A		-0.675429
orf19.250	C3_02600C_A	SLC1	-0.675267
orf19.2389	CR_03340C_A		-0.674816
orf19.7097	C7_00250C_A		-0.674334
orf19.798	C2_04220C_A	TAF14	-0.672276
orf19.7513	CR_00210W_A	ALK2	-0.671765
orf19.2991	C1_03000W_A	HOL1	-0.670485
orf19.1549	C2_02310W_A		-0.669648
orf19.7500	CR_00330C_A	PXA1	-0.66362
orf19.4911	C1_10410W_A		-0.662397
orf19.2019	C2_01030W_A		-0.662045
orf19.6171	C3_07780C_A	NUP159	-0.661716
orf19.6293	CR_07590W_A	EMP24	-0.659746
orf19.3438	C6_01460C_A		-0.659122
orf19.5711	C6_03470W_A		-0.658953
orf19.3782	C4_04980W_A		-0.658916
orf19.6152	CR_07200W_A		-0.658815
orf19.7300	CR_09040W_A		-0.658142
orf19.5764	C6_03890C_A	SKI8	-0.657846
orf19.6717	C3_07700W_A		-0.657846
orf19.578	C5_00760W_A		-0.65611
orf19.4340	C5_03100C_A		-0.654808
orf19.2708	C4_02880C_A		-0.653309
orf19.6628	CR_05840W_A		-0.651996
orf19.1606	C3_02390W_A		-0.651813
orf19.1460	C2_01530C_A		-0.648764
orf19.3844	C4_04430W_A	MRP8	-0.647776
orf19.4326	C5_03010W_A		-0.647653
orf19.1429	C4_04210C_A	SOH1	-0.64756
orf19.6118	CR_07470W_A		-0.646357
orf19.6565	C7_01600W_A		-0.645881
orf19.6829	C3_06740W_A		-0.645298
orf19.7427	C3_06420C_A		-0.645138
orf19.1652	C3_01960C_A	POX1-3	-0.643512
orf19.1989	C2_01360C_A	DCW1	-0.642878
orf19.1967	C5_01050C_A		-0.641471
orf19.122	C6_01150W_A	CDC20	-0.639513
orf19.6155	C3_00830C_A		-0.63862
orf19.4641	C4_01440W_A	NMT1	-0.636911
orf19.1186	C6_00270W_A		-0.636716
orf19.1500	C2_01900C_A		-0.636611
orf19.56	C1_05020C_A	ARG2	-0.634175
orf19.184	C2_04800C_A	MDM10	-0.633158

orf19.5164	C7_02970W_A	ECM39	-0.633055
orf19.5496	C2_06330C_A	AVT1	-0.631629
orf19.2895	C4_06400C_A	VMA8	-0.63126
orf19.3240	CR_01140C_A	ERG27	-0.630738
orf19.4445	C1_07220W_A		-0.630213
orf19.5384	C3_00710W_A	CHS8	-0.629989
orf19.1710	C3_01410C_A	ALI1	-0.627338
orf19.991	C1_10480W_A	DJP1	-0.623331
orf19.2003	C2_01210C_A	HNM1	-0.623182
orf19.5884	C3_04370C_A		-0.619753
orf19.2008	C2_01160W_A		-0.613554
orf19.5410	C3_00530C_A		-0.612892
orf19.4306	C5_02820C_A		-0.612019
orf19.1621	C3_02240C_A	GPA2	-0.604466
orf19.3150	C2_06720W_A	GRE2	0.597615
orf19.6952	C3_03670W_A		0.61763
orf19.3839	C4_04470W_A	SAP10	0.618565
orf19.3845	C4_04420W_A	FGR3	0.619336
orf19.4731	C1_08730W_A		0.619413
orf19.4624	C4_01690C_A	HRT2	0.620124
orf19.349	C3_03510C_A		0.621628
orf19.6807	C3_06940W_A		0.624546
orf19.4614	C4_01790W_A		0.624606
orf19.2069	C2_00580C_A	SMF3	0.626017
orf19.1195	C6_00340C_A		0.628841
orf19.7494	CR_00390W_A	MMS22	0.63016
orf19.428	C1_05370C_A		0.630628
orf19.1281	C5_04080C_A		0.634105
orf19.1842	CR_06910W_A	BUD5	0.637167
orf19.6416	CR_08470W_A		0.637204
orf19.5580	C6_03010W_A	TEL1	0.637491
orf19.3736	CR_02330C_A	KAR4	0.643946
orf19.5312	C4_04000W_A	MET4	0.647428
orf19.7578	CR_10020C_A		0.647494
orf19.867	C2_03530W_A		0.6485
orf19.4278	C5_02620C_A		0.648717
orf19.4546	C1_01780C_A	HOL4	0.650387
orf19.2057	C2_00700W_A		0.650896
orf19.3422	C6_01650C_A	FMP27	0.651912
orf19.583	C5_00800C_A		0.655457
orf19.6638	CR_05740C_A	PTC4	0.656403
orf19.4376	CR_03780C_A		0.659691
orf19.2663	C1_06200W_A		0.660949
orf19.1526	C2_02100W_A	SNF2	0.662131
orf19.3369	C4_03430W_A	MOH1	0.663868
orf19.6578	C7_01510W_A		0.664028

orf19.4499	C2_04510W_A	RIM2	0.666832
orf19.4210	C6_00490W_A		0.667294
orf19.2484	C1_05670W_A		0.669722
orf19.84	C6_00830C_A	CAN3	0.670296
orf19.3447	C6_01370W_A		0.670364
orf19.1718	C3_01330W_A	ZCF8	0.671708
orf19.2653	C5_03260C_A		0.673286
orf19.856	C2_03620W_A	IFK2	0.6741
orf19.2110	C2_00230W_A		0.674103
orf19.4643	C4_01420W_A		0.675379
orf19.904	C2_03260W_A		0.675751
orf19.3878	CR_06260W_A		0.67619
orf19.4414	C4_06040W_A		0.679196
orf19.5880	C3_04360W_A		0.681704
orf19.55	C1_05010C_A		0.682444
orf19.5454	C3_00180C_A	DAL1	0.685353
orf19.3269	CR_00850C_A	GSL2	0.686628
orf19.1725	C3_01260C_A		0.686692
orf19.3795	C4_04840C_A	AGP3	0.68703
orf19.5952	C3_04840C_A		0.687435
orf19.1270	C4_05840W_A	FRE3	0.691823
orf19.5342	C2_10630W_A		0.693197
orf19.1843	CR_06900C_A	ALG6	0.6938
orf19.5422	C3_00440W_A		0.693986
orf19.7284	CR_08890C_A	ASR2	0.694929
orf19.5219	C1_12450C_A	IRA2	0.695994
orf19.5943	C3_04800C_A		0.696093
orf19.1796	C4_05390W_A		0.696586
orf19.3501	C6_02090C_A		0.696652
orf19.3030	C1_03330C_A		0.697692
orf19.7265	C1_14470W_A		0.698069
orf19.5625	C6_03360C_A		0.699622
orf19.6947	C3_03720W_A	GTT11	0.700493
orf19.2743	C4_02570C_A		0.701213
orf19.2135	C6_04500C_A	TSM1	0.70151
orf19.7379	C3_05980C_A	FAA2	0.704725
orf19.6929	C3_03860W_A		0.706541
orf19.1834	C1_10630C_A		0.706973
orf19.5816	C2_02880C_A	EBP7	0.708433
orf19.6344	C1_12780W_A	RBK1	0.708945
orf19.2952	C1_02630C_A	EXG2	0.709048
orf19.3521	C2_04880C_A	ARH2	0.709724
orf19.2972	C1_02840W_A	PDE2	0.710023
orf19.698	CR_06640C_A		0.710107
orf19.2397.3	CR_03260W_A		0.710782
orf19.3980	C5_04920C_A		0.711677

orf19.4229	C5_02220C_A		0.711707
orf19.4175	C4_00670W_A	TOK1	0.714996
orf19.5114	C1_08340C_A		0.7167
RDN5	CR_08760C_A	RDN5	0.716784
orf19.4880	C1_10110W_A		0.718125
orf19.7598	CR_10230W_A		0.721178
orf19.3059	C1_03560C_A	SUA71	0.722839
orf19.4269	C5_02560C_A		0.723164
orf19.7276.1	C1_14590C_A	TLO4	0.724236
orf19.994	C1_10510W_A		0.724352
orf19.4733	C1_08750W_A	YMC2	0.72527
orf19.5942	C3_04790W_A		0.728545
orf19.5605	C6_03180C_A		0.72868
orf19.465	CR_03880W_A	IFF9	0.729287
orf19.3701	C7_02630W_A		0.729429
orf19.7666	CR_10770W_A		0.729547
orf19.4900	C1_10300W_A	MNN12	0.732654
orf19.607	CR_07910C_A		0.733703
orf19.5841	C2_02660W_A		0.735096
orf19.508	CR_04210C_A	QDR1	0.735237
orf19.1424	C4_04250W_A		0.735422
orf19.1357	C2_09950W_A	FCY21	0.73572
orf19.3859	CR_06070W_A		0.737428
orf19.3712	CR_07830C_A		0.737699
orf19.1097	C6_04380W_A	ALS2	0.739095
orf19.261	C3_02690C_A		0.739892
orf19.4723	C1_08650C_A	FAD1	0.740228
orf19.1229	C1_07690C_A		0.740335
orf19.767	C1_04770C_A	ERG3	0.740555
orf19.4264	C5_02520W_A		0.740683
orf19.1656	C3_01900C_A		0.741801
orf19.7344	CR_09490W_A		0.743654
orf19.2769	C4_02340W_A		0.745101
orf19.4353	CR_03820C_A	ULP2	0.745674
orf19.1190	C6_00300C_A	STV1	0.746184
orf19.4753	C1_08950W_A	PFK26	0.747097
orf19.229	C3_02410C_A		0.747318
orf19.4953	C1_13270W_A		0.747948
orf19.3944	C5_04600C_A	GRR1	0.748085
orf19.5552	C6_02790C_A		0.748313
orf19.7498	CR_00360C_A	LEU1	0.749842
orf19.2133	C6_04490W_A	LIP4	0.752396
orf19.5604	C6_03170C_A	MDR1	0.753071
orf19.2605	CR_02040W_A	PRK1	0.762097
orf19.6592	CR_09700W_A		0.762165
orf19.5671	C4_00420C_A		0.762381

orf19.451	CR_06000W_A	SOK1	0.763267
orf19.7380	C3_05990C_A		0.763941
orf19.795	C2_04250W_A	VPS36	0.765598
orf19.6318	CR_04730W_A		0.766469
orf19.351	C3_03530W_A		0.766917
orf19.968	C5_00270W_A	PGA14	0.767292
orf19.2251	C2_06970W_A	AAH1	0.768729
orf19.292	C3_02980C_A		0.769665
orf19.7263	C1_14450C_A		0.770454
orf19.3234	CR_01200W_A	OYE22	0.770607
orf19.4883	C1_10140C_A		0.771502
orf19.2414	CR_03120W_A		0.772825
orf19.7611	CR_10350C_A	TRX1	0.773691
orf19.2051	C2_00730C_A		0.774401
orf19.610	CR_07890W_A	EFG1	0.775491
orf19.2879	C4_06550C_A	IFF5	0.776424
orf19.433	C1_05320C_A		0.77805
orf19.2965.1	C1_02770W_A		0.778123
orf19.7086	C7_00340C_A		0.780061
orf19.1192	C6_00320C_A	DNA2	0.782582
orf19.778	C1_04680W_A	PIL1	0.783776
orf19.434	C1_05300C_A	PRD1	0.78411
orf19.1961	C5_01090C_A		0.784476
orf19.5326	C2_10540W_A		0.784502
orf19.5284	CR_05420W_A		0.784794
orf19.6055	C1_00530C_A		0.785188
orf19.3355	C1_01650W_A	ISN1	0.787036
orf19.4967	C1_13410W_A	COX19	0.787777
orf19.2882	C4_06530C_A	XUT1	0.788124
orf19.4407	C4_05970W_A		0.792114
orf19.3318	C1_01290C_A		0.793116
orf19.4785	C1_09260C_A	PTC1	0.793401
orf19.3047	C1_03450C_A		0.794301
orf19.5291	C4_04180C_A		0.794646
orf19.6580	C7_01490W_A		0.795388
orf19.4756	C1_09000W_A		0.795966
orf19.1681	C3_01620W_A		0.796237
orf19.7487	CR_00470W_A		0.797568
orf19.612	CR_07870W_A		0.797959
orf19.918	C3_04070C_A	CDR11	0.799015
orf19.1888	C2_07430C_A		0.799037
orf19.2063	C2_00650W_A		0.799301
orf19.542.2	CR_04520W_A	MIM1	0.80233
orf19.1350	C2_08330W_A		0.804055
orf19.5439	C3_00300W_A		0.80547
orf19.3035	C1_03360W_A		0.805897

orf19.2310	C1_11050W_A		0.806962
orf19.4862.2	C1_09960W_A	PET100	0.807938
orf19.4779	C1_09210C_A		0.807969
orf19.2347	C1_10720C_A	MNN2	0.808458
orf19.4540	C1_01830C_A	UBC8	0.810635
orf19.4318	C5_02940C_A	MIG1	0.810822
orf19.4380.1	CR_03620C_A		0.812486
orf19.406	C1_08590C_A	ERG1	0.812555
orf19.449	C1_05160C_A		0.812789
orf19.4416	C4_06060W_A	VPS13	0.813246
orf19.4543	C1_01810C_A	UGA2	0.814671
orf19.4432	C1_07380C_A	KSP1	0.815563
orf19.1266	C4_05800C_A		0.815901
orf19.1871	C2_07560W_A	SWR1	0.81598
orf19.5651	C4_00260W_A		0.816625
orf19.3004	C1_03120W_A		0.820087
orf19.5558	C6_02840C_A	RBF1	0.820413
orf19.6793	C3_07070C_A		0.821062
orf19.376	C4_00080C_A		0.821427
orf19.589	CR_08060C_A	VPS21	0.821768
orf19.3029	C1_03320C_A	EHD3	0.82214
orf19.6754	C3_07370W_A		0.822777
orf19.5992	C3_05170W_A	WOR2	0.823428
orf19.2834	CR_02760C_A	RPD3	0.823759
orf19.4379	CR_03810W_A	PRP13	0.823811
orf19.7419	C3_06340W_A	HNT2	0.827176
orf19.791	C2_04290W_A	RIM11	0.829017
orf19.7140	C7_04280C_A		0.83027
orf19.4592	C4_02010C_A	HSX11	0.833052
orf19.6550	C7_01750W_A		0.83321
orf19.4282	C5_02650C_A	IFH1	0.833754
orf19.4982	C1_13510C_A	TGL99	0.834218
orf19.7261	C1_14440C_A	GDI1	0.835709
orf19.207	C2_08980C_A	PGA55	0.837261
orf19.3264	CR_00920W_A	CCE1	0.838727
orf19.7668	CR_10790W_A	MAL2	0.839572
orf19.333	C3_03360W_A	FCY2	0.839599
orf19.5980	C3_05090C_A		0.839602
orf19.4369	CR_03700C_A		0.839648
orf19.6842	C1_04480C_A	TUS1	0.840234
orf19.7204	C7_03780C_A		0.84162
orf19.3012	C1_03200C_A	ARO80	0.841869
orf19.6898	C7_01150W_A		0.842273
orf19.3148	C2_06740W_A		0.842533
orf19.6382	CR_08200C_A		0.844568
orf19.5688	C5_00090C_A		0.846798

orf19.7130	C7_04320W_A		0.847426
orf19.4720	C1_08620W_A	CTR2	0.847786
orf19.5068	C1_07970C_A	IRE1	0.849153
orf19.3610	C2_08660C_A		0.851163
orf19.1777	C2_10050W_A		0.851549
orf19.6923.1	C3_03920W_A		0.852094
orf19.7148	C7_04250W_A	TPO2	0.852956
orf19.7212	C1_14040W_A		0.854198
orf19.1782.1	C2_10000C_A		0.855641
orf19.5831	C2_02750C_A		0.859624
orf19.552	CR_04610C_A		0.860192
orf19.5003	C1_13650C_A		0.860462
orf19.5859	C3_04160W_A	DAL8	0.860985
orf19.3840	C4_04460C_A		0.861013
orf19.1798	C4_05420C_A	TSC2	0.862726
orf19.4608	C4_01850C_A	PDC12	0.863049
orf19.6800	C3_07010W_A	POS5	0.863285
orf19.2763	C4_02400C_A		0.863898
orf19.7319	CR_09210W_A	SUC1	0.864242
orf19.7046	C7_00730W_A	MET28	0.864942
orf19.5280	C1_11870W_A	MUP1	0.866501
orf19.2896	C4_06390W_A	SOU1	0.866771
orf19.1331	C7_03450C_A	HSM3	0.869155
orf19.48	C1_04950C_A	RPM2	0.871309
orf19.673	C1_11290W_A		0.871421
orf19.3156	C3_01180C_A		0.872337
orf19.163	CR_02580W_A	PAN6	0.872354
orf19.6660	C5_03510C_A		0.87476
orf19.1189	C6_00290W_A		0.877813
orf19.6038	C1_00670C_A	UGA32	0.878401
orf19.5102	C1_08230C_A	PLB5	0.87858
orf19.2826	CR_02670C_A		0.879445
orf19.3618	C2_08590W_A	YWP1	0.8814
orf19.5259	C1_12060C_A		0.881724
snR52	C3_07510C_A	SNR52	0.884316
orf19.4555	C6_04130C_A	ALS4	0.884369
orf19.6773	C3_07260C_A	ECM29	0.88446
orf19.2929	C1_02420C_A	GSC1	0.884735
orf19.7297	CR_09010C_A		0.885256
orf19.3858	CR_06060W_A		0.888888
orf19.6905	C7_01230C_A		0.889337
orf19.2253	C2_06990W_A		0.889344
orf19.6225	C1_06850W_A	PCL7	0.889908
orf19.2962	C1_02730W_A		0.889918
orf19.6393	CR_08290W_A		0.890074
orf19.2168	C2_08170W_A		0.89149

orf19.6065	C1_00420W_A		0.893527
orf19.3431	C6_01550C_A		0.895804
orf19.3218	C5_03940C_A		0.895907
orf19.6739	C3_07490W_A		0.899611
orf19.1960	C5_01100C_A	CLN3	0.900435
orf19.7355	C3_05740C_A	SSN8	0.90107
orf19.1153	C1_11660W_A	GAD1	0.902016
orf19.1668	C3_01770C_A		0.90211
orf19.4357	CR_03860C_A		0.903221
orf19.953.1	C5_00370W_A	COF1	0.903721
orf19.1069	C1_04330W_A	RPN4	0.90422
orf19.4673	C4_01170C_A	BMT9	0.904242
orf19.4912	C1_10420C_A		0.905572
orf19.7245	C1_14330W_A		0.905778
orf19.3483	C6_02420W_A		0.906391
orf19.3512	CR_05430W_A	CSP1	0.908685
orf19.6637	CR_05750W_A		0.909485
orf19.409	C1_08610C_A		0.909755
orf19.68.2	C1_12480W_A		0.911846
orf19.2302	C1_11140W_A		0.912188
orf19.4394	CR_03510W_A		0.912951
orf19.5063	C1_07900W_A	COI1	0.913346
orf19.5578	C6_02990W_A		0.91413
orf19.6875	C2_05650W_A	VPS35	0.915631
orf19.3753	CR_02190C_A	SEF1	0.916674
orf19.932	C5_00570W_A		0.918735
orf19.278	C3_02870C_A		0.919336
orf19.2033	C2_00910W_A	PGA19	0.919557
orf19.7400	C3_06320W_A	ALS7	0.922076
orf19.2242	C2_06880C_A	PRB1	0.922214
orf19.7405	C3_06270C_A		0.927341
orf19.432	C1_05330C_A		0.928412
orf19.1546	C2_02280W_A		0.928575
orf19.3340	C1_01520C_A	SOD2	0.92899
orf19.5065	C1_07930C_A	ERD1	0.929226
orf19.3007	C1_03140W_A		0.930294
orf19.4934	C1_13080W_A	OP4	0.930928
orf19.5879	C3_04350C_A		0.931624
orf19.2945	C1_02570C_A	PUT4	0.931681
orf19.5861.1	C3_04190W_A		0.932505
orf19.1277	C4_05900C_A		0.932851
orf19.3214	C5_03970W_A		0.93476
orf19.1763	C2_10180W_A	IFR1	0.936247
orf19.2990	C1_02990C_A	XOG1	0.936565
orf19.5537	C6_02670C_A	WSC2	0.936649
orf19.6313	CR_04800W_A	MNT4	0.937103

orf19.4821	C1_09580C_A	LIP1	0.940548
orf19.6995	C3_05600W_A	ATO6	0.940873
orf19.732	CR_07170W_A		0.944361
orf19.5204	C2_05810W_A		0.944924
orf19.2334	C1_10830W_A	BIG1	0.944993
orf19.2108	C2_00240C_A	SOD6	0.947591
orf19.6478	C7_02330W_A	YCF1	0.949408
orf19.5438	C3_00310C_A		0.950584
orf19.338	C3_03410C_A		0.950903
orf19.5335	C2_10570W_A	SGS1	0.950964
snR128	C1_08970W_A		0.951459
orf19.3861	CR_06080W_A	SIS1	0.953371
orf19.5622	C6_03340C_A	GLC3	0.954733
orf19.6824	C3_06790W_A	TRY6	0.955284
RDN58	CR_08790W_A	RDN58	0.955404
orf19.7583	CR_10070C_A	ZCF39	0.955433
orf19.6501	C7_02140W_A		0.956406
orf19.6736	C3_07550C_A		0.95662
orf19.6585	C7_01440W_A		0.957306
orf19.4494	C2_04560W_A	KTR2	0.957423
orf19.7349	C3_05700W_A	CHS4	0.958796
orf19.2842	CR_02850C_A	GZF3	0.959147
orf19.5801	C2_03010C_A	RNR21	0.960596
orf19.1193	C6_00330C_A	GNP1	0.962074
orf19.4377	CR_03790C_A	KRE1	0.963606
orf19.1481	C2_01700C_A	HAP42	0.964872
orf19.7522	CR_00130C_A		0.965113
orf19.7093	C7_00290C_A	HGT13	0.965693
orf19.6350	C1_12720C_A		0.966847
orf19.2332	C1_10850W_A		0.970276
orf19.410.3	C1_05560W_A	RIB4	0.972824
orf19.996	C1_10520W_A		0.975088
orf19.3139	C4_06710W_A		0.975376
orf19.6997	C3_05620W_A	ATO5	0.976944
orf19.5642	C4_00170W_A		0.981222
orf19.7173	C7_04060W_A		0.98537
orf19.6178	C3_07830W_A	FBP1	0.985505
orf19.5514	C6_02450W_A		0.986349
orf19.6420	CR_08510W_A	PGA13	0.986433
orf19.3021	C1_03270W_A		0.98647
orf19.2457	C1_05920W_A		0.987181
orf19.978	C5_00200C_A	BDF1	0.988124
orf19.4024	C5_05300W_A	RIB5	0.988705
orf19.7612	CR_10360C_A	CTM1	0.990572
orf19.225	C2_08850C_A		0.991556
orf19.4315	C5_02910C_A	GYP8	0.992538

orf19.86	C6_00850W_A		0.994188
orf19.3577	C2_05470W_A	COQ5	0.994494
orf19.1366	C2_09850C_A		0.995708
orf19.6678	C5_03650C_A		0.99904
orf19.7068	C7_00510W_A	MAC1	0.999525
orf19.1187	C6_00280W_A	CPH2	0.999882
orf19.1349	C2_08340C_A		1.00102
orf19.6977	C3_05390C_A	GPI1	1.00112
orf19.7372	C3_05920W_A	MRR1	1.00224
orf19.4792	C1_09320C_A		1.00281
orf19.2458	C1_05910W_A	SIP5	1.00339
orf19.21	C2_06430C_A		1.00382
orf19.5634	C4_00110C_A	FRP1	1.00629
orf19.5521	C6_02520W_A	ISA1	1.01122
orf19.2836	CR_02780W_A		1.01546
orf19.1307	C4_03710C_A		1.01601
orf19.5860	C3_04170W_A		1.01709
orf19.3802	C4_04780W_A	PMT6	1.01964
orf19.1720	C3_01310W_A		1.02156
orf19.2583	CR_01830C_A	PTR2	1.0239
orf19.3328	C1_01390C_A		1.02476
orf19.2613	CR_02130W_A	ECM4	1.02656
orf19.6756	C3_07350W_A		1.02678
orf19.3738	CR_02290W_A	PGA22	1.02679
orf19.5070	C1_07990C_A		1.02697
orf19.3040	C1_03390W_A	EHT1	1.02807
orf19.1795	C4_05370W_A	PUF3	1.03158
orf19.4898	C1_10280C_A		1.03334
orf19.7581	CR_10060W_A		1.03414
orf19.5601	C6_03150C_A		1.03671
orf19.2587	CR_01900C_A	HNM3	1.03927
orf19.4947	C1_13190W_A		1.04081
snR63	C2_05420C_A		1.04152
orf19.7570	CR_09960C_A	UGA3	1.04344
orf19.1486	C2_01750C_A		1.04721
orf19.2735	C4_02640C_A	SEN2	1.04762
orf19.4609	C4_01840C_A		1.04828
orf19.2481	C1_05690C_A		1.04886
orf19.6084	C1_00190C_A		1.05212
orf19.4824	C1_09610W_A		1.05223
orf19.3461	C6_02210W_A		1.05276
orf19.6544	C7_01780W_A	LPI9	1.05497
orf19.6557	C7_01670W_A		1.05665
orf19.5645	C4_00200C_A	MET15	1.05674
orf19.3544	C2_05130W_A		1.05967
orf19.4742	C1_08840W_A		1.06001

orf19.7389	C3_06080W_A	REV3	1.06048
orf19.5197	C1_04400C_A	APE2	1.0606
orf19.5059	C1_07880C_A	GCS1	1.06119
orf19.1861	C2_07640W_A		1.06133
orf19.2553	CR_01640C_A		1.06273
orf19.3621	C2_08580W_A		1.06397
orf19.3515	CR_05440W_A		1.06402
orf19.4309	C5_02860C_A	GRP2	1.06522
orf19.5672	C4_00430W_A	MEP2	1.06526
orf19.5250	C1_12140W_A		1.06591
orf19.1978	C5_00890C_A	GIT2	1.06633
orf19.670	C1_11330C_A	SMT3	1.06651
orf19.646	CR_05050W_A	GLN1	1.067
orf19.5978	C3_05080W_A		1.06892
orf19.1268	C4_05830W_A		1.06895
orf19.1564	C2_02410W_A		1.07082
orf19.5029	C1_13910C_A	MODF	1.07146
orf19.5867	C3_04250W_A	WSC1	1.07231
orf19.909	C2_03220C_A	STP4	1.07434
orf19.6798	C3_07020W_A	SSN6	1.07501
orf19.5933	C3_04730C_A		1.07572
orf19.87	C6_00860W_A	GPX1	1.07727
orf19.1926	C5_01400W_A	SEF2	1.08033
orf19.6288	CR_07610C_A		1.08475
orf19.2076	C2_00510W_A		1.08501
orf19.5383	C3_00720W_A	PMA1	1.08564
orf19.1837	C1_10660W_A	TBP1	1.08674
orf19.4371	CR_03720W_A	TAL1	1.08847
orf19.2552	CR_01630C_A		1.08931
orf19.413	C1_05520W_A		1.09047
orf19.5242	C1_12220W_A	CDC6	1.09078
orf19.860	C2_03600W_A	BMT8	1.09435
orf19.359	C3_03600C_A	GTT12	1.09469
orf19.4819	C1_09550W_A		1.09516
orf19.609	CR_07900C_A		1.09607
orf19.4747	C1_08880W_A	HEM14	1.09672
orf19.3971	C5_04850W_A		1.09803
orf19.7029	C7_00870W_A		1.09859
orf19.23	C2_06460W_A	RTA3	1.09888
orf19.5241	C1_12230W_A	SNT1	1.09934
orf19.4043	C5_05440C_A		1.09968
orf19.5257	C1_12080W_A	LCB4	1.10155
orf19.2601	CR_02000C_A	HEM1	1.10187
orf19.2812	C3_03980C_A		1.10815
orf19.4358	CR_03870W_A		1.1085
orf19.2272	C2_07170C_A	AFT2	1.11046

orf19.2344	C1_10740C_A	ASR1	1.11118
orf19.5169	C7_02920W_A		1.11127
orf19.5539	C6_02680W_A		1.11313
orf19.5346	C2_10680W_A		1.11527
orf19.3261	CR_00970W_A	ATO9	1.11753
orf19.6627	CR_05860W_A		1.1181
orf19.5285	CR_05390W_A	PST3	1.11822
orf19.5005	C1_13670W_A	OSM2	1.11899
orf19.2317	C1_10980W_A		1.11928
orf19.2341	C1_10780C_A	HNT1	1.12004
orf19.3707	CR_07790C_A	YHB1	1.12119
orf19.1562	C2_02390W_A		1.12148
orf19.2703	C4_02920W_A		1.12232
orf19.4044	C5_05450C_A	MUM2	1.12298
orf19.4070	C2_09120C_A		1.12402
orf19.1148	C1_11710C_A		1.12437
orf19.4736	C1_08780W_A		1.12441
orf19.4118	C2_06020W_A	CNT	1.12454
orf19.2021	C2_01010W_A	HGT8	1.12986
orf19.2107	C2_00260C_A	MUQ1	1.13132
orf19.4869	C1_10020W_A	SFU1	1.1322
orf19.781	C1_04650W_A	DUR3	1.13299
orf19.2637	C5_03460C_A		1.1342
orf19.1826	C1_06230C_A	MDM34	1.13507
orf19.7021	C7_00930W_A	GPH1	1.13749
orf19.7434	C3_06450W_A	GLG2	1.1389
orf19.2335	C1_10820C_A		1.13939
orf19.3644	C6_00800C_A		1.14011
orf19.6027	C1_00790W_A		1.14294
orf19.2517	CR_01340W_A		1.14425
orf19.388	C1_08430W_A	CAF16	1.14531
orf19.4550	C1_01750W_A		1.14809
orf19.3506	C6_02050W_A	DBR1	1.15064
orf19.419	C1_05440C_A		1.15131
orf19.6758	C3_07330W_A		1.15139
orf19.2079	C2_00480C_A	PHHB	1.15186
orf19.7192	C7_03900W_A		1.15233
orf19.7374	C3_05930W_A	CTA4	1.15424
orf19.1368	C2_09820W_A		1.15456
orf19.57	C1_05030C_A	PSF2	1.15832
orf19.5517	C6_02480W_A		1.1593
orf19.2176	C2_08080C_A	IFM3	1.16016
orf19.5777	C2_03170W_A		1.16226
orf19.4132	C5_01420W_A		1.16275
orf19.3926	C5_04400W_A	RNY11	1.165
orf19.2459	C1_05900W_A		1.16513

orf19.4017	C5_05240C_A		1.16526
orf19.4981	C1_13490C_A		1.16568
orf19.1290	C4_05300W_A	XKS1	1.16997
orf19.6141	CR_07270C_A	HGT16	1.17092
orf19.6311	CR_04820W_A		1.17093
orf19.553	CR_04620C_A		1.17096
orf19.2966	C1_02780W_A		1.1721
orf19.1150.1	C1_11680C_A		1.17248
orf19.4502	C2_04500W_A		1.17331
orf19.3713	CR_07840C_A		1.17423
orf19.6594	CR_09690C_A	PLB3	1.17899
orf19.6734	C3_07560W_A	TCC1	1.179
orf19.6001	C3_05230W_A	SAP3	1.18119
orf19.31	C2_06550W_A		1.18184
orf19.7320	CR_09220C_A	LIP7	1.1857
orf19.5144	C7_03110W_A	PGA28	1.18656
orf19.882	C2_03390C_A	HSP78	1.18695
orf19.5342.2	C2_10650W_A		1.18711
orf19.6805	C3_06950W_A		1.18763
orf19.2324	C1_10930C_A	UBA4	1.1887
orf19.7105	C7_00180W_A	FAR1	1.19025
orf19.3780	C4_05000W_A		1.19135
orf19.7313	CR_09170C_A	SSU1	1.19519
orf19.4975	C1_13450W_A	HYR1	1.19582
orf19.7539	CR_00050W_A	INO2	1.19741
orf19.4776	C1_09180W_A	LYS143	1.1985
orf19.1180	C6_00210W_A		1.19895
orf19.7247	C1_14340C_A	RIM101	1.19938
orf19.166	CR_02560C_A	ASG1	1.20043
orf19.1339	C7_03360W_A	CPY1	1.20311
orf19.6840	C1_04470C_A		1.20383
orf19.4979	C1_13470W_A	KNS1	1.20483
orf19.3302	C1_01140C_A		1.20626
orf19.35	C2_06600W_A		1.20776
orf19.849	C2_03710W_A		1.20783
orf19.2097	C2_00350W_A		1.20875
orf19.6117	CR_07480W_A		1.21074
orf19.2046	C2_00780W_A	POT1-2	1.21367
orf19.6059	C1_00490C_A	TTR1	1.21588
orf19.1748	C2_10300C_A		1.22087
orf19.6115	C1_00010W_A		1.22192
orf19.4816	C1_09520C_A		1.22247
orf19.4841	C1_09760C_A	SHY1	1.22473
SNRNAU4	C4_06180C_A		1.22529
orf19.771	C1_04740W_A	LPG20	1.22644
orf19.4929	C1_13010W_A		1.22662

orf19.513	CR_04250W_A		1.2267
orf19.6222	C1_06880C_A	SPO22	1.22738
orf19.3351	C1_01610C_A		1.22901
orf19.2772	C4_02300W_A	HOS3	1.22904
orf19.1336.2	C7_03380W_A		1.22963
orf19.279	C3_02880W_A		1.23174
orf19.1048	C1_04140W_A	IFD6	1.2348
orf19.2838	CR_02800C_A		1.23537
orf19.5925	C3_04650W_A		1.23769
orf19.4081	C2_09210W_A		1.23771
SCR1	CR_04275W_A	SCR1	1.23791
orf19.1381	C2_09710C_A		1.24114
orf19.2131	C6_04470C_A		1.24195
orf19.6486	C7_02270W_A	LDG3	1.24513
orf19.1369	C2_09810C_A		1.24736
orf19.4383	CR_03590C_A		1.24987
orf19.454	CR_05990C_A	SFL1	1.25002
orf19.5399	C3_00600W_A	IFF11	1.25055
orf19.2738	C4_02610C_A	SUL2	1.25099
orf19.2252	C2_06980W_A		1.25161
orf19.1654	C3_01940C_A		1.25182
orf19.3441	C6_01430C_A	FRP6	1.25191
orf19.5431	C3_00360W_A		1.25195
orf19.1417	C4_04300C_A		1.25306
orf19.3664	C1_02070W_A	HSP31	1.25559
orf19.4961	C1_13350W_A	STP2	1.25654
orf19.984	C1_10430W_A	PHO8	1.25727
orf19.3981	C5_04930C_A	MAL31	1.25779
orf19.2169	C2_08150W_A		1.25895
orf19.6211	C1_06970C_A		1.25898
orf19.1477	C2_01660C_A		1.25973
orf19.3863	CR_06100C_A		1.26046
orf19.6405	CR_08380C_A		1.26586
orf19.5962	C3_04930C_A	HGT4	1.26677
orf19.915	CR_06570C_A		1.26754
orf19.4056	C1_05140W_A	BRG1	1.26781
orf19.323	C3_03250W_A		1.26809
orf19.348	C3_03500W_A	SKN2	1.27018
orf19.3693	C1_02360C_A	PGA5	1.27352
orf19.775	C1_04700C_A		1.2779
orf19.4368	CR_03690W_A		1.27901
orf19.6755	C3_07360W_A	DLD2	1.28072
orf19.7609	CR_10330W_A	PGA11	1.28103
orf19.5612	C6_03250W_A	BMT4	1.28141
orf19.2809	C3_04000C_A	CTN3	1.28606
orf19.2886	C4_06480C_A	CEK1	1.28718

orf19.6261	C1_06520C_A	BPH1	1.29021
orf19.5213	C2_05910W_A		1.29224
orf19.5854	C3_04090W_A	SBP1	1.29291
orf19.4837	C1_09730W_A	DAM1	1.29359
orf19.7396	C3_06140W_A		1.29379
orf19.4539	C1_01840C_A		1.29484
orf19.2515	C3_01130C_A		1.29706
orf19.90	C6_00890W_A		1.29711
orf19.2244	C2_06890C_A		1.29918
orf19.7166	C7_04090C_A		1.30002
orf19.5919	C3_04610W_A	MEA1	1.30032
orf19.1461	C2_01540W_A		1.30171
orf19.5527	C6_02580W_A		1.30344
snR57	CR_06210C_A		1.30394
orf19.4279	C5_02630C_A	MNN1	1.30445
orf19.7580	CR_10040W_A		1.30534
orf19.1409.2	C4_04380C_A		1.30641
orf19.220	C2_08870C_A	PIR1	1.30717
orf19.6844	C1_04500W_A	ICL1	1.30816
orf19.1158	C1_11620W_A		1.3088
orf19.3781	C4_04990C_A		1.30891
orf19.6225.1	C1_06840C_A		1.30916
orf19.7479	CR_00560W_A	NTH1	1.30956
orf19.2280	C2_07230C_A	ZCF10	1.30975
orf19.2841	CR_02820W_A	PGM2	1.31649
orf19.4907	C1_10360C_A		1.31658
orf19.2006.1	C2_01180W_A	COX17	1.32205
orf19.1999	C2_01250W_A		1.32244
orf19.6349	C1_12730W_A	RVS162	1.32712
orf19.296	C3_03000W_A		1.32737
orf19.7296	CR_08990C_A		1.32852
orf19.1365	C2_09860C_A		1.32997
orf19.4804	C1_09420W_A	LIP2	1.33038
orf19.355	C3_03570C_A		1.33068
orf19.847	C2_03750W_A	YIM1	1.33101
orf19.446	C1_05200C_A		1.3321
orf19.2922	C4_06150C_A		1.33292
orf19.1171	C1_11490C_A	BTA1	1.33641
orf19.5526	C6_02570C_A	SEC20	1.33662
orf19.7295	CR_08980C_A		1.33991
orf19.1797	C4_05400C_A		1.34005
orf19.5954	C3_04870W_A		1.34439
RPR1	C2_08055W_A	RPR1	1.34545
orf19.2337	C1_10800C_A	ALP1	1.34718
orf19.951	C5_00390C_A		1.34764
orf19.4682	C4_01070W_A	HGT17	1.34807

orf19.2049	C2_00750W_A		1.34828
orf19.7561	CR_09880W_A	DEF1	1.34876
orf19.4322	C5_02970W_A	DAP2	1.34956
orf19.4041	C5_05430W_A	PEX4	1.35109
orf19.2655	C5_03240W_A	BUB3	1.35696
orf19.5862	C3_04200W_A	AFP99	1.35722
orf19.4196	C6_00610C_A		1.35985
orf19.5334	C2_10560C_A	ZSF1	1.36138
orf19.2946	C1_02580W_A	HNM4	1.36189
orf19.3915	C5_04300C_A		1.36467
orf19.5770	C6_03930W_A	OPT8	1.36732
orf19.5172	C7_02880C_A	LIP9	1.36798
orf19.1434	C2_08380C_A		1.36862
orf19.6396	CR_08300C_A		1.37015
orf19.7531	CR_00090C_A		1.37015
orf19.971	C5_00240W_A	SKN7	1.37103
orf19.295	C3_02990C_A		1.37266
orf19.5655	C4_00290C_A		1.37296
orf19.2296	C1_11200W_A		1.37733
orf19.4280	C5_02640W_A		1.37821
orf19.2132	C6_04480C_A		1.37853
orf19.5776	C2_03180C_A	TOM1	1.38232
orf19.952	C5_00380W_A		1.38421
orf19.4818	C1_09540W_A		1.38448
orf19.1974	C5_00930C_A	TFS1	1.38725
orf19.7151	C7_04220W_A		1.38957
orf19.4775	C1_09170W_A	CTA8	1.39055
orf19.2942	C1_02530C_A	DIP5	1.39254
orf19.398	C1_08530W_A		1.39493
orf19.4436	C1_07350C_A	GPX3	1.39663
orf19.2969	C1_02810W_A	RAD16	1.3969
orf19.4553	C6_04140C_A		1.39708
orf19.3172	C5_01990W_A		1.39888
orf19.113	C6_01070C_A	CIP1	1.39982
orf19.11	CR_07140C_A		1.40084
orf19.855	C2_03630W_A		1.40131
orf19.3932.1	C5_04480C_A		1.4021
orf19.4828	C1_09650W_A		1.40371
orf19.4099	C2_06170C_A	ECM17	1.40487
orf19.5611	C6_03240W_A		1.40584
orf19.6502	C7_02130W_A		1.40701
orf19.3320	C1_01310W_A		1.40897
orf19.3671	C1_02140C_A		1.40971
snR10c	C5_02850W_A		1.41009
orf19.5920	C3_04620C_A		1.41071
orf19.3940.1	C5_04560C_A	CUP1	1.41156

orf19.1035	C1_03740W_A	WAR1	1.41486
orf19.1827	C1_06220C_A		1.41495
orf19.2119	C2_00140W_A	NDT80	1.41592
orf19.5924	C3_04640W_A	ZCF31	1.41896
orf19.4477	C1_04020C_A	CSH1	1.4191
orf19.4013	C5_05200C_A		1.41965
orf19.1963	C5_01080C_A	GDS1	1.4244
orf19.6559	C7_01650W_A		1.42485
orf19.4015	C5_05220W_A	CAG1	1.42715
orf19.3910	C5_04260W_A		1.4298
orf19.2737	C4_02620C_A		1.44181
orf19.137	C6_01300W_A		1.44219
orf19.893	C2_03350W_A	PGA17	1.44382
orf19.7436	C3_06470W_A	AAF1	1.44391
orf19.7325	CR_09300C_A	SCO1	1.4485
orf19.3071	C3_00800W_A	MIH1	1.44863
orf19.6197	C1_07070C_A	DHH1	1.44944
orf19.5404.1	C3_00580W_A	FLO9	1.45504
orf19.1719	C3_01320C_A	SGA1	1.45739
orf19.69.2	C1_12460C_A		1.46108
orf19.7357	C3_05760W_A		1.46332
orf19.4786	C1_09270W_A	FGR43	1.46398
orf19.5078	C1_08060W_A	OFR1	1.46563
orf19.720	CR_06460W_A	GST3	1.46646
orf19.6232	C1_06780W_A	NPR1	1.46716
orf19.1285	C5_04050W_A		1.46862
orf19.1889	C2_07420W_A		1.47155
orf19.6659	C5_03500W_A	GAP6	1.47292
orf19.4738	C1_08800W_A		1.48383
orf19.4679	C4_01100C_A	AGP2	1.48424
orf19.4317	C5_02930C_A	GRE3	1.48479
orf19.2197	C2_07840W_A		1.49
orf19.3049	C1_03470C_A		1.49161
orf19.3395	C6_01870C_A		1.49829
orf19.1891	C2_07400C_A		1.50058
orf19.3353	C1_01630W_A		1.50067
orf19.6744	C3_07450C_A		1.50217
orf19.4444	C1_07230W_A	PHO15	1.50468
orf19.5282	C1_11850W_A		1.50807
orf19.6813	C3_06880W_A		1.51965
orf19.822	C2_04010C_A	HSP21	1.52032
orf19.1416	C4_04310W_A	COX11	1.5214
orf19.3656	C6_00720C_A	COX15	1.5261
orf19.733	CR_07160C_A		1.52629
orf19.2591	CR_01920W_A		1.52749
orf19.3661	C1_02040C_A		1.5311

orf19.1227	C1_07670W_A	ZCF4	1.53145
orf19.3548.1	C2_05180W_A	WH11	1.53161
orf19.5894	C3_04440C_A		1.53296
orf19.1321	C4_03570W_A	HWP1	1.53477
orf19.5636	C4_00130W_A	RBT5	1.53795
orf19.4057	C1_05130W_A		1.54237
orf19.3881	CR_06290C_A		1.54486
orf19.844	C2_03770C_A	STE11	1.551
orf19.4737	C1_08790W_A	TPO3	1.55558
orf19.2803	C3_04060C_A	HEM13	1.55672
orf19.1906	C2_00130W_A		1.56296
orf19.1159	C1_11610C_A		1.56303
orf19.7252	C1_14370W_A		1.56446
orf19.6527	C7_01940C_A		1.56572
orf19.2590	CR_01910C_A		1.56995
orf19.4246	C5_02380W_A		1.5731
orf19.5917	C3_04580C_A	STP1	1.57312
SNRNAU6	C3_05500W_A	SNR6	1.57392
orf19.4463	C1_03890W_A		1.57594
orf19.223	C2_08860W_A		1.57615
snR33a	CR_02470W_A		1.57616
orf19.2503	C3_00990C_A		1.57889
TER1	CR_05370W_A		1.57905
orf19.768	C1_04760C_A	SYG1	1.5803
orf19.4145	C5_01500C_A	ZCF20	1.58083
orf19.7027	C7_00880C_A		1.58132
orf19.158	C3_01150C_A		1.58199
orf19.3427	C6_01600W_A		1.58319
orf19.6640	CR_05720W_A	TPS1	1.58508
orf19.6817	C3_06850W_A	FCR1	1.59334
orf19.2047	C2_00770W_A		1.59348
orf19.3563	C2_05330C_A		1.5959
orf19.1768	C2_10130W_A		1.60562
orf19.6222.1	C1_06870C_A		1.60676
orf19.5408	C3_00550C_A	HRK1	1.60689
orf19.4531	C1_01920W_A	ROA1	1.60715
orf19.1237	C4_05560C_A	ARO9	1.6078
orf19.1671.1	C3_01740C_A		1.60845
orf19.2624	CR_07540C_A		1.60959
orf19.5557	C6_02830W_A	MNN4-4	1.6157
orf19.7336	CR_09390C_A		1.61817
orf19.5306	C4_04040W_A		1.61928
orf19.5812	C2_02930C_A		1.61982
orf19.3208	C5_01670W_A	DAL52	1.62166
orf19.344	C3_03460C_A		1.62317
orf19.5741	C6_03700W_A	ALS1	1.62363

orf19.7306	CR_09100C_A		1.62363
orf19.3038	C1_03380W_A	TPS2	1.62535
orf19.1107	C5_03870C_A		1.62799
orf19.3301	C1_01130W_A		1.62823
orf19.1325	C4_03540C_A	ECM38	1.62955
orf19.1887	C2_07440C_A		1.631
orf19.2892	C4_06420W_A		1.63264
orf19.1257	C4_05720W_A		1.63407
orf19.866	C2_03540W_A	RAD32	1.63492
orf19.3444	C6_01400W_A		1.63637
orf19.2681	C4_03100W_A	RBT7	1.641
orf19.5549	C6_02760W_A		1.64217
orf19.6949	C3_03700C_A		1.64641
orf19.218	C2_08880W_A	BUD20	1.64672
orf19.4370	CR_03710C_A		1.64835
orf19.3404	C6_01810W_A		1.649
orf19.2761	C4_02420C_A		1.65094
orf19.2073	C2_00540W_A		1.65117
orf19.4872	C1_10050W_A		1.65392
orf19.1844	CR_06870C_A		1.65418
orf19.5158	C7_03030W_A		1.65595
orf19.6091	C1_00150C_A	RIM8	1.65624
orf19.6658	C5_03490C_A		1.65661
orf19.3105	C4_07040W_A		1.65885
orf19.391	C1_08460C_A	UPC2	1.66037
orf19.4527	C1_01980W_A	HGT1	1.66264
orf19.823	C2_04000C_A		1.66347
orf19.4251	C5_02430W_A	ZCF22	1.66419
orf19.4843	C1_09780C_A		1.66601
orf19.7404	C3_06280W_A		1.66872
orf19.3924	C5_04380C_A		1.66913
orf19.1563	C2_02400W_A	ECM3	1.67326
orf19.2768	C4_02360W_A	AMS1	1.67355
orf19.4076	C2_09140C_A	MET10	1.6746
orf19.5469	C3_00030C_A		1.67992
orf19.97	C6_00960W_A	CAN1	1.68317
orf19.2474	C1_05770C_A	PRC3	1.68337
orf19.1958	C5_01120W_A		1.68775
orf19.431	C1_05340C_A	ZCF2	1.68901
orf19.2114	C2_00180C_A		1.702
orf19.6950	C3_03690W_A		1.70432
ITS2	CR_08800W_A	ITS2	1.7066
orf19.2583.2	CR_01850C_A		1.71368
orf19.4170	C4_00720W_A	CSP2	1.71953
orf19.812	C2_04090W_A		1.72094
orf19.1943	C5_01260W_A		1.72096

orf19.3826	C4_04560C_A		1.72336
orf19.2878	C4_06560W_A	PGA15	1.72671
orf19.3885	CR_06310W_A		1.72874
orf19.2531	CR_01470W_A	CSP37	1.72934
orf19.854	C2_03640W_A	UGA11	1.73237
orf19.4914.1	C1_12850W_A	BLP1	1.73433
orf19.5592	C6_03090W_A		1.73803
orf19.1028	C1_03810C_A	ELA1	1.73844
orf19.260	C3_02680C_A	SLD1	1.73973
orf19.5323	C2_10480W_A	MDH1-3	1.74138
orf19.1395	C2_09590C_A		1.74237
orf19.2989	C1_02980W_A	GOR1	1.74347
orf19.6138	CR_07300W_A		1.74673
orf19.2779	C1_07650W_A		1.74926
orf19.3368	C4_03420C_A		1.75338
orf19.1980	C5_00870C_A	GIT4	1.75543
orf19.1156	C1_11630C_A	FUS1	1.75594
orf19.1114	C5_03800W_A		1.76294
orf19.3589	C2_08820C_A	SPO11	1.7638
orf19.4287	C5_02690W_A		1.76704
orf19.489	CR_04060C_A	DAP1	1.76774
orf19.411	C1_05540C_A		1.77589
orf19.217	C2_08890W_A		1.78034
orf19.2175	C2_08100W_A		1.78799
orf19.2179	C2_08050C_A	SIT1	1.78962
orf19.4952	C1_13250W_A		1.79351
orf19.4942	C1_13150W_A		1.79464
orf19.2781	C1_07640C_A		1.79616
SNRNAU1	CR_03190C_A		1.79693
orf19.2839	CR_02810W_A	CIRT4B	1.80158
orf19.5417	C3_00480C_A	DOT5	1.80242
orf19.1679	C3_01640C_A		1.80329
orf19.4151	C5_01570C_A	SPO1	1.80938
orf19.2787	C1_07580C_A	PRY1	1.81911
orf19.6951	C3_03680W_A		1.82002
orf19.5843	CR_05610C_A	SRR1	1.821
orf19.6874	C2_05640W_A		1.83485
orf19.1586	C2_02600C_A	FGR22	1.83506
orf19.3655	C6_00730W_A		1.83599
orf19.3966	C5_04800W_A	CRH12	1.84416
orf19.5716	C6_03500C_A	SAP4	1.8492
orf19.691	C6_02010C_A	GPD2	1.8505
orf19.802	C2_04190C_A	UGA1	1.85205
orf19.1771	C2_10100W_A	CWC22	1.85356
orf19.1066	C1_04310C_A	GIG1	1.8554
orf19.6520	C7_02000C_A		1.86341

orf19.742	C4_05130C_A	ALD6	1.86409
orf19.7440	C3_06510C_A	HST6	1.86681
orf19.3278	CR_00780C_A	GSY1	1.86709
orf19.1009	CR_05280W_A		1.86739
orf19.4946	C1_13180W_A		1.86753
orf19.669	C1_11340W_A	PRM1	1.87701
orf19.1411	C4_04360W_A		1.88017
orf19.6838	C1_04460C_A		1.88359
orf19.3925	C5_04390C_A		1.88532
orf19.6058	C1_00500C_A	GLO1	1.89067
orf19.5686	C5_00100C_A		1.89296
orf19.7558	CR_09860W_A	YTA6	1.89508
orf19.7597	CR_10210W_A	PGA12	1.89684
orf19.1314	C4_03600C_A		1.90245
orf19.3413	C6_01730W_A	FGR37	1.90567
orf19.6937	C3_03800W_A	PTR22	1.90668
orf19.3969	C5_04830W_A	SFL2	1.9079
orf19.4566	C4_02240C_A		1.90927
orf19.1120	C5_03750W_A	FAV2	1.90967
orf19.3134	C4_06750C_A		1.91541
orf19.6742	C3_07460W_A		1.91923
orf19.6608	CR_09530C_A		1.92078
orf19.6449	CR_08720W_A		1.92192
orf19.942	C5_00470C_A	KRE62	1.92331
orf19.7053	C7_00660W_A	GAC1	1.92545
orf19.5818	C2_02860W_A	SUR2	1.92637
orf19.2107.1	C2_00250W_A	STF2	1.92663
orf19.7550	CR_09780C_A	IFA14	1.93186
orf19.5227	C1_12370W_A		1.93204
orf19.3325	C1_01360C_A		1.93319
orf19.6214	C1_06940C_A	ATC1	1.93379
orf19.7314	CR_09180W_A	CDG1	1.93581
orf19.5140	C7_03150W_A		1.93873
orf19.4424	C1_07430W_A	PHO100	1.94052
orf19.5524	C6_02550W_A		1.94071
orf19.6070	C1_00390W_A	ENA2	1.94111
orf19.2968	C1_02800W_A		1.94351
orf19.695	CR_06590C_A	RGS2	1.94583
orf19.5000	C1_13630W_A	CYB2	1.94648
orf19.3820	C4_04620C_A		1.96664
orf19.3131	C4_06780C_A	OYE32	1.96802
orf19.4528.1	C1_01970W_A		1.97546
orf19.69	C1_12470W_A		1.9755
orf19.1032	C1_03770W_A	SKO1	1.97694
orf19.6003	C3_05250C_A		1.97856
orf19.1207	C4_03970W_A		1.98019

orf19.3999	C5_05070W_A		1.98034
orf19.3140	C4_06700W_A		1.98054
orf19.4943	C1_13160W_A	PSA2	1.98456
orf19.4853	C1_09870W_A	HCM1	1.98483
orf19.5045	C4_03890W_A	PTP2	1.98644
orf19.6263	C1_06510C_A		1.98804
orf19.5542	C6_02710C_A	SAP6	2.00389
orf19.7503	CR_00300W_A	CDA2	2.01355
orf19.5228	C1_12360C_A	RIB3	2.01449
orf19.744	C4_05140C_A	GDB1	2.01624
orf19.4551	C1_01740W_A	CTN1	2.02357
orf19.584	C5_00810C_A		2.03103
orf19.5915	C3_04570C_A	DUR35	2.03422
orf19.7437	C3_06490W_A		2.03983
orf19.2881	C4_06540W_A	MNN4	2.04614
orf19.2608	CR_02070C_A	ADH5	2.04954
orf19.3104	C4_07050W_A	YDC1	2.05218
orf19.4784	C1_09250W_A	CRP1	2.05834
orf19.6164	C3_00870W_A		2.05966
orf19.5103	C1_08240C_A		2.06217
orf19.5474	C3_00010C_A		2.06249
orf19.5902	C3_04480C_A	RAS2	2.06403
orf19.1075	C6_04190C_A		2.06661
orf19.2866	C4_06660W_A		2.06775
orf19.3746	CR_02240C_A	OPT2	2.06968
orf19.1152	C1_11670W_A		2.07022
orf19.4580	C4_02110W_A		2.07768
orf19.1075.1	C6_04200C_A		2.08012
orf19.723	CR_06440C_A	BCR1	2.08114
SNRNAU2	C2_04070C_A		2.08814
orf19.1476	C2_01650W_A		2.09027
orf19.868	C2_03520C_A	ADAEC	2.09336
orf19.550	CR_04590C_A	PDX3	2.0993
orf19.1240	C4_05590W_A		2.10047
orf19.3888.2	CR_06350C_A		2.11007
orf19.33	C2_06570C_A		2.12189
orf19.3337	C1_01510W_A		2.12503
orf19.7596	CR_10200W_A		2.12733
orf19.5911	C3_04550C_A	CMK1	2.12851
orf19.7323	CR_09270C_A	CBP1	2.13109
orf19.2467	C1_05840W_A	PRN1	2.1343
orf19.6387	CR_08250C_A	HSP104	2.14077
orf19.6484	C7_02280W_A		2.14188
orf19.7557	CR_09850C_A	FGR46	2.14706
orf19.1353	C2_08300C_A		2.14884
orf19.2823	CR_02640W_A	RFG1	2.15618

orf19.1027	C1_03820W_A	PDR16	2.1562
orf19.6992	C3_05570W_A	QDR2	2.16064
orf19.450	C1_05150C_A		2.16335
orf19.2430	C1_06150W_A		2.17032
orf19.4774	C1_09160W_A	AOX1	2.17783
orf19.1582	C2_02570W_A		2.18027
orf19.238	C3_02480C_A	CCP1	2.18229
orf19.2652	C5_03270W_A	TEF4	2.18516
orf19.1060	C1_04250C_A		2.18665
orf19.4752	C1_08940C_A	MSN4	2.19049
orf19.2429	C1_06160W_A		2.19251
orf19.6747	C3_07430W_A		2.21083
orf19.4690	C4_00990W_A		2.21465
orf19.5023	C1_13850C_A	DAL7	2.21539
orf19.5729	C6_03610W_A	FGR17	2.22131
orf19.22.1	C2_06450C_A		2.22303
orf19.7283	CR_08880C_A		2.22539
orf19.4777	C1_09190C_A	DAK2	2.22673
orf19.430	C1_05350W_A	YPT53	2.22968
orf19.4048	C5_05480W_A	DES1	2.23523
orf19.5037	C4_03820C_A	HRQ2	2.23707
orf19.4856	C1_09900W_A	LIP3	2.24733
orf19.6540	C7_01800C_A	PFK2	2.24765
orf19.1105.3	C5_03890C_A		2.26328
orf19.2010	C2_01140C_A		2.26782
orf19.4216	C5_02110W_A		2.27146
orf19.4976	C1_13460W_A		2.27382
orf19.6142	CR_07260C_A		2.29564
orf19.5616	C6_03280W_A		2.29571
snR189b	C2_04610C_A		2.29761
orf19.6983	C3_05450C_A		2.30447
orf19.5866	C3_04240C_A		2.30643
orf19.3310	C1_01220C_A		2.31785
orf19.3121	C4_06900W_A	GST1	2.31898
orf19.1106	C5_03880C_A		2.34783
orf19.3888	CR_06340C_A	PGI1	2.34925
orf19.6257	C1_06550W_A	GLT1	2.3615
orf19.3127	C4_06820C_A	CZF1	2.37199
orf19.1667.1	C3_01780C_A		2.37481
orf19.6450	CR_08740W_A		2.37987
orf19.3869	CR_06140W_A		2.37989
orf19.5760	C6_03850C_A	IHD1	2.38156
orf19.4886	C1_10170W_A		2.38769
orf19.2261	C2_07060W_A		2.3886
orf19.2602	CR_02020C_A	OPT1	2.38972
orf19.3364	C4_03370C_A		2.39665

orf19.6077	C1_00310W_A		2.39805
orf19.1691	C3_01540W_A		2.39879
orf19.3160	C5_02080C_A	HSP12	2.39966
ITS1	CR_08780W_A	ITS1	2.40545
orf19.322	C3_03240C_A		2.41064
orf19.1169	C1_11510C_A		2.42104
orf19.5525	C6_02560W_A		2.43428
orf19.7196	C7_03860W_A		2.44314
orf19.6398	CR_08310C_A		2.46362
orf19.1067	C1_04320W_A	GPM2	2.46469
orf19.3988	C5_04980W_A		2.46711
orf19.7225	C1_14180W_A		2.47061
orf19.6079	C1_00270W_A		2.47454
orf19.6882	C2_05700W_A	OSM1	2.47614
orf19.4581	C4_02100C_A	GPI14	2.47997
orf19.4836	C1_09720W_A	URA1	2.48132
orf19.1867	C2_07580W_A		2.48676
orf19.6078	C1_00290W_A	POL93	2.48741
orf19.6816	C3_06860C_A		2.49612
orf19.4980	C1_13480W_A	HSP70	2.50233
orf19.3442	C6_01420C_A		2.50937
orf19.842	C2_03790C_A	ASR3	2.53938
orf19.1600	C2_09420W_A		2.54937
orf19.251	C3_02610C_A	GLX3	2.55071
orf19.3612	C2_08640C_A	PST2	2.554
orf19.3263	CR_00930W_A	ATO10	2.55693
orf19.1756	C2_10240W_A	GPD1	2.55778
orf19.1778	C2_10040W_A		2.58322
orf19.2584	CR_01860W_A	OPT9	2.5841
orf19.2725	C4_02730C_A		2.58962
orf19.6556	C7_01680C_A		2.59225
orf19.6224	C1_06860W_A		2.59729
orf19.647.3	CR_05040W_A		2.60078
orf19.3822	C4_04590W_A	SCS7	2.60833
orf19.633	CR_04960C_A	CRG1	2.62501
orf19.769	C1_04750W_A	IFE1	2.63742
orf19.4617	C4_01760W_A		2.63841
orf19.1979	C5_00880C_A	GIT3	2.64005
orf19.2766	C4_02380W_A		2.6495
orf19.776	C1_04690C_A		2.65133
orf19.5145	C7_03090C_A	SSP96	2.65818
orf19.5447	C3_00220W_A	HGT19	2.65973
orf19.6998	C3_05630W_A	GTT1	2.676
orf19.4391	CR_03480W_A		2.67832
orf19.1863	C2_07620W_A		2.68631
orf19.3522	C2_04910W_A		2.68919

orf19.5180	C7_02810W_A	PRX1	2.69962
orf19.258	C3_02660W_A		2.69986
orf19.2496	C3_00930W_A	ATO2	2.73066
orf19.5503	C7_03740C_A		2.7333
orf19.5820	C2_02850W_A	UGA6	2.73673
orf19.4842	C1_09770W_A		2.73838
orf19.1616	C3_02300W_A	FGR23	2.73858
orf19.542	CR_04510W_A	HXK2	2.75783
orf19.2262	C2_07070W_A		2.76967
orf19.5288	CR_05340C_A	IFE2	2.77661
orf19.1286	C5_04040C_A		2.77668
orf19.6757	C3_07340W_A	GCY1	2.7952
orf19.4530.1	C1_01930W_A		2.79721
orf19.2395	CR_03290C_A	IME2	2.81299
orf19.5842	C2_02650C_A		2.83632
orf19.3967	C5_04810W_A	PFK1	2.85893
orf19.3378	C4_03500C_A		2.86186
orf19.4310	C5_02870C_A		2.87629
orf19.5844	CR_05580C_A		2.88112
orf19.7356	C3_05750C_A		2.89172
orf19.6487	C7_02260W_A		2.89218
orf19.2691	C4_03000C_A		2.91043
orf19.2172	C2_08130W_A	ARA1	2.91488
orf19.5713	C6_03480W_A	YMX6	2.92201
orf19.308	C3_03110W_A		2.93574
orf19.5113	C1_08330C_A	ADH2	2.93823
orf19.5520	C6_02510C_A	ASG7	2.94116
orf19.36.1	C2_06630C_A		2.97689
orf19.5635	C4_00120W_A	PGA7	2.9852
orf19.539	CR_04480C_A	LAP3	2.98548
orf19.6488	C7_02250W_A		3.00988
orf19.6116	CR_07490C_A	GLK4	3.01568
orf19.4461	C1_03880C_A		3.03471
orf19.1275	C4_05880W_A	GAT1	3.03543
orf19.3711	CR_07820W_A		3.04133
orf19.6661	C5_03520W_A		3.0461
orf19.7251	C1_14360C_A	WSC4	3.05209
orf19.5348	C2_10690W_A	TPS3	3.05234
orf19.1581	C2_02560W_A		3.05806
orf19.4833	C1_09690W_A	MLS1	3.08327
orf19.164	CR_02570C_A		3.08964
orf19.3749	CR_02220C_A	OPT3	3.10441
orf19.7227	C1_14190C_A		3.118
orf19.2530	CR_01460C_A		3.12254
orf19.903	C2_03270W_A	GPM1	3.12288
orf19.347	C3_03490W_A	RSN1	3.1521

snR42a	C1_07450W_A		3.24152
orf19.2248	C2_06940C_A	ARE2	3.25789
orf19.1287	C5_04030W_A		3.2725
orf19.1433	C2_08390W_A		3.28236
orf19.5811	C2_02940W_A	MET1	3.28759
orf19.2371	C7_02610C_A		3.29494
orf19.3432	C6_01520W_A		3.31745
orf19.1433.1	C2_08400C_A		3.3383
orf19.1034	C1_03750W_A		3.35091
orf19.6745	C3_07440W_A	TPI1	3.355
orf19.111	C6_01060C_A	CAN2	3.35897
orf19.3651	C6_00750C_A	PGK1	3.36101
orf19.689	C6_01990W_A	PLB1	3.40248
orf19.787.1	C4_03960W_A		3.54404
orf19.125	C6_01180C_A	EBP1	3.6245
orf19.3803	C4_04770C_A	MNN22	3.63516
orf19.3053	C1_03510C_A		3.63713
orf19.5663	C4_00360C_A	RCH1	3.63946
orf19.3117	C4_06920C_A	CSA2	3.66857
orf19.1614	C3_02310W_A	MEP1	3.6879
orf19.5759	C6_03840C_A	SNQ2	3.70629
orf19.2619	CR_02180W_A	PHO113	3.74423
orf19.1862	C2_07630C_A		3.77395
orf19.2762	C4_02410C_A	AHP1	3.81132
orf19.2693	C4_02990C_A	GST2	3.84425
orf19.7676	CR_10840C_A	XYL2	3.85487
orf19.7054	C7_00650W_A		3.85982
orf19.4773	C1_09150W_A	AOX2	3.86096
orf19.5079	C1_08070W_A	CDR4	3.86886
orf19.1149	C1_11700C_A	MRF1	3.8831
orf19.5437	C3_00320W_A	RHR2	3.88807
orf19.2048	C2_00760C_A		3.90413
orf19.4438	C1_07330W_A	RME1	3.95272
orf19.1831	C1_10600C_A		3.9531
orf19.4716	C4_06120W_A	GDH3	3.96535
orf19.5785	C2_03110W_A		4.0196
orf19.1599	C2_09410W_A		4.08738
orf19.3433	C6_01510W_A	OYE23	4.10794
orf19.3727	CR_02400W_A	PHO112	4.15186
orf19.2372	C7_02600C_A		4.2063
orf19.7278	C1_14630C_A		4.21061
orf19.2941	C1_02520W_A	SCW4	4.21818
orf19.5742	C6_03710W_A	ALS9	4.27947
orf19.6391	CR_08270W_A		4.34432
orf19.780	C1_04660W_A	DUR1,2	4.36522
orf19.5674	C4_00450C_A	PGA10	4.39421

orf19.4612	C4_01800W_A			4.45555
orf19.734	CR_07150W_A	GLK1		4.45938
orf19.6993	C3_05580C_A	GAP2		4.99481
orf19.6209	C1_06980C_A			4.99661
orf19.5784	C2_03120W_A	AMO1		5.12644
orf19.2350	C1_10710C_A			5.4047
orf19.1868	C2_07570W_A	RNR22		5.53888
orf19.7495	CR_00380W_A			5.84608
orf19.7397.3	C3_06170C_A			6.05348
orf19.3120	C4_06910W_A			6.93616
orf19.1438	C4_03340C_A			9.70693
orf19.7417	C3_06180C_A	TSA1		10.0054
orf19.3868	CR_06130C_A		inf	
snR60	C1_05550C_A		inf	
tA(UGC)1	C6_01800W_A	tA(UGC)1	inf	
tV(CAC)1	C2_00040C_A	tV(CAC)1	inf	
