# Supplementary material for: Inhibition of Classical and Alternative Modes of Respiration in Candida albicans Leads to Cell Wall Remodeling and Increased Macrophage Recognition
Source: mBio. 2019 Jan 29;10(1):e02535-18. doi: 10.1128/mBio.02535-18 (PMC6355986; doi:10.1128/mBio.02535-18)
Supplement: TABLE S4 [file mBio.02535-18-st004.pdf]

Untreated vs SNP: differentially expressed genes

Assembly 21 Identifier	Systematic Name	Standard Name	Log[2]Fold Change
orf19.2061	C2_00670C_A		-6.30499
orf19.6249	C1_06610C_A	HAK1	-5.20699
orf19.4691	C4_00980C_A	MRV1	-4.67835
orf19.265	C3_02750W_A		-4.51663
orf19.4653	C4_01340W_A		-4.38052
orf19.3107	C4_07020C_A		-4.21929
orf19.6302	CR_04900C_A	PGA39	-4.21043
orf19.4384.1	CR_03580C_A		-4.11461
orf19.2633.1	CR_07740W_A		-4.08352
orf19.1774	C2_10070W_A		-4.07643
orf19.2633	CR_07730W_A	HGT14	-3.8154
orf19.4342	C5_03130W_A	SUT1	-3.75821
orf19.5302	C4_04080C_A	PGA31	-3.75672
orf19.7514	CR_00200W_A	PCK1	-3.67482
orf19.2060	C2_00680C_A	SOD5	-3.63402
orf19.4789	C1_09300C_A		-3.61332
orf19.4934	C1_13080W_A	OP4	-3.47781
orf19.1766	C2_10150W_A		-3.44347
orf19.3352	C1_01620C_A		-3.43675
orf19.356	C3_03580C_A	GTT13	-3.43519
orf19.6248	C1_06620C_A		-3.428
orf19.4654	C4_01330W_A		-3.40689
orf19.3721	CR_02440W_A		-3.30781
orf19.2049	C2_00750W_A		-3.29325
orf19.10	CR_07130C_A	ALK8	-3.28814
orf19.2475	C1_05760C_A	PGA26	-3.26364
snR77b	C4_01630C_A		-3.22835
orf19.1724	C3_01270W_A		-3.22143
orf19.716	CR_06500C_A		-3.19127
orf19.4179	C4_00640W_A		-3.11261
orf19.183	C2_04810W_A	HIS3	-3.06881
orf19.670.2	C1_11320C_A		-3.0599
orf19.3109	C4_07000W_A		-3.04516
orf19.7098	C7_00240W_A		-3.02999
orf19.1930	C5_01380W_A	CFL5	-2.90579
orf19.94	C6_00930C_A		-2.90069
orf19.6688	C7_03560W_A		-2.88118
orf19.1530	C2_02140C_A		-2.86888
orf19.2846	CR_02880W_A		-2.85279
orf19.3112	C4_06970C_A	ZRT1	-2.83503
orf19.3238	CR_01160W_A		-2.83177
orf19.944	C5_00450C_A	IFG3	-2.81795
orf19.6245	C1_06660W_A		-2.79855
orf19.1450	C2_01460C_A		-2.7689
orf19.7585	CR_10100C_A	INO1	-2.76319
orf19.5212	C2_05900W_A		-2.75773

orf19.5814.1	C2_02910W_A		-2.72706
orf19.6899	C7_01170C_A		-2.68321
orf19.4698	C4_00960W_A	PTC8	-2.68193
orf19.715	CR_06510W_A		-2.6722
orf19.532	CR_04420C_A	RBR2	-2.67074
orf19.851	C2_03690C_A		-2.63458
orf19.3113	C4_06960W_A		-2.6288
orf19.1611	C3_02330C_A		-2.61465
orf19.1822	C1_06280C_A	UME6	-2.61352
orf19.787	C4_03950C_A		-2.59702
orf19.4921.1	C1_12910W_A		-2.57586
orf19.8278	C1_11410C_A		-2.56495
orf19.255	C3_02640C_A	ZCF1	-2.53522
orf19.1964	C5_01070C_A		-2.51548
orf19.3114	C4_06950W_A		-2.50745
orf19.5905	C3_04510W_A		-2.49749
orf19.1523	C2_02060C_A	FMO1	-2.47724
orf19.4970	C1_13430C_A		-2.46134
orf19.675.1	C1_11260C_A		-2.45224
orf19.1815	CR_07080W_A		-2.41523
orf19.2202	C2_07790C_A		-2.40878
orf19.889	C2_03370W_A	THI20	-2.40563
orf19.6864	C4_05250W_A		-2.40014
orf19.3722	CR_02430C_A		-2.39934
orf19.4873	C1_10060C_A		-2.39918
orf19.5504	C7_03730C_A		-2.39036
orf19.1415	C4_04320W_A	FRE10	-2.38536
orf19.1580	C2_02550C_A		-2.38078
orf19.4212	C6_00470C_A	FET99	-2.37244
orf19.5132	C7_03230C_A		-2.36548
orf19.5975	C3_05050W_A	TRY4	-2.36468
orf19.3794	C4_04850C_A	CSR1	-2.35744
orf19.2253	C2_06990W_A		-2.35243
orf19.3932	C5_04470C_A		-2.3523
orf19.7447	C3_06580W_A	JEN1	-2.31917
orf19.2062	C2_00660C_A	SOD4	-2.31305
orf19.2445	C1_06000W_A		-2.30931
CaalfMr17	CM_00360W	RRNS	-2.30276
orf19.3954.1	C5_04690C_A		-2.30158
orf19.6919	C7_01380W_A		-2.27388
orf19.3110	C4_06990W_A		-2.26022
orf19.2044	C2_00800C_A	PGA27	-2.25656
orf19.2770.1	C4_02320C_A	SOD1	-2.25029
orf19.4506	C2_04460W_A	LYS22	-2.23956
orf19.5831	C2_02750C_A		-2.22778
orf19.4568	C4_02220C_A	ZCF25	-2.21121
orf19.6948	C3_03710W_A	CCC1	-2.20387
orf19.7504	CR_00290W_A		-2.1886

orf19.1344	C7_03310W_A		-2.18301
orf19.1080	C6_04230W_A		-2.18192
orf19.1996	C2_01270W_A	CHA1	-2.18104
orf19.3895	C5_04130C_A	CHT2	-2.18088
orf19.1440.1	C4_03310C_A		-2.18067
orf19.5133	C7_03220C_A	ZCF29	-2.1704
orf19.6090	C1_00160C_A		-2.15321
orf19.4266	C5_02540C_A	SPR28	-2.14445
orf19.1541	C2_02230C_A		-2.14437
orf19.7455	C3_06660C_A		-2.12671
orf19.1874	C2_07530C_A		-2.12155
orf19.1524	C2_02070W_A	SPR3	-2.11732
orf19.2770	C4_02330C_A		-2.11121
orf19.1473	C2_01630W_A		-2.10272
orf19.6920	C7_01390W_A		-2.09914
orf19.4600	C4_01930C_A		-2.09843
orf19.4690	C4_00990W_A		-2.0822
orf19.3115	C4_06940C_A		-2.08077
orf19.3902	C5_04190W_A	MRV2	-2.07018
orf19.1148	C1_11710C_A		-2.06251
orf19.7411	C3_06220C_A	OAC1	-2.05937
orf19.1123	C5_03720C_A		-2.0489
orf19.2024	C2_00990W_A		-2.03916
orf19.3475	C6_02330W_A		-2.03884
orf19.6586	C7_01430C_A		-2.03797
orf19.6350	C1_12720C_A		-2.03252
orf19.149	C2_04670W_A		-2.02669
orf19.634	CR_04970C_A		-2.02376
orf19.6741	C3_07470W_A		-2.01717
orf19.6073	C1_00350C_A	HMX1	-2.00982
orf19.2959.1	C1_02700C_A		-2.00243
orf19.1976	C5_00910C_A	TRX2	-1.99771
orf19.2356	CR_07060C_A	CRZ2	-1.99087
orf19.4227	C5_02200W_A		-1.98649
orf19.7384	C3_06030W_A	NOG1	-1.97998
orf19.1782.1	C2_10000C_A		-1.97964
orf19.3782	C4_04980W_A		-1.97931
orf19.5806	C2_02970C_A	ALD5	-1.97891
orf19.6475	C7_02360W_A		-1.97859
CaalfMp08	CM_00210W	COX1	-1.96798
orf19.3111	C4_06980W_A	PRA1	-1.96151
orf19.4895	C1_10250C_A		-1.95424
orf19.6021	C1_00850W_A	IHD2	-1.93874
CaalfMr16	CM_00010W	RRNL	-1.93805
orf19.849	C2_03710W_A		-1.93698
orf19.1030	C1_03790C_A		-1.92946
orf19.7422	C3_06370C_A		-1.92802
orf19.7112	C7_00100W_A	FRP2	-1.92108

orf19.7042	C7_00770W_A		-1.91903
orf19.1714	C3_01370C_A	PGA44	-1.91863
orf19.6017	C1_00880W_A		-1.9098
snR52	C3_07510C_A	SNR52	-1.90919
orf19.254	C3_02630C_A		-1.90132
orf19.6143	CR_07250C_A		-1.89568
orf19.1765	C2_10160W_A		-1.89547
orf19.5565	C6_02890C_A	HPD1	-1.89296
orf19.4526	C1_01990W_A	HSP30	-1.88898
orf19.5362	C2_10780C_A	PSO2	-1.87575
orf19.6489	C7_02240W_A	FMP45	-1.87493
orf19.2669	C4_03230C_A		-1.87394
orf19.1507	C2_01950C_A	AMN1	-1.86991
orf19.1363	C2_09880C_A		-1.85937
orf19.7300	CR_09040W_A		-1.85815
orf19.499	CR_04150W_A		-1.85763
orf19.558	CR_05220C_A	GUT1	-1.8472
orf19.169	CR_02540W_A	CHO2	-1.84346
CaalfMp05	CM_00140C	ATP9	-1.84042
orf19.3897	C5_04140W_A		-1.83996
orf19.1701	C3_01480C_A	RKI1	-1.83779
orf19.4689	C4_01000C_A	PGA57	-1.83521
orf19.9	CR_07120C_A		-1.83123
orf19.4082	C2_09220W_A	DDR48	-1.83069
orf19.3931	C5_04440C_A	SFC1	-1.82771
orf19.4674.1	C4_01160W_A	CRD2	-1.82623
orf19.3306	C1_01180C_A		-1.82612
orf19.6169	C3_00920W_A	ATO1	-1.82239
orf19.3740	CR_02280W_A	PGA23	-1.8223
orf19.6005	C3_05270C_A	HGT5	-1.80385
orf19.4749	C1_08900W_A		-1.80114
orf19.2479	C1_05710C_A	UGA4	-1.79721
orf19.5316	C2_10430C_A	FGR29	-1.79345
orf19.5926	C3_04660C_A	ARG11	-1.77352
orf19.7071	C7_00480W_A	FGR2	-1.76921
orf19.4573	C4_02180C_A	ZCF26	-1.767
orf19.3710	CR_07810W_A	YHB5	-1.76432
orf19.22	C2_06440C_A		-1.75768
orf19.4793	C1_09330W_A		-1.75755
orf19.7094	C7_00280W_A	HGT12	-1.75678
SNRNAU4	C4_06180C_A		-1.74636
orf19.1746	C2_10320C_A		-1.74175
orf19.1502	C2_01910W_A		-1.74131
orf19.6766	C3_07300W_A	NOP13	-1.73752
orf19.4189	C4_00530C_A		-1.73204
orf19.4505	C2_04470W_A	ADH3	-1.72852
orf19.6366	CR_08080W_A		-1.72713
orf19.5952	C3_04840C_A		-1.72618

orf19.6886	C2_05750W_A		-1.72298
orf19.7513	CR_00210W_A	ALK2	-1.72113
orf19.4590	C4_02030W_A	RFX2	-1.72076
orf19.5070	C1_07990C_A		-1.71953
orf19.5850	CR_05520W_A	NOC2	-1.71568
orf19.6518	C7_02010C_A		-1.70702
orf19.6315	CR_04770C_A		-1.7038
orf19.1728	C3_01230C_A		-1.70257
orf19.685.1	C6_01940W_A		-1.70237
orf19.7512	CR_00220W_A		-1.70067
orf19.7299.1	CR_09030C_A		-1.69888
orf19.4447	C1_07200W_A	YMC1	-1.69668
orf19.8	CR_07110C_A		-1.69203
orf19.2947	C1_02590C_A	SNZ1	-1.69009
orf19.2916	C4_06220C_A		-1.68904
orf19.6459	C7_02500C_A	DPP3	-1.68673
orf19.5874	C3_04310C_A		-1.68503
orf19.675	C1_11270W_A		-1.68347
orf19.2192	C2_07900W_A	GDH2	-1.68312
orf19.1397	C2_09570C_A		-1.67087
orf19.812	C2_04090W_A		-1.66949
orf19.3733	CR_02360W_A	IDP2	-1.66262
orf19.813	C2_04080W_A		-1.65728
CaalfMp06	CM_00160C	ATP6	-1.64549
orf19.7011	C7_01030C_A		-1.64521
orf19.7534	CR_00080W_A	MIS12	-1.64464
orf19.3567	C2_05360C_A	BIO32	-1.6436
orf19.4630	C4_01550C_A	CPA1	-1.63601
orf19.7107	C7_00160C_A		-1.63284
orf19.7606	CR_10310W_A		-1.63151
orf19.740	C4_05110C_A	HAP41	-1.62555
orf19.5958	C3_04890W_A	CDR2	-1.62372
orf19.276	C3_02850C_A		-1.62345
orf19.5984	C3_05110W_A		-1.60913
orf19.2701	C4_02930W_A		-1.60677
orf19.2991	C1_03000W_A	HOL1	-1.59758
orf19.4450	C1_07170C_A	ZCF23	-1.5939
orf19.257	C3_02650W_A		-1.59368
orf19.5138	C7_03170W_A	IFA21	-1.59294
orf19.6000	C3_05220W_A	CDR1	-1.58709
orf19.5255	C1_12100C_A	PXA2	-1.58649
orf19.4096	C2_09350W_A	TAZ1	-1.58575
orf19.4011	C5_05180W_A		-1.58211
orf19.5655	C4_00290C_A		-1.58149
orf19.4697	C4_00970C_A	MDN1	-1.56992
orf19.7350	C3_05710W_A	RCT1	-1.56786
orf19.5140	C7_03150W_A		-1.56489
orf19.2922	C4_06150C_A		-1.5642

orf19.494	CR_04110W_A		-1.54846
orf19.4222	C5_02160W_A	SST2	-1.54811
orf19.4445	C1_07220W_A		-1.54763
orf19.4941	C1_13140C_A	TYE7	-1.54434
orf19.2043	C2_00810C_A		-1.54389
orf19.677	C1_11240C_A	CHO1	-1.54367
orf19.638	CR_05170C_A	FDH1	-1.53912
orf19.5986	C3_05130C_A	THI4	-1.53669
orf19.6955	C3_03650W_A	HBR3	-1.53305
orf19.3015	C1_03230C_A	ARX1	-1.52912
orf19.1985	CR_07700W_A		-1.52543
orf19.2934	C1_02450C_A		-1.524
orf19.1800	C4_05440C_A		-1.52353
orf19.6828	C3_06760W_A		-1.52202
orf19.6648	CR_05660W_A	SDA1	-1.51797
orf19.5364	C2_10810W_A		-1.51615
orf19.1345	C7_03300C_A	LIP8	-1.51495
orf19.5587	C6_03050C_A		-1.51469
orf19.5614	C6_03260W_A		-1.513
orf19.2003	C2_01210C_A	HNM1	-1.51179
orf19.5626	C6_03370W_A		-1.50471
orf19.3547	C2_05160C_A		-1.5043
orf19.3736	CR_02330C_A	KAR4	-1.50373
orf19.4401	CR_03570C_A	YVH1	-1.50172
orf19.6028	C1_00780C_A	HGC1	-1.50119
orf19.5069	C1_07980C_A		-1.49824
orf19.6413	CR_08440W_A		-1.49552
orf19.5720	C6_03540W_A		-1.49492
orf19.4274	C5_02600W_A	PUT1	-1.49478
orf19.1124	C5_03710C_A		-1.49095
orf19.3621	C2_08580W_A		-1.49082
orf19.501	CR_04170W_A		-1.48404
orf19.1340	C7_03350C_A		-1.48027
orf19.6968	C3_05320W_A		-1.47985
orf19.4884	C1_10150W_A	WOR1	-1.47945
orf19.2906	C4_06310C_A	PGA41	-1.47549
orf19.3908	C5_04250W_A	MRV8	-1.47282
orf19.3974	C5_04880C_A	PUT2	-1.47267
orf19.5049	C1_07790C_A		-1.47213
orf19.6522	C7_01990C_A		-1.47134
orf19.2910	C4_06260W_A	PGA43	-1.4707
orf19.2515	C3_01130C_A		-1.46852
orf19.3001	C1_03080C_A	TEM1	-1.46798
orf19.5010	C1_13730C_A	DIM1	-1.46741
CaalfMp11	CM_00330W	COB	-1.46343
orf19.1639	C3_02070C_A		-1.46295
orf19.7062	C7_00570W_A	RPA135	-1.46274
orf19.7013	C7_01010W_A		-1.46267

orf19.2638	C5_03430W_A		-1.45483
orf19.3638	C2_08430C_A	PGA46	-1.45238
orf19.2734	C4_02660W_A		-1.4513
orf19.568	C5_00700C_A	SPE2	-1.44681
orf19.7420	C3_06350W_A		-1.44652
orf19.6066	C1_00410C_A		-1.44035
orf19.5100	C1_08210C_A	MLT1	-1.43986
orf19.853	C2_03670W_A	SAP99	-1.43577
orf19.4335	C5_03060C_A	TNA1	-1.43441
orf19.344	C3_03460C_A		-1.43389
orf19.3439	C6_01450C_A		-1.43378
orf19.4804	C1_09420W_A	LIP2	-1.4329
orf19.4743	C1_08850C_A	AFG1	-1.42835
orf19.4880	C1_10110W_A		-1.42833
orf19.6656	C5_03480C_A		-1.42527
orf19.3923	C5_04370C_A	PGA37	-1.42132
orf19.2362	CR_07030C_A		-1.42129
orf19.1264	C4_05780C_A	CFL2	-1.42119
orf19.6417	CR_08490W_A	TSR1	-1.42028
orf19.2747	C4_02540W_A	RGT1	-1.41966
orf19.1780	C2_10020C_A		-1.41791
orf19.3777	C4_05020W_A	IMG2	-1.41588
orf19.4311	C5_02890W_A	YNK1	-1.41324
orf19.2185	C2_07960C_A	NSA1	-1.41116
orf19.1124.2	C5_03700C_A		-1.41098
orf19.7209	C1_14020W_A		-1.41085
orf19.4070	C2_09120C_A		-1.41071
orf19.1966	C5_01060C_A	BUD23	-1.40978
orf19.2899	C4_06360C_A		-1.40949
orf19.570	C5_00710W_A	IFF8	-1.40656
orf19.4093	C2_09320C_A	PES1	-1.40457
orf19.6530	C7_01910C_A		-1.40248
orf19.5305	C4_04050C_A	RHD3	-1.40119
orf19.5097	C1_08190C_A	CAT8	-1.39925
orf19.4765	C1_09080C_A	PGA6	-1.39781
orf19.6704	C7_03470W_A		-1.39686
orf19.2317	C1_10980W_A		-1.39671
orf19.3672	C1_02150W_A	GAL10	-1.39469
orf19.6675	C5_03630C_A		-1.39379
orf19.4515	C2_04390W_A		-1.39256
orf19.138	C6_01310W_A	FIG1	-1.39229
orf19.4923	C1_12930C_A		-1.39131
orf19.4688	C4_01010C_A	DAG7	-1.39033
orf19.2306	C1_11100W_A		-1.38831
orf19.2211	C2_07710W_A		-1.38725
orf19.3684	C1_02270C_A		-1.38458
RPR1	C2_08055W_A	RPR1	-1.3828
orf19.2314	C1_11000C_A		-1.38271

orf19.4908	C1_10370W_A		-1.38104
orf19.7657	CR_10690W_A	POP3	-1.38042
orf19.6873.1	C2_05620W_A	KTI11	-1.37781
orf19.1327	C4_03520C_A	RBT1	-1.37624
orf19.4668	C4_01220C_A		-1.37498
orf19.4813	C1_09490C_A	GUA1	-1.37396
orf19.4239	C5_02310C_A		-1.36616
orf19.2575	CR_01780W_A		-1.36596
orf19.3406	C6_01780C_A		-1.36439
orf19.2810	C3_03990C_A	AAP1	-1.36148
orf19.4669	C4_01200C_A	AAT22	-1.36105
orf19.1927	C5_01390C_A	SNM1	-1.36022
orf19.6113	C1_00030C_A		-1.35992
orf19.1517	C2_02030W_A	ARO3	-1.3584
orf19.4593	C4_02000C_A	RGA2	-1.35736
orf19.1342	C7_03330C_A	SHM1	-1.35393
orf19.113	C6_01070C_A	CIP1	-1.35305
orf19.6601	CR_09610C_A		-1.34907
orf19.4097	C2_09360W_A		-1.34593
orf19.7645	CR_10620C_A		-1.34576
orf19.5468	C3_00050C_A		-1.34388
orf19.7500	CR_00330C_A	PXA1	-1.34355
orf19.6536	C7_01840W_A	IQG1	-1.3434
orf19.4121	C2_05990C_A		-1.33923
orf19.2962	C1_02730W_A		-1.33688
orf19.1735	CR_04710W_A		-1.33249
orf19.1847	CR_06860C_A	ARO10	-1.33139
orf19.7381	C3_06000W_A	AHR1	-1.33122
orf19.2599	CR_01980C_A	CRC1	-1.32838
orf19.3354	C1_01640W_A	RPS42	-1.32836
orf19.2168	C2_08170W_A		-1.32663
orf19.5508	C7_03690W_A		-1.32615
orf19.3675	C1_02180W_A	GAL7	-1.32565
orf19.1646	C3_02020W_A		-1.325
orf19.2623	CR_07530C_A	ECM22	-1.32466
orf19.2489	C1_05630C_A		-1.32447
orf19.6418	CR_08500W_A		-1.31922
orf19.4760	C1_09040C_A		-1.31793
orf19.4324	C5_02990W_A		-1.31791
orf19.7456	C3_06670C_A		-1.31163
orf19.7364	C3_05840W_A		-1.3113
orf19.5311	C4_04010W_A		-1.31076
orf19.4122	C2_05980C_A		-1.3076
orf19.1258	C4_05730W_A		-1.30362
orf19.419	C1_05440C_A		-1.30027
orf19.5019	C1_13810W_A		-1.29999
orf19.7610	CR_10340W_A	PTP3	-1.2979
orf19.2948	C1_02600W_A	SNO1	-1.29326

orf19.4870	C1_10030W_A	DBP3	-1.2904
orf19.2957	C1_02670C_A		-1.28888
orf19.5921	C3_04630W_A		-1.28882
orf19.711	CR_06550C_A		-1.28711
orf19.4182	C4_00610W_A		-1.28448
orf19.1578	C2_02540W_A		-1.28438
orf19.3478	C6_02360W_A	NIP7	-1.28327
orf19.1830	C1_10580C_A		-1.28247
orf19.639	CR_05160C_A		-1.28133
orf19.5469	C3_00030C_A		-1.28088
orf19.1349	C2_08340C_A		-1.27836
orf19.3986	C5_04970C_A	PPR1	-1.27608
orf19.4159	C4_00820W_A		-1.27562
orf19.4815	C1_09510W_A	YTM1	-1.27385
orf19.2385	CR_03370C_A	KTI12	-1.27292
orf19.5188	C7_02770W_A	CHS1	-1.26569
orf19.3670	C1_02130C_A	GAL1	-1.26529
snR87	C1_13040C_A		-1.26385
orf19.4160	C4_00810C_A		-1.26239
orf19.2936	C1_02470W_A		-1.26121
orf19.6916	C7_01350C_A		-1.2597
orf19.6793	C3_07070C_A		-1.25572
orf19.467	CR_03890W_A	WOR3	-1.25399
orf19.1624.1	C3_02200W_A		-1.24935
orf19.3627	C2_08510W_A		-1.24868
orf19.2451	C1_05960W_A	PGA45	-1.24805
orf19.1529	C2_02130C_A		-1.24635
orf19.7056	C7_00630C_A		-1.244
orf19.5238	C1_12250C_A		-1.24334
orf19.962	C5_00310C_A		-1.24299
orf19.3978	C5_04910W_A		-1.24296
orf19.6192	C3_07980C_A		-1.24156
orf19.5879	C3_04350C_A		-1.24124
orf19.1596	C4_03910W_A	FGR28	-1.24032
orf19.3962	C5_04750C_A	HAS1	-1.23968
orf19.2905	C4_06320C_A		-1.23911
orf19.2137	C6_04510C_A		-1.23887
orf19.871	C2_03500W_A		-1.23808
orf19.3540	C2_05090W_A	MAK5	-1.2345
orf19.7296	CR_08990C_A		-1.23439
orf19.4504	C2_04480W_A		-1.23344
orf19.3708	CR_07800W_A	SAP2	-1.2331
orf19.6686	C7_03540C_A	ENP2	-1.2286
orf19.2748	C4_02530W_A	ARG83	-1.22356
orf19.1939	C5_01300C_A		-1.22186
orf19.4072	C2_09130C_A	IFF6	-1.22185
orf19.2917	C4_06210C_A		-1.21952
orf19.2033	C2_00910W_A	PGA19	-1.21854

orf19.4349	C5_03170C_A		-1.21741
orf19.5748	C6_03740W_A		-1.20752
orf19.1704	C3_01460C_A	FOX3	-1.20719
orf19.5514	C6_02450W_A		-1.20079
orf19.5762	C6_03860C_A	PGA61	-1.20057
orf19.4456	C1_07120W_A	GAP4	-1.20022
orf19.7105	C7_00180W_A	FAR1	-1.19994
orf19.6551.1	C7_01730C_A	STE18	-1.19992
orf19.7664	CR_10750C_A		-1.19905
orf19.2753	C4_02490W_A	ZCF15	-1.19029
orf19.4055	C5_05510C_A		-1.18815
orf19.2284	C2_07270W_A		-1.18772
orf19.6652	CR_05630W_A	DBP8	-1.18746
orf19.1566	C2_02430W_A	UTP21	-1.18651
orf19.3676	C1_02190W_A	ABP140	-1.18327
orf19.6124	CR_07440W_A	ACE2	-1.1822
orf19.2998	C1_03060C_A	TSR2	-1.18186
orf19.3793	C4_04860W_A		-1.18057
orf19.5343	C2_10660W_A	ASH1	-1.17879
orf19.2250	C2_06960W_A	SPE3	-1.17841
orf19.3852	CR_06030C_A		-1.17803
orf19.512	CR_04240C_A		-1.17756
orf19.4587	C4_02050W_A	HGH1	-1.17386
orf19.3669	C1_02120C_A	SHA3	-1.17375
orf19.5216	C2_05960C_A		-1.17295
orf19.5020	C1_13820C_A		-1.17285
orf19.6148	CR_07220C_A		-1.17158
orf19.2898	C4_06370C_A		-1.1712
orf19.3159	C3_01200W_A	UTP20	-1.16853
orf19.2783	C1_07620C_A	PIR32	-1.16839
orf19.7635	CR_10550W_A	DRS1	-1.16521
orf19.2005	C2_01200C_A	REG1	-1.15861
orf19.4479	C1_04040C_A		-1.1578
orf19.984	C1_10430W_A	PHO8	-1.15634
orf19.2848	CR_02910W_A		-1.15591
orf19.6781	C3_07200C_A	ZFU2	-1.15548
orf19.2227	C2_06760C_A		-1.15327
orf19.857	C2_03610W_A	FMO2	-1.15236
orf19.4896	C1_10260C_A	RPA34	-1.1498
orf19.5293	C4_04160W_A		-1.14951
orf19.5550	C6_02770W_A	MRT4	-1.14944
orf19.6282	CR_07680C_A		-1.14673
orf19.7219	C1_14130W_A	FTR1	-1.14618
orf19.6242	C1_06690W_A	CYK3	-1.14609
orf19.7400	C3_06320W_A	ALS7	-1.14361
orf19.6474	C7_02370W_A		-1.14303
orf19.6869	C2_05570C_A		-1.1421
orf19.2712	C4_02830C_A	HCA4	-1.14131

orf19.4451	C1_07140C_A	RIA1	-1.14108
orf19.360	C3_03610W_A	FUR4	-1.14075
orf19.6723	C3_07650C_A		-1.14049
orf19.3600	C2_08750W_A		-1.13999
orf19.6175	C3_07800C_A		-1.13971
orf19.2367	CR_06980W_A		-1.13488
orf19.270	C3_02790W_A		-1.13444
orf19.1783	C2_09990C_A	YOR1	-1.13396
orf19.4996	C1_13600W_A		-1.13049
orf19.4191	C4_00510C_A	RLP24	-1.12987
orf19.6141	CR_07270C_A	HGT16	-1.12969
orf19.3564	C2_05340C_A	RPC40	-1.12929
orf19.2312	C1_11020W_A		-1.12927
orf19.1522	C2_02050C_A		-1.12892
orf19.313	C3_03140C_A	DAL4	-1.12851
orf19.706	CR_06720W_A	NMD3	-1.12792
orf19.4243	C5_02350C_A		-1.12778
orf19.5991	C3_05160C_A		-1.12751
orf19.2951	C1_02620C_A	HOM6	-1.12557
orf19.782	C1_04640W_A		-1.12478
orf19.810	C2_04110W_A		-1.12339
orf19.6420	CR_08510W_A	PGA13	-1.12287
orf19.1090	C6_04320C_A		-1.12175
orf19.2808	C3_04020C_A	ZCF16	-1.12113
orf19.1789.1	C4_05320W_A	LYS1	-1.11945
orf19.473	CR_03920C_A	TPO4	-1.11713
orf19.4591	C4_02020W_A	CAT2	-1.11691
orf19.4538	C1_01850C_A		-1.11632
orf19.2480.1	C1_05700W_A	AUT7	-1.11416
orf19.7088	C7_00330C_A		-1.11317
orf19.1915	C2_00070C_A	MPP10	-1.1131
orf19.7154	C7_04190C_A	UTP18	-1.11224
orf19.1647	C3_02010C_A		-1.1112
orf19.809	C2_04120C_A		-1.10982
orf19.2685	C4_03070W_A	PGA54	-1.10952
orf19.862	C2_03570C_A		-1.10861
orf19.4017	C5_05240C_A		-1.10522
orf19.1377	C2_09730C_A	IPK2	-1.10341
orf19.4035	C5_05390C_A	PGA4	-1.10326
orf19.2209	C2_07730W_A	YVC1	-1.10114
orf19.5094	C1_08170C_A	BUL1	-1.09822
orf19.5824	C2_02820C_A		-1.09424
orf19.1051	C1_04170C_A	HTA2	-1.09231
orf19.3539	C2_05080C_A		-1.09153
orf19.6902	C7_01200C_A	DBP7	-1.09078
orf19.4244	C5_02360C_A		-1.09065
orf19.5356	C2_10740C_A		-1.09019
orf19.1826	C1_06230C_A	MDM34	-1.08993

orf19.7517	CR_00180C_A	CHT1	-1.08608
orf19.773	C1_04710C_A		-1.08571
orf19.278	C3_02870C_A		-1.08557
orf19.5249	C1_12150C_A		-1.08128
orf19.3982	C5_04940W_A		-1.07422
orf19.3066	C1_03680W_A	ENG1	-1.06979
orf19.4640	C4_01450W_A	PWP1	-1.06857
orf19.3276	CR_00800C_A	PWP2	-1.06757
orf19.500	CR_04160C_A		-1.06675
orf19.3668	C1_02110C_A	HGT2	-1.06624
orf19.4655	C4_01320C_A	OPT6	-1.06429
orf19.604	CR_07940W_A		-1.06354
orf19.2745	C4_02560C_A	UME7	-1.06244
orf19.3291	C1_01030W_A	HMT1	-1.0621
orf19.3138	C4_06720W_A	NOP1	-1.06091
orf19.3499	C6_02100W_A		-1.05971
orf19.930	C5_00590W_A	PET9	-1.05965
orf19.5610	C6_03230W_A	ARG3	-1.05892
orf19.334	C3_03370C_A		-1.05818
orf19.1651	C3_01970C_A		-1.05778
orf19.6297	C5_01610W_A		-1.05699
orf19.2859	CR_02980C_A	SRP40	-1.0569
orf19.7160	C7_04140C_A		-1.05673
orf19.695	CR_06590C_A	RGS2	-1.05562
orf19.3840	C4_04460C_A		-1.05457
orf19.2082	C2_00460W_A	SAP30	-1.05256
orf19.4400	CR_03560W_A		-1.05239
orf19.2646	C5_03330C_A	ZCF13	-1.05227
orf19.2042	C2_00820W_A		-1.05213
orf19.3460	C6_02200C_A		-1.05112
orf19.2246	C2_06920C_A		-1.05062
orf19.68.2	C1_12480W_A		-1.05014
orf19.5012	C1_13750C_A		-1.04683
orf19.7552	CR_09800C_A		-1.0466
orf19.4805	C1_09430W_A		-1.04658
orf19.4195	C6_00630W_A		-1.04602
orf19.4492	C2_04570W_A		-1.04209
orf19.2504	C3_01000W_A	BMS1	-1.04067
orf19.3434	C6_01500C_A	TRY5	-1.03872
orf19.5500	C2_06360C_A	MAK16	-1.03871
orf19.938	C5_00510W_A		-1.03863
orf19.1633	C3_02130W_A	UTP4	-1.03755
orf19.1150	C1_11690W_A		-1.03736
orf19.124	C6_01170W_A	CIC1	-1.03608
orf19.3037	C1_03370W_A		-1.0358
orf19.1091	C6_04330W_A	NOP8	-1.03369
orf19.2229	C2_06780C_A		-1.03348
orf19.5987	C3_05140C_A		-1.03254

orf19.5639	C4_00140C_A	HIS4	-1.0318
orf19.35.1	C2_06610C_A		-1.03086
orf19.7472	CR_00610W_A	IFF4	-1.03062
orf19.6971	C3_05340W_A	DIE2	-1.03041
orf19.1082	C6_04240W_A		-1.02901
orf19.1117	C5_03770C_A		-1.02801
orf19.7305	CR_09090C_A		-1.02754
orf19.1747	C2_10310C_A	KIP2	-1.0271
orf19.2115	C2_00170C_A		-1.02662
orf19.1793	C4_05350W_A		-1.02403
orf19.341	C3_03440C_A		-1.02175
orf19.7569	CR_09950C_A	SIK1	-1.02168
orf19.3912	C5_04280C_A	GLN3	-1.02122
orf19.3425	C6_01620W_A		-1.02062
orf19.5714	C6_03490C_A	SAP1	-1.02002
orf19.2059	C2_00690W_A		-1.01908
orf19.2191	C2_07920W_A		-1.01835
orf19.4459	C1_03870C_A		-1.01691
orf19.3751	CR_02210W_A		-1.01572
orf19.665	C1_11380W_A	NEP1	-1.01559
orf19.6168	C3_00910W_A		-1.01393
orf19.7565	CR_09910W_A	GNP3	-1.01321
orf19.5976	C3_05060W_A		-1.01242
orf19.6713	C3_07730W_A	WOR4	-1.0114
orf19.5753	C6_03790C_A	HGT10	-1.01048
orf19.7006	C7_01070C_A		-1.00832
orf19.2547	CR_01600C_A		-1.00399
orf19.6678	C5_03650C_A		-1.00297
orf19.3255	CR_01010W_A	TEN1	-1.00209
orf19.596.1	CR_08020C_A	NOP10	-1.0014
orf19.6234	C1_06760C_A		-1.00051
orf19.1202	C6_00400C_A		-1.00044
orf19.3108	C4_07010C_A		-0.999946
orf19.5912	C3_04560W_A	MAK21	-0.99916
orf19.3419	C6_01670W_A	MAE1	-0.999085
orf19.7223	C1_14160W_A		-0.996328
orf19.5959	C3_04900W_A	NOP14	-0.993695
orf19.6236	C1_06740C_A	NOP6	-0.991997
orf19.4779	C1_09210C_A		-0.991063
orf19.2674	C4_03160C_A		-0.989605
orf19.2752	C4_02500C_A	ADR1	-0.988841
orf19.3471	C6_02300C_A		-0.987758
orf19.1505	C2_01930C_A		-0.986753
orf19.3893	C5_04110W_A	SCW11	-0.984389
orf19.4273	C5_02590C_A		-0.983279
orf19.2320	C1_10950C_A		-0.98231
orf19.3526	C2_04940C_A	ITR1	-0.982288
orf19.4662	C4_01260W_A	RLM1	-0.98025

orf19.3221	CR_01330W_A	CPA2	-0.980162
orf19.6830	C3_06730W_A		-0.977147
orf19.4270	C5_02570W_A	MNN13	-0.977079
orf19.661	C1_11420W_A	KRR1	-0.976561
orf19.4393	CR_03500W_A	CIT1	-0.972214
orf19.7298	CR_09020C_A	CHS2	-0.970697
orf19.310	C3_03130C_A		-0.970544
orf19.7173	C7_04060W_A		-0.969818
orf19.7502	CR_00310C_A		-0.969039
orf19.6605	CR_09570W_A		-0.968693
orf19.2342	C1_10770W_A	SFT2	-0.967901
orf19.3742	CR_02270C_A		-0.967873
orf19.2230	C2_06790W_A		-0.967457
orf19.2075	C2_00520W_A	DFG5	-0.965994
orf19.3267	CR_00870C_A		-0.962919
orf19.1569	C2_02450C_A	UTP22	-0.962866
orf19.7650	CR_10650W_A	LTV1	-0.962434
orf19.6048	C1_00580W_A		-0.961667
orf19.2170	C2_08140C_A	PHM7	-0.961262
orf19.1938	C5_01310W_A		-0.96114
orf19.2117	C2_00150W_A	LEU5	-0.959266
orf19.1449	C2_01450C_A		-0.9591
orf19.2668	C4_03250C_A	RHD2	-0.958809
orf19.76	C6_04160C_A	SPB1	-0.95875
orf19.4647	C4_01390W_A	HAP3	-0.957631
orf19.4012	C5_05190W_A	PCL5	-0.957208
orf19.3512	CR_05430W_A	CSP1	-0.957056
orf19.918	C3_04070C_A	CDR11	-0.956926
orf19.3625	C2_08540C_A		-0.956813
orf19.5392	C3_00650W_A	NGT1	-0.955045
orf19.2564	CR_01710W_A		-0.954487
orf19.6007	C3_05280C_A		-0.952967
orf19.6254	C1_06570W_A	ANT1	-0.952762
orf19.850	C2_03700W_A		-0.950716
SCR1	CR_04275W_A	SCR1	-0.950029
orf19.1047	C1_04130W_A	ERB1	-0.949534
orf19.4028	C5_05330C_A	RER2	-0.94748
orf19.6947	C3_03720W_A	GTT11	-0.946738
orf19.7601	CR_10260W_A		-0.944922
orf19.2788	C1_07570C_A		-0.944159
orf19.7205	C1_13980C_A	DUR7	-0.941869
orf19.3351	C1_01610C_A		-0.9409
orf19.2723	C4_02750W_A	HIT1	-0.940249
orf19.4599	C4_01940W_A	PHO89	-0.939116
orf19.7050	C7_00690W_A	NOP15	-0.938572
orf19.6724	C3_07640C_A	FUM12	-0.937999
orf19.1393	C2_09610W_A		-0.936026
orf19.2309	C1_11070W_A	PET127	-0.935536

orf19.5566	C6_02900C_A		-0.934872
orf19.4101	C2_06160W_A		-0.933269
orf19.3311	C1_01240W_A	IFD3	-0.933001
orf19.2270	C2_07160W_A	SMF12	-0.932789
orf19.3732	CR_02370W_A	ERG25	-0.930764
orf19.3783	C4_04950W_A		-0.929765
orf19.100	C6_00980C_A		-0.929707
orf19.2717	C4_02790C_A	SAS10	-0.929379
orf19.7495	CR_00380W_A		-0.929269
orf19.4066	C2_09070C_A		-0.928658
orf19.6298	C5_01600C_A	SPB4	-0.927408
orf19.59	C1_05060W_A	REI1	-0.927156
orf19.7436.1	C3_06480C_A	ECM15	-0.925295
orf19.2517	CR_01340W_A		-0.924448
orf19.2726	C4_02720C_A		-0.924186
orf19.4411	C4_06010C_A	HOS1	-0.923996
orf19.4337	C5_03080C_A		-0.923518
orf19.1250	C4_05650W_A		-0.923284
orf19.2812	C3_03980C_A		-0.920294
orf19.2196	C2_07860W_A		-0.919743
orf19.4114	C2_06070W_A	FAA2-1	-0.919511
orf19.999	C1_10550C_A	GCA2	-0.919091
orf19.3201	C5_01740C_A	MTLA1	-0.918607
orf19.4883	C1_10140C_A		-0.918503
orf19.345	C3_03470W_A		-0.917681
orf19.1318	C4_03580W_A		-0.917637
orf19.1902	C2_07340W_A	NOC4	-0.916721
orf19.4399	CR_03550W_A	NCS2	-0.914809
orf19.1429	C4_04210C_A	SOH1	-0.911818
orf19.5949	C3_04830C_A	FAS2	-0.910784
orf19.4167	C4_00750C_A		-0.910241
orf19.4780	C1_09220W_A		-0.910032
orf19.4420	C4_06090C_A		-0.909282
orf19.346	C3_03480C_A	ALT1	-0.90693
orf19.1642	C3_02040C_A		-0.906914
orf19.7371	C3_05910W_A	ZCF35	-0.906705
orf19.676	C1_11250W_A		-0.905987
orf19.3770	C4_05070C_A	ARG8	-0.905888
orf19.5430	C3_00370C_A	BUD21	-0.90495
orf19.4450.2	C1_07150W_A		-0.904493
orf19.3851	CR_06020W_A		-0.903697
orf19.2066	C2_00620C_A		-0.903164
orf19.6953	C3_03660W_A	IRS4	-0.902944
orf19.3631	C2_08470C_A	STN1	-0.902691
orf19.6480	C7_02310C_A		-0.902481
orf19.6548	C7_01760C_A	ISU1	-0.902043
orf19.1663	C3_01830C_A	MNT2	-0.902018
orf19.7197	C7_03850W_A		-0.901496

orf19.1330	C7_03460W_A		-0.901217
orf19.3042	C1_03410W_A		-0.900997
orf19.816	C2_04040C_A	DCK2	-0.900427
orf19.5125	C7_03280C_A		-0.900078
orf19.6676	C5_03640W_A		-0.900028
orf19.81	C2_06660W_A		-0.899219
orf19.3763	C1_12530C_A		-0.897924
orf19.3298	C1_01100W_A	CCH1	-0.896928
orf19.3667	C1_02100W_A		-0.896839
orf19.3518	CR_05480W_A		-0.896798
orf19.7148	C7_04250W_A	TPO2	-0.895038
orf19.215	C2_08920W_A		-0.894227
orf19.1164	C1_11550W_A	GAR1	-0.894056
orf19.3841	C4_04450C_A	ATG1	-0.893639
orf19.6264.3	C1_06480C_A		-0.892973
orf19.552	CR_04610C_A		-0.89161
orf19.518	CR_04300W_A		-0.890433
orf19.5370	C2_10870W_A		-0.890283
orf19.526	CR_04360C_A	NHP2	-0.890107
orf19.5026	C1_13880C_A		-0.887566
orf19.1693	C3_01530C_A	CAS4	-0.88511
orf19.6689	C7_03570W_A	ARG4	-0.884974
orf19.2882	C4_06530C_A	XUT1	-0.88493
orf19.1052	C1_04180W_A		-0.884669
orf19.5735.3	C6_03670C_A		-0.884613
orf19.7566	CR_09920W_A		-0.882789
orf19.956	C5_00330C_A		-0.88277
orf19.5543	C6_02720C_A		-0.881994
orf19.6326	C6_00110C_A		-0.881207
orf19.2939	C1_02500W_A		-0.880914
orf19.1950	C5_01200W_A		-0.880698
orf19.3455	C6_02150C_A		-0.880493
orf19.5507	C7_03700C_A	ENP1	-0.879908
orf19.5567	C6_02910W_A	POP4	-0.878459
orf19.5677	C4_00480W_A	DUR4	-0.878242
orf19.6790	C3_07100C_A		-0.877779
orf19.7556	CR_09840C_A		-0.877257
orf19.3498	C6_02110W_A		-0.875732
orf19.5022	C1_13840W_A		-0.875156
orf19.1585	C2_02590W_A	ZRT2	-0.874352
orf19.4110	C2_06090W_A		-0.874135
orf19.4788	C1_09290C_A	ARG5,6	-0.874026
orf19.3854	CR_06040W_A		-0.873495
orf19.2281	C2_07240C_A		-0.872783
orf19.2724	C4_02740W_A		-0.872646
orf19.1332	C7_03440W_A	SNG4	-0.872032
orf19.7618	CR_10410C_A		-0.869003
orf19.7073	C7_00460W_A		-0.868802

orf19.4325	C5_03000C_A		-0.868323
orf19.3998	C5_05060C_A		-0.86714
orf19.5278	C1_11890W_A		-0.866918
orf19.5718	C6_03520C_A		-0.866259
orf19.1404	C2_09500W_A		-0.864073
orf19.4858	C1_09920W_A	VPS41	-0.86355
orf19.1853	CR_06810W_A	HHT2	-0.862233
orf19.4177	C4_00650W_A	HIS5	-0.862065
orf19.2198	C2_07830W_A	FLC3	-0.861064
orf19.5791	C2_03080W_A	IDH2	-0.860939
orf19.1388	C2_09660W_A		-0.860143
orf19.3088	C4_07150W_A		-0.859947
orf19.708	CR_06740W_A		-0.857379
orf19.6903	C7_01210C_A		-0.856978
orf19.5118	C1_08370W_A	SDS24	-0.856711
orf19.6306	CR_04870C_A		-0.85671
orf19.4791	C1_09310C_A		-0.856216
orf19.3309	C1_01210W_A		-0.855933
orf19.6152	CR_07200W_A		-0.853052
orf19.7063	C7_00560C_A	THG1	-0.852586
orf19.3784	C4_04940W_A		-0.852573
orf19.2143	C6_04530C_A		-0.852569
orf19.4365	CR_03660C_A		-0.852522
orf19.563	C2_09380W_A	RRP15	-0.852286
orf19.4015	C5_05220W_A	CAG1	-0.851835
orf19.206	C2_08990C_A		-0.851139
orf19.6829	C3_06740W_A		-0.850391
orf19.7379	C3_05980C_A	FAA2	-0.848942
orf19.4634	C4_01500W_A		-0.848739
orf19.2287	C2_07300C_A	RPA12	-0.848305
orf19.4885	C1_10160W_A	MIR1	-0.845537
orf19.1352	C2_08310W_A	TIM22	-0.844659
orf19.300	C3_03040W_A	AIP2	-0.844545
orf19.4931	C1_13030C_A		-0.843069
orf19.6163	C3_00860W_A	CSE4	-0.843063
orf19.4771	C1_09130W_A		-0.841065
orf19.4143	C5_01480W_A	FYV5	-0.840393
orf19.7159	C7_04150W_A		-0.839343
orf19.7342	CR_09470W_A	AXL1	-0.838244
orf19.3791	C4_04880W_A	FGR10	-0.838141
orf19.1839	C1_10670C_A	RPA190	-0.836478
orf19.2990	C1_02990C_A	XOG1	-0.834532
orf19.1813	CR_07100W_A	FLC2	-0.834507
orf19.5147	C7_03080W_A	LMO1	-0.834268
orf19.6062.3	C1_00450C_A		-0.833632
orf19.3863	CR_06100C_A		-0.833232
orf19.2829	CR_02700W_A		-0.832996
orf19.3569	C2_05380W_A		-0.83267

orf19.7010	C7_01040C_A		-0.831207
orf19.4919	C1_12890W_A		-0.831132
orf19.3275	CR_00810W_A		-0.830626
orf19.3435	C6_01490C_A		-0.82988
orf19.7594	CR_10180W_A		-0.829012
orf19.4746	C1_08870C_A	JIP5	-0.829003
orf19.3690.2	C1_02330C_A		-0.828554
orf19.578	C5_00760W_A		-0.827685
orf19.2527	CR_01410C_A		-0.826856
orf19.3553	C2_05230C_A	RPF2	-0.82611
orf19.1676	C3_01680C_A		-0.825066
orf19.5050	C1_07800W_A	MTO1	-0.824118
orf19.6773	C3_07260C_A	ECM29	-0.823699
orf19.4476	C1_04010C_A		-0.822013
orf19.6501	C7_02140W_A		-0.821589
orf19.7104	C7_00190W_A		-0.820853
orf19.5038	C4_03830W_A		-0.818559
orf19.342	C3_03450C_A	BMT7	-0.817931
orf19.6276	C1_06350W_A		-0.816828
orf19.5505	C7_03720C_A	HIS7	-0.816723
orf19.4465	C1_03910C_A		-0.81623
orf19.1420	C4_04280C_A		-0.816076
orf19.6494	C7_02200W_A	WHI3	-0.816037
orf19.2580	CR_01800C_A	HST2	-0.815785
orf19.1351	C2_08320C_A		-0.815131
orf19.4135	C5_01450W_A	PRC2	-0.814992
orf19.1618	C3_02280C_A	GFA1	-0.814618
orf19.1369	C2_09810C_A		-0.814526
orf19.5163	C7_02980C_A	SFI1	-0.814088
orf19.2067	C2_00600C_A		-0.813114
orf19.3516	CR_05450C_A		-0.812016
orf19.4029	C5_05340W_A		-0.811944
orf19.6378	CR_08180C_A	TRM9	-0.811904
orf19.603	CR_07950W_A	IMP4	-0.810751
snR128	C1_08970W_A		-0.810226
orf19.6014	C1_00900W_A	RRS1	-0.80986
orf19.2711	C4_02850W_A		-0.809804
orf19.2157	C6_04600W_A	DAC1	-0.808143
orf19.6532	C7_01880C_A		-0.807548
orf19.1727	C3_01250W_A	PMC1	-0.805727
orf19.2182	C2_08010W_A	BLM3	-0.803894
orf19.1955	C5_01170W_A		-0.8036
orf19.6958	C3_03630W_A	ECM18	-0.802296
orf19.2709	C4_02870C_A	ZUO1	-0.801921
orf19.541	CR_04500C_A		-0.801516
orf19.3356	C1_01670C_A	ESP1	-0.801379
orf19.2521	CR_01380W_A		-0.800353
orf19.5051	C1_07810C_A		-0.797213

orf19.6779	C3_07220C_A	PRO2	-0.797081
orf19.1609	C3_02350W_A		-0.795685
orf19.2785	C1_07600W_A	ATP7	-0.795298
orf19.2386	CR_03360W_A		-0.793709
orf19.3778	C4_05010W_A		-0.79362
orf19.1059	C1_04240C_A	HHF1	-0.792451
orf19.6643	CR_05690W_A	MCT1	-0.792397
orf19.2908	C4_06280C_A		-0.791593
orf19.2638.1	C5_03440W_A		-0.791395
orf19.3606	C2_08700C_A		-0.79124
orf19.7668	CR_10790W_A	MAL2	-0.79071
orf19.588	C5_00850C_A		-0.790574
orf19.2397	CR_03270W_A		-0.789066
orf19.172	CR_02520W_A	RPC19	-0.788804
orf19.2237.1	C2_06850W_A		-0.787848
orf19.916	CR_06560C_A		-0.785188
orf19.318	C3_03190C_A		-0.784684
orf19.2090	C2_00410C_A		-0.781332
orf19.7634	CR_10540C_A	MCD1	-0.780853
orf19.1886	C2_07450C_A	RCL1	-0.779924
orf19.2360	CR_07050C_A	URA2	-0.779814
orf19.4418	C4_06070C_A	FMT1	-0.778086
orf19.4795	C1_09340C_A		-0.776977
orf19.2495	C1_05600W_A	GSL1	-0.776918
orf19.2736	C4_02630C_A	HFL2	-0.774628
orf19.2064	C2_00640W_A		-0.772709
orf19.28	C2_06520C_A		-0.772337
orf19.6972	C3_05350C_A	SMI1B	-0.770236
orf19.1244	C4_05600W_A	GYP2	-0.769593
orf19.7370	C3_05900W_A		-0.769005
orf19.2594	CR_01950W_A		-0.768767
orf19.1855	CR_06790C_A		-0.767545
orf19.1223	C2_06670C_A	DBF2	-0.766938
orf19.1406	C2_09480W_A		-0.76673
orf19.2631	C4_05260W_A		-0.766572
orf19.6805	C3_06950W_A		-0.765125
orf19.423	C1_05420W_A		-0.764526
orf19.6277	C1_06340W_A		-0.763051
orf19.4705	C4_00880W_A		-0.761875
orf19.1335	C7_03400C_A		-0.759753
orf19.4432	C1_07380C_A	KSP1	-0.759074
orf19.2444	C1_06010W_A	CHS7	-0.759045
orf19.3601	C2_08740W_A		-0.757343
orf19.5719	C6_03530C_A		-0.75693
orf19.6240	C1_06700W_A		-0.756898
orf19.3470	C6_02290C_A		-0.756836
orf19.2921	C4_06160W_A		-0.756784
orf19.2133	C6_04490W_A	LIP4	-0.755969

orf19.4404	C4_05920C_A	PGA49	-0.754504
orf19.2657	C5_03210C_A		-0.754296
orf19.2980	C1_02900C_A		-0.753855
orf19.2956	C1_02660C_A	MGM101	-0.753701
orf19.4657	C4_01300W_A		-0.752512
orf19.4059	C1_05120W_A		-0.752022
orf19.1495	C2_01860C_A		-0.750182
orf19.600	CR_07960C_A	TRK1	-0.749084
orf19.1515	C2_02010C_A	CHT4	-0.748874
orf19.5752	C6_03780C_A		-0.748808
orf19.6146	CR_07240C_A	CLG1	-0.748798
orf19.2506	C3_01020W_A		-0.748585
orf19.7158	C7_04160W_A		-0.748569
orf19.5244	C1_12200W_A	MCD4	-0.748144
orf19.7637	CR_10570C_A	YHB4	-0.747632
orf19.1708	C3_01430W_A		-0.745087
orf19.896	C2_03320W_A	CHK1	-0.744697
orf19.4363	CR_03650W_A	SGD1	-0.744084
orf19.3629	C2_08490W_A	DSE1	-0.743429
orf19.3682	C1_02250W_A	CWH8	-0.743225
orf19.5432	C3_00350W_A	TPT1	-0.743129
orf19.3266	CR_00880W_A		-0.742299
orf19.3305	C1_01170C_A	ZCF17	-0.740281
orf19.1753	C2_10270W_A	PUS7	-0.738942
orf19.5797	C2_03040W_A	PLC2	-0.738821
orf19.2017	C2_01070W_A		-0.736849
orf19.517	CR_04290W_A	HAP31	-0.734934
orf19.875	C2_03450W_A		-0.73414
orf19.2030	C2_00940W_A		-0.733051
orf19.6629	CR_05830C_A	ISC1	-0.732645
orf19.7100	C7_00230W_A		-0.73188
orf19.4409	C4_05980C_A		-0.731163
orf19.1288	C3_00810C_A	FOX2	-0.727123
orf19.2697	C4_02960W_A		-0.726437
orf19.3630	C2_08480W_A	RRP8	-0.72448
orf19.320	C3_03210W_A		-0.723753
orf19.7339	CR_09420C_A	BGL22	-0.723142
orf19.4677	C4_01130C_A		-0.722904
orf19.5884	C3_04370C_A		-0.722355
orf19.2831	CR_02720C_A	RPC31	-0.722319
orf19.3788	C4_04910C_A	SPC34	-0.720812
orf19.3894	C5_04120C_A		-0.720426
orf19.477	CR_03950W_A		-0.718992
orf19.6283	CR_07670W_A		-0.71863
orf19.6118	CR_07470W_A		-0.717595
orf19.6943	C3_03740W_A		-0.717187
orf19.5660	C4_00320C_A		-0.71522
orf19.7545	CR_00010C_A		-0.715138

orf19.5885	C3_04380C_A		-0.714781
orf19.3142	C4_06670W_A		-0.714298
orf19.1969	C5_01010W_A	CCW14	-0.714044
orf19.1394	C2_09600C_A		-0.713904
orf19.405	C1_08580C_A	VCX1	-0.711867
orf19.1296	C2_06650C_A		-0.709668
orf19.474	CR_03930C_A		-0.707835
orf19.4250	C5_02410C_A		-0.706224
orf19.5337	C2_10580W_A	UBC15	-0.706112
orf19.6119	CR_07460C_A		-0.706052
orf19.1687	C3_01560W_A		-0.7057
orf19.7255	C1_14390W_A	RPC10	-0.70369
orf19.5848	CR_05540C_A		-0.703333
orf19.1575	C2_02510W_A	PRS1	-0.702561
orf19.5365	C2_10820C_A		-0.702377
orf19.5137.1	C7_03180C_A	HHO1	-0.701915
orf19.6310	CR_04830C_A		-0.701283
orf19.7387	C3_06060W_A	ELP3	-0.701178
orf19.7291	CR_08940W_A		-0.699859
orf19.6578	C7_01510W_A		-0.69861
orf19.6941	C3_03760W_A		-0.698547
orf19.6979	C3_05410W_A		-0.698447
orf19.5384	C3_00710W_A	CHS8	-0.696636
orf19.6376	CR_08160W_A	PTC5	-0.692711
orf19.1490	C2_01780W_A	MSB2	-0.692052
orf19.7469	CR_00620C_A	ARG1	-0.691165
orf19.1499	C2_01890W_A	CTF1	-0.690717
orf19.6272	C1_06390W_A		-0.690301
orf19.6563	C7_01620C_A	KCH1	-0.68546
orf19.3555	C2_05260W_A	BUD14	-0.684403
orf19.6630	CR_05800C_A		-0.68327
orf19.3586	C2_08830W_A		-0.683078
orf19.4376	CR_03780C_A		-0.6825
orf19.1584	C2_02580W_A		-0.682196
orf19.5412	C3_00510W_A		-0.679143
orf19.3102	C4_07070W_A	CTA6	-0.678863
orf19.93	C6_00920W_A		-0.674995
orf19.6414	CR_08450C_A		-0.672979
orf19.1301	C4_03770W_A		-0.672945
orf19.7593	CR_10170C_A		-0.670233
orf19.4412	C4_06020C_A		-0.669948
orf19.2256	C2_07000W_A		-0.668843
orf19.2769	C4_02340W_A		-0.668811
orf19.3836	C4_04500C_A		-0.667754
orf19.3773	C4_05060W_A	CDL1	-0.664676
orf19.7401	C3_06310C_A	ISW2	-0.65991
orf19.6286	CR_07640C_A		-0.658456
orf19.649	CR_05030W_A		-0.656295

orf19.5110	C1_08310W_A	OPY2	-0.653683
orf19.6173	C3_07790W_A	STD1	-0.644325
orf19.6653	CR_05620C_A	MTG2	-0.636624
orf19.2760	C4_02430W_A		0.635655
orf19.3051	C1_03490W_A		0.644419
orf19.5572	C6_02940C_A		0.649072
orf19.2455	C1_05930C_A		0.649996
orf19.7579	CR_10030W_A	FGR34	0.652074
orf19.227	C3_02400C_A	COX7	0.656467
orf19.151	C2_04690C_A	TPO5	0.656731
orf19.156	C2_04720C_A	FGR51	0.659643
orf19.1143	C1_11740W_A		0.659936
orf19.1192	C6_00320C_A	DNA2	0.65999
orf19.2414	CR_03120W_A		0.660861
orf19.3195	C5_01800C_A	HIP1	0.66259
orf19.3944	C5_04600C_A	GRR1	0.663565
orf19.6348	C1_12740W_A		0.665513
orf19.5196	C1_04410C_A	ESS1	0.666324
orf19.3649	C6_00760W_A		0.670297
orf19.686	C6_01950C_A		0.675812
orf19.6393	CR_08290W_A		0.676024
orf19.1115	C5_03790W_A	GUK1	0.676476
orf19.4723	C1_08650C_A	FAD1	0.676926
orf19.3148	C2_06740W_A		0.682357
orf19.3043	C1_03430W_A		0.68279
orf19.7083	C7_00370W_A	DCC1	0.682933
orf19.6668	C5_03570W_A		0.684438
orf19.3228	CR_01260W_A		0.6852
orf19.5815	C2_02890W_A	SCT2	0.685997
orf19.5393	C3_00640W_A		0.686494
orf19.5855	C3_04110C_A	MBP1	0.689515
orf19.5379	C3_00760W_A	ERG4	0.690642
orf19.5727	C6_03590C_A		0.690679
orf19.2174	C2_08110W_A	RAD57	0.690777
orf19.5411	C3_00520W_A		0.691262
orf19.3581	C2_05510C_A		0.692176
orf19.2063	C2_00650W_A		0.694846
orf19.4759	C1_09030C_A	COX5	0.695019
orf19.752	C1_04910C_A		0.696709
orf19.3752	CR_02200C_A	RAD51	0.699599
orf19.7149	C7_04240C_A		0.699734
orf19.261	C3_02690C_A		0.701062
orf19.7279	CR_08840C_A	NIT2	0.702062
orf19.5003	C1_13650C_A		0.704421
orf19.592	CR_08040W_A		0.704619
orf19.5559	C6_02850W_A	RAV2	0.704835
orf19.4107	C2_06110W_A		0.704988
orf19.5114	C1_08340C_A		0.705884

orf19.2272	C2_07170C_A	AFT2	0.707661
orf19.5030	C1_13920W_A	DOS2	0.707933
orf19.7544	CR_00020W_A	TLO1	0.714788
orf19.1170	C1_11500C_A	ARO7	0.718319
orf19.4269	C5_02560C_A		0.719317
orf19.1843	CR_06900C_A	ALG6	0.720251
orf19.941	C5_00480C_A	SEC14	0.722817
orf19.3319	C1_01300W_A		0.725786
orf19.5646	C4_00210W_A		0.729521
orf19.2021	C2_01010W_A	HGT8	0.730519
orf19.229	C3_02410C_A		0.731457
orf19.4665	C4_01240C_A		0.732275
orf19.905	C2_03250W_A	AVT7	0.733251
orf19.4912	C1_10420C_A		0.734331
orf19.933	C5_00560W_A		0.734822
orf19.2825	CR_02650C_A	DRE2	0.735065
orf19.7564	CR_09900C_A	DPB2	0.735506
orf19.6481	C7_02300W_A	YPS7	0.737196
orf19.1479	C2_01680C_A		0.737742
orf19.99	C6_00970C_A	HAL21	0.738386
orf19.367	C4_00040W_A	CNH1	0.738634
orf19.5217	C2_05970C_A	TES1	0.741447
orf19.2686	C4_03050C_A		0.742858
orf19.2842	CR_02850C_A	GZF3	0.745069
orf19.2093	C2_00380C_A	RFA1	0.745164
orf19.4828	C1_09650W_A		0.74538
orf19.5579	C6_03000C_A		0.747091
orf19.433	C1_05320C_A		0.74767
orf19.1655.3	C3_01910C_A		0.751114
orf19.6556	C7_01680C_A		0.751175
orf19.7372	C3_05920W_A	MRR1	0.75221
orf19.820	C2_04020C_A	SDS22	0.752292
orf19.6558	C7_01660C_A		0.752346
orf19.2410	CR_03150W_A	SYS3	0.754423
orf19.4753	C1_08950W_A	PFK26	0.754423
orf19.4355	CR_03840C_A		0.75503
orf19.1179	C6_00200C_A		0.755574
orf19.5563	C6_02870W_A	RNH1	0.755605
orf19.5031	C1_13930W_A	SSK1	0.756982
orf19.3697	C7_02670W_A		0.759027
orf19.2269	C2_07140W_A		0.759186
orf19.1035	C1_03740W_A	WAR1	0.75949
orf19.2278	C2_07220W_A		0.760388
orf19.1885	C2_07460W_A	TAF4	0.760465
orf19.7078	C7_00420C_A		0.762697
orf19.200	C2_09040W_A		0.763615
orf19.6658	C5_03490C_A		0.765351
orf19.1200	C6_00380C_A		0.76561

orf19.2365	CR_07000C_A	POL2	0.765964
orf19.3006	C1_03130C_A	GGA2	0.767724
orf19.5177	C7_02850W_A		0.768198
orf19.5159	C7_03020C_A	DUG3	0.769717
orf19.899	C2_03290W_A		0.770833
orf19.2835	CR_02770C_A		0.771054
orf19.7250	C1_14350W_A		0.773076
orf19.7165	C7_04100C_A	MED9	0.773532
orf19.3829	C4_04530C_A	PHR1	0.773848
orf19.3302	C1_01140C_A		0.773877
orf19.225	C2_08850C_A		0.774189
orf19.2396	CR_03280W_A	IFR2	0.774352
orf19.7204	C7_03780C_A		0.774883
orf19.2289	C2_07320W_A	ARP3	0.776911
orf19.5688	C5_00090C_A		0.777937
orf19.7571	CR_09970W_A	UBC4	0.778499
orf19.1291	C4_05290W_A	ABZ1	0.783556
orf19.6809	C3_06920W_A		0.784792
orf19.3047	C1_03450C_A		0.78512
orf19.3218	C5_03940C_A		0.786263
orf19.1412	C4_04350W_A		0.787043
orf19.5296	C4_04130W_A		0.788216
orf19.4024	C5_05300W_A	RIB5	0.788322
orf19.7021	C7_00930W_A	GPH1	0.788719
orf19.202	C2_09020W_A	CDC47	0.790585
orf19.4186	C4_00570C_A	PCT1	0.791497
orf19.1600	C2_09420W_A		0.791777
orf19.687	C6_01960W_A		0.792034
orf19.1582	C2_02570W_A		0.792643
orf19.3139	C4_06710W_A		0.793259
orf19.1628	C3_02170C_A	LAP41	0.793673
orf19.2286	C2_07290W_A		0.794676
orf19.2016	C2_01090C_A	ERG28	0.7954
orf19.4395	CR_03520C_A		0.797231
orf19.4545	C1_01790W_A	SWI4	0.79927
orf19.364	C4_00020W_A		0.803916
orf19.7362	C3_05810C_A	SKN1	0.804561
orf19.4030	C5_05350W_A		0.804924
orf19.6812	C3_06890W_A	PMT2	0.804994
orf19.1723	C3_01280W_A		0.807263
orf19.505	CR_04190W_A	SRV2	0.807376
orf19.1734	CR_04720C_A		0.812596
orf19.5194.1	C1_04430C_A		0.813176
orf19.7554	CR_09830W_A		0.813586
orf19.3548.1	C2_05180W_A	WH11	0.814029
orf19.1409.2	C4_04380C_A		0.814191
orf19.2426	CR_03020C_A		0.814956
orf19.84	C6_00830C_A	CAN3	0.815871

orf19.908	C2_03230C_A	FEN12	0.817895
orf19.4105	C2_06130W_A	CSM3	0.818278
orf19.2862	CR_02990C_A	RIB1	0.819513
orf19.5773	C6_03960W_A		0.821085
orf19.2875	C4_06590W_A		0.82154
orf19.55	C1_05010C_A		0.822301
orf19.7314	CR_09180W_A	CDG1	0.823996
orf19.53	C1_04990C_A		0.825451
orf19.5843	CR_05610C_A	SRR1	0.826041
orf19.7140	C7_04280C_A		0.827056
orf19.3392	C6_01900C_A	DOG1	0.827974
orf19.5281	C1_11860W_A		0.830031
orf19.7313	CR_09170C_A	SSU1	0.832145
orf19.6189	C3_07940W_A		0.832731
orf19.2465	C1_05850W_A	POL32	0.833647
orf19.873.1	C2_03470C_A	COX6	0.833693
orf19.6796	C3_07040C_A	YSA1	0.836154
orf19.2796	C1_07490C_A		0.836225
orf19.3796	C4_04830W_A	DCR1	0.83672
orf19.3711	CR_07820W_A		0.841778
orf19.5076	C1_08030W_A	PFY1	0.843461
orf19.3846	C4_04410C_A	LYS4	0.843597
orf19.5065	C1_07930C_A	ERD1	0.846748
orf19.97	C6_00960W_A	CAN1	0.848296
orf19.3648	C6_00770C_A		0.848715
orf19.5862	C3_04200W_A	AFP99	0.851341
orf19.5182	C7_02790C_A	POL3	0.851892
orf19.2672	C4_03180W_A	NCP1	0.856183
orf19.2739	C4_02600C_A		0.858029
orf19.2119	C2_00140W_A	NDT80	0.858709
orf19.669	C1_11340W_A	PRM1	0.859
orf19.4127	C2_04850C_A		0.859493
orf19.4633	C4_01510W_A		0.861316
orf19.842	C2_03790C_A	ASR3	0.862285
orf19.6875	C2_05650W_A	VPS35	0.862673
orf19.681	C1_11210C_A	HAP43	0.864367
orf19.50	C1_04960C_A		0.865391
orf19.5671	C4_00420C_A		0.86542
orf19.6059	C1_00490C_A	TTR1	0.866603
orf19.7329	CR_09340W_A		0.866951
orf19.6770	C3_07280C_A		0.868343
orf19.1354	C2_08290C_A	UCF1	0.869285
orf19.1978	C5_00890C_A	GIT2	0.869879
orf19.2459	C1_05900W_A		0.872009
orf19.2277	C2_07210C_A	TPK2	0.885884
orf19.4774	C1_09160W_A	AOX1	0.886169
orf19.1866	C2_07590W_A	VMA10	0.886737
orf19.3578	C2_05490W_A		0.887076

orf19.4952.1	C1_13260W_A		0.888228
orf19.7276.1	C1_14590C_A	TLO4	0.890267
orf19.719	CR_06470W_A		0.891371
orf19.2611	CR_02110W_A	MCM6	0.893801
orf19.3061.1	C1_03620C_A		0.893821
orf19.5651	C4_00260W_A		0.894651
orf19.1043	C1_04110W_A		0.898761
orf19.5318	C2_10440C_A	RAD1	0.901076
orf19.2826	CR_02670C_A		0.902181
orf19.4929	C1_13010W_A		0.908725
orf19.3869	CR_06140W_A		0.915051
orf19.1011	CR_05300C_A	MNN21	0.919896
orf19.1307	C4_03710C_A		0.923971
orf19.1799	C4_05430C_A	GAP5	0.925051
orf19.1655	C3_01930W_A	PXP2	0.927296
orf19.3363	C4_03360C_A	VTC4	0.929621
orf19.1906	C2_00130W_A		0.929871
orf19.2419	CR_03070W_A		0.931144
orf19.3127	C4_06820C_A	CZF1	0.933373
orf19.5642	C4_00170W_A		0.934471
orf19.7023	C7_00910C_A		0.936924
orf19.396	C1_08510W_A	EAF6	0.937927
orf19.1190	C6_00300C_A	STV1	0.938875
orf19.4734	C1_08760W_A		0.939191
orf19.6400	CR_08330W_A		0.94002
orf19.542.2	CR_04520W_A	MIM1	0.942906
orf19.4371	CR_03720W_A	TAL1	0.946524
orf19.3444	C6_01400W_A		0.948164
orf19.3910	C5_04260W_A		0.949714
orf19.4215	C6_00440C_A	FET34	0.950506
orf19.1285	C5_04050W_A		0.953527
orf19.1180	C6_00210W_A		0.955223
orf19.5178	C7_02840C_A	ERG5	0.956137
orf19.3577	C2_05470W_A	COQ5	0.95996
orf19.6211	C1_06970C_A		0.960001
orf19.6534.2	C7_01850C_A	CRN1	0.960065
orf19.909	C2_03220C_A	STP4	0.964577
orf19.7445	C3_06560W_A		0.965144
orf19.3590	C2_08810C_A	IPP1	0.966895
orf19.3826	C4_04560C_A		0.969855
orf19.4953	C1_13270W_A		0.971131
orf19.7570	CR_09960C_A	UGA3	0.971152
orf19.7326	CR_09310W_A		0.972789
orf19.3449	C6_01350W_A		0.977343
orf19.1901	C2_07350W_A	MCM3	0.982146
orf19.6447	CR_08700C_A	ARF1	0.986663
orf19.6684	C7_03520W_A	PNC1	0.988873
orf19.3380	C4_03510C_A	HWP2	0.990351

orf19.577	C5_00750C_A		0.993522
orf19.6905	C7_01230C_A		0.995563
orf19.2710	C4_02860W_A		0.996294
orf19.7306	CR_09100C_A		0.99669
orf19.3916	C5_04310W_A		1.00362
orf19.4056	C1_05140W_A	BRG1	1.00552
orf19.968	C5_00270W_A	PGA14	1.00564
orf19.1097	C6_04380W_A	ALS2	1.00709
orf19.4943	C1_13160W_A	PSA2	1.00776
orf19.1357	C2_09950W_A	FCY21	1.00807
orf19.909.1	C2_03210W_A		1.01
orf19.3487	CR_07690W_A		1.01391
orf19.392	C1_08470W_A		1.0152
orf19.4555	C6_04130C_A	ALS4	1.01535
orf19.5617	C6_03290W_A		1.02029
orf19.4043	C5_05440C_A		1.02168
orf19.3004	C1_03120W_A		1.02408
orf19.6487	C7_02260W_A		1.02549
orf19.1861	C2_07640W_A		1.03051
orf19.4185	C4_00580W_A		1.03151
orf19.508	CR_04210C_A	QDR1	1.03375
orf19.6224	C1_06860W_A		1.03397
orf19.2965.1	C1_02770W_A		1.03407
orf19.3447	C6_01370W_A		1.0353
orf19.769	C1_04750W_A	IFE1	1.03573
orf19.1193	C6_00330C_A	GNP1	1.038
orf19.5213.2	C2_05930W_A	COX9	1.03828
orf19.2467	C1_05840W_A	PRN1	1.03868
orf19.7419	C3_06340W_A	HNT2	1.03924
orf19.796	C2_04240C_A	HYM1	1.03969
orf19.4786	C1_09270W_A	FGR43	1.04018
orf19.3469	C6_02280W_A		1.04092
orf19.7357	C3_05760W_A		1.04598
orf19.251	C3_02610C_A	GLX3	1.04999
orf19.2681	C4_03100W_A	RBT7	1.05165
orf19.3521	C2_04880C_A	ARH2	1.05236
orf19.775	C1_04700C_A		1.0548
orf19.1860.1	C2_07650C_A		1.05742
orf19.3281	CR_00750C_A		1.05793
orf19.4151	C5_01570C_A	SPO1	1.06367
orf19.7577	CR_10010C_A	MSS51	1.06446
orf19.2853	CR_02960W_A		1.06752
orf19.4528	C1_01950C_A		1.07235
orf19.5114.1	C1_08350C_A		1.07286
orf19.5450	C3_00200C_A	ETR1	1.07561
orf19.6318	CR_04730W_A		1.07622
orf19.6261	C1_06520C_A	BPH1	1.07904
orf19.6191	C3_07970C_A	TLO8	1.07984

orf19.7499	CR_00350W_A		1.0811
orf19.6055	C1_00530C_A		1.08252
orf19.3701	C7_02630W_A		1.08267
orf19.5612	C6_03250W_A	BMT4	1.08675
orf19.5158	C7_03030W_A		1.08827
orf19.5063	C1_07900W_A	COI1	1.08853
orf19.6440	CR_08650C_A		1.09009
orf19.5282	C1_11850W_A		1.09156
orf19.4354	CR_03830C_A	MCM2	1.09283
orf19.2593	CR_01930C_A	BIO2	1.09414
orf19.3971	C5_04850W_A		1.09424
orf19.6638	CR_05740C_A	PTC4	1.09443
orf19.2917.1	C4_06200W_A		1.09644
orf19.2613	CR_02130W_A	ECM4	1.09801
orf19.1201	C6_00390W_A		1.09822
orf19.1891	C2_07400C_A		1.10064
orf19.3310	C1_01220C_A		1.10441
orf19.23	C2_06460W_A	RTA3	1.10447
orf19.6337	C6_00030W_A	TLO13	1.10472
orf19.791	C2_04290W_A	RIM11	1.10509
orf19.6800	C3_07010W_A	POS5	1.10645
orf19.2618	CR_02170W_A	MET2	1.10738
orf19.2839	CR_02810W_A	CIRT4B	1.10798
orf19.2395	CR_03290C_A	IME2	1.1124
orf19.3639	C2_08420W_A		1.11248
orf19.4720	C1_08620W_A	CTR2	1.11364
orf19.6837	C1_04450C_A	FMA1	1.1168
orf19.4620	C4_01740W_A	TIM12	1.1196
orf19.4616	C4_01770W_A	POL30	1.12041
orf19.6580	C7_01490W_A		1.12129
snR10c	C5_02850W_A		1.12292
orf19.2173	C2_08120W_A	MAF1	1.12468
orf19.1365	C2_09860C_A		1.1248
orf19.4368	CR_03690W_A		1.12893
orf19.1863	C2_07620W_A		1.13096
orf19.4609	C4_01840C_A		1.13126
orf19.4304	C5_02790C_A	GAP1	1.1317
orf19.2640	C5_03390C_A	FUR1	1.13349
orf19.7581	CR_10060W_A		1.13401
orf19.7163	C7_04120W_A		1.13663
orf19.166	CR_02560C_A	ASG1	1.13813
orf19.6082	C1_00210C_A		1.14002
orf19.4742	C1_08840W_A		1.14034
orf19.6323	C6_00140C_A	HPA2	1.14075
orf19.1162	C1_11580W_A		1.14224
orf19.5965	C3_04970C_A		1.14374
orf19.5210	C2_05860C_A		1.14818
orf19.6951	C3_03680W_A		1.14849

orf19.3753	CR_02190C_A	SEF1	1.15061
orf19.3781	C4_04990C_A		1.15234
orf19.6744	C3_07450C_A		1.15487
orf19.2531	CR_01470W_A	CSP37	1.15634
orf19.795	C2_04250W_A	VPS36	1.16335
orf19.2114	C2_00180C_A		1.1645
orf19.2834	CR_02760C_A	RPD3	1.16606
orf19.1880	C2_07490W_A	HEM15	1.16644
orf19.6398	CR_08310C_A		1.1679
orf19.5054	C1_07840W_A		1.17548
orf19.2738	C4_02610C_A	SUL2	1.17625
orf19.4109	C2_06100W_A	PMT4	1.17756
orf19.7344	CR_09490W_A		1.17859
orf19.1474	C2_01640W_A	SLA1	1.18113
orf19.704	CR_06700C_A	SOL3	1.18141
orf19.6396	CR_08300C_A		1.18295
orf19.7360	C3_05790C_A		1.18804
orf19.6117	CR_07480W_A		1.19024
orf19.3881	CR_06290C_A		1.19099
orf19.1113	C5_03810C_A		1.19287
orf19.7503	CR_00300W_A	CDA2	1.19303
orf19.6529	C7_01920W_A	CDC34	1.19316
orf19.7297	CR_09010C_A		1.19463
orf19.6742	C3_07460W_A		1.19675
orf19.4150	C5_01560C_A		1.20225
orf19.3802	C4_04780W_A	PMT6	1.20234
orf19.1564	C2_02410W_A		1.20598
orf19.431	C1_05340C_A	ZCF2	1.20874
orf19.5992	C3_05170W_A	WOR2	1.21121
orf19.2097	C2_00350W_A		1.21314
orf19.1546	C2_02280W_A		1.21349
orf19.7611	CR_10350C_A	TRX1	1.21458
orf19.4631	C4_01530C_A	ERG251	1.21491
orf19.2175	C2_08100W_A		1.2165
orf19.1338	C7_03370C_A		1.21756
orf19.7550	CR_09780C_A	IFA14	1.22004
orf19.4524	C1_02010C_A	ZCF24	1.23148
orf19.5779	C2_03160C_A	RNR1	1.23297
orf19.5257	C1_12080W_A	LCB4	1.23366
orf19.2472.1	C1_05790W_A		1.23576
orf19.1592	C2_04300C_A		1.23719
orf19.4290	C5_02710W_A	TRR1	1.23877
orf19.5663	C4_00360C_A	RCH1	1.23966
orf19.3522	C2_04910W_A		1.24163
orf19.3922	C5_04360C_A		1.24207
orf19.6952	C3_03670W_A		1.24835
orf19.4824	C1_09610W_A		1.24919
orf19.5527	C6_02580W_A		1.2524

orf19.2047	C2_00770W_A		1.25509
orf19.2006.1	C2_01180W_A	COX17	1.25578
orf19.105	C6_01030W_A	HAL22	1.25983
orf19.6813	C3_06880W_A		1.26689
orf19.915	CR_06570C_A		1.26921
orf19.1979	C5_00880C_A	GIT3	1.27129
orf19.5320	C2_10450W_A	NCE4	1.27315
orf19.7612	CR_10360C_A	CTM1	1.27698
orf19.953.1	C5_00370W_A	COF1	1.28178
orf19.5447	C3_00220W_A	HGT19	1.28428
orf19.1653	C3_01950C_A		1.28884
orf19.7405	C3_06270C_A		1.29023
orf19.137	C6_01300W_A		1.29041
orf19.21	C2_06430C_A		1.29255
orf19.691	C6_02010C_A	GPD2	1.29281
orf19.7551	CR_09790W_A	ALO1	1.2946
orf19.2670	C4_03200C_A		1.29583
orf19.6209	C1_06980C_A		1.29679
orf19.5025	C1_13870W_A	MET3	1.29795
orf19.5394.1	C3_00620C_A		1.29881
orf19.4044	C5_05450C_A	MUM2	1.29988
orf19.7086	C7_00340C_A		1.30317
orf19.6344	C1_12780W_A	RBK1	1.30652
orf19.2098	C2_00340C_A	ARO8	1.30757
orf19.6881	C2_05690C_A	YTH1	1.31208
orf19.5227	C1_12370W_A		1.31604
orf19.7522	CR_00130C_A		1.31675
orf19.3761	C1_12550C_A	CDC54	1.32207
orf19.4982	C1_13510C_A	TGL99	1.32614
orf19.4595	C4_01970W_A		1.32759
orf19.2429	C1_06160W_A		1.32878
orf19.3940.1	C5_04560C_A	CUP1	1.32973
orf19.6225	C1_06850W_A	PCL7	1.33431
orf19.2551	CR_01620C_A	MET6	1.34655
orf19.2334	C1_10830W_A	BIG1	1.35068
orf19.2496	C3_00930W_A	ATO2	1.35176
orf19.7531	CR_00090C_A		1.35177
orf19.1149	C1_11700C_A	MRF1	1.35792
orf19.2768	C4_02360W_A	AMS1	1.35988
orf19.6472	C7_02380C_A	CYP1	1.36308
orf19.1857	CR_06770C_A		1.36793
orf19.410.3	C1_05560W_A	RIB4	1.36817
orf19.1158	C1_11620W_A		1.36925
orf19.3646	C6_00790C_A	CTR1	1.37561
orf19.4898	C1_10280C_A		1.37582
orf19.2937	C1_02480W_A	PMM1	1.37837
orf19.4317	C5_02930C_A	GRE3	1.37967
orf19.7199	C7_03830C_A		1.38068

orf19.7434	C3_06450W_A	GLG2	1.38709
orf19.6817	C3_06850W_A	FCR1	1.3874
orf19.4621	C4_01730C_A		1.39343
orf19.1963	C5_01080C_A	GDS1	1.39362
orf19.3746	CR_02240C_A	OPT2	1.39492
orf19.5526	C6_02570C_A	SEC20	1.39806
orf19.1827	C1_06220C_A		1.3986
orf19.2608	CR_02070C_A	ADH5	1.39986
orf19.768	C1_04760C_A	SYG1	1.40855
orf19.5312	C4_04000W_A	MET4	1.4121
orf19.6077	C1_00310W_A		1.41874
orf19.6983	C3_05450C_A		1.42816
orf19.1152	C1_11670W_A		1.42959
orf19.409	C1_08610C_A		1.42981
orf19.6402	CR_08340W_A	CYS3	1.43148
orf19.4862.2	C1_09960W_A	PET100	1.43277
orf19.733	CR_07160C_A		1.43473
orf19.646	CR_05050W_A	GLN1	1.43877
orf19.3966	C5_04800W_A	CRH12	1.44165
orf19.6058	C1_00500C_A	GLO1	1.44796
orf19.5023	C1_13850C_A	DAL7	1.44867
orf19.6661	C5_03520W_A		1.45641
orf19.5630	C6_03410C_A	APA2	1.45681
orf19.6757	C3_07340W_A	GCY1	1.45878
orf19.4550	C1_01750W_A		1.46145
orf19.4013	C5_05200C_A		1.46197
orf19.1887	C2_07440C_A		1.46209
orf19.1974	C5_00930C_A	TFS1	1.46647
orf19.33	C2_06570C_A		1.46763
orf19.1946	C5_01230C_A		1.47205
orf19.7459	C3_06700C_A		1.47235
orf19.4752	C1_08940C_A	MSN4	1.47322
orf19.2989	C1_02980W_A	GOR1	1.47486
orf19.1999	C2_01250W_A		1.47655
orf19.6977	C3_05390C_A	GPI1	1.47833
orf19.7212	C1_14040W_A		1.4787
orf19.3355	C1_01650W_A	ISN1	1.48038
orf19.670	C1_11330C_A	SMT3	1.48399
orf19.3999	C5_05070W_A		1.48471
orf19.5777	C2_03170W_A		1.48561
orf19.3104	C4_07050W_A	YDC1	1.48563
orf19.1591	C2_04310W_A	ERG10	1.48785
orf19.5524	C6_02550W_A		1.49387
orf19.1599	C2_09410W_A		1.49545
orf19.3391	C6_01910W_A	ADK1	1.49781
orf19.1477	C2_01660C_A		1.49848
orf19.6594	CR_09690C_A	PLB3	1.50858
orf19.5417	C3_00480C_A	DOT5	1.51221

orf19.4967	C1_13410W_A	COX19	1.51486
orf19.1476	C2_01650W_A		1.51799
orf19.5700	C5_00010W_A	TLO11	1.51803
orf19.1434	C2_08380C_A		1.51935
orf19.6232	C1_06780W_A	NPR1	1.52173
orf19.2262	C2_07070W_A		1.52419
orf19.6739	C3_07490W_A		1.52464
orf19.5917	C3_04580C_A	STP1	1.52589
orf19.5622	C6_03340C_A	GLC3	1.52904
orf19.2529	CR_01430W_A		1.53046
orf19.3106	C4_07030W_A	MET16	1.53524
orf19.54	C1_05000W_A	RHD1	1.53609
orf19.2107	C2_00260C_A	MUQ1	1.55459
orf19.321	C3_03230C_A		1.55517
orf19.1240	C4_05590W_A		1.5583
orf19.2583.2	CR_01850C_A		1.55948
orf19.5980	C3_05090C_A		1.56609
orf19.4358	CR_03870W_A		1.5669
SNRNAU6	C3_05500W_A	SNR6	1.56727
orf19.6978	C3_05400C_A		1.56756
orf19.6659	C5_03500W_A	GAP6	1.57379
orf19.2484	C1_05670W_A		1.57544
orf19.4736	C1_08780W_A		1.57597
orf19.1961	C5_01090C_A		1.57809
orf19.4309	C5_02860C_A	GRP2	1.57906
orf19.163	CR_02580W_A	PAN6	1.59228
orf19.5242	C1_12220W_A	CDC6	1.59297
orf19.5525	C6_02560W_A		1.59547
orf19.1795.1	C4_05380C_A		1.59577
orf19.5059	C1_07880C_A	GCS1	1.59976
orf19.3885	CR_06310W_A		1.60534
orf19.6923.1	C3_03920W_A		1.606
orf19.5841	C2_02660W_A		1.61658
RDN58	CR_08790W_A	RDN58	1.61904
orf19.5326	C2_10540W_A		1.61975
orf19.6190	C3_07950C_A	SRB1	1.62173
orf19.1107	C5_03870C_A		1.62629
orf19.4816	C1_09520C_A		1.63051
orf19.1743	C2_10350C_A	ACS1	1.64347
orf19.4438	C1_07330W_A	RME1	1.66051
orf19.2761	C4_02420C_A		1.66485
orf19.4280	C5_02640W_A		1.66626
orf19.5479	C2_06180C_A	FGR12	1.66633
orf19.3780	C4_05000W_A		1.66782
orf19.4118	C2_06020W_A	CNT	1.67094
orf19.4841	C1_09760C_A	SHY1	1.67437
orf19.2655	C5_03240W_A	BUB3	1.68924
orf19.4733	C1_08750W_A	YMC2	1.7027

orf19.4523	C1_02020W_A		1.70527
orf19.5920	C3_04620C_A		1.70538
orf19.5686	C5_00100C_A		1.71135
orf19.430	C1_05350W_A	YPT53	1.71434
orf19.3263	CR_00930W_A	ATO10	1.72402
orf19.767	C1_04770C_A	ERG3	1.7255
orf19.2966	C1_02780W_A		1.73498
orf19.6222.1	C1_06870C_A		1.73622
orf19.5280	C1_11870W_A	MUP1	1.73841
orf19.5503	C7_03740C_A		1.74513
orf19.7225	C1_14180W_A		1.7469
orf19.1668	C3_01770C_A		1.75244
orf19.3337	C1_01510W_A		1.75723
orf19.223	C2_08860W_A		1.75771
orf19.776	C1_04690C_A		1.7587
orf19.5334	C2_10560C_A	ZSF1	1.76131
orf19.5197	C1_04400C_A	APE2	1.76844
orf19.4310	C5_02870C_A		1.77722
orf19.7166	C7_04090C_A		1.77983
orf19.2866	C4_06660W_A		1.79442
orf19.6570	C7_01560C_A	NUP	1.79498
orf19.1336.2	C7_03380W_A		1.79558
orf19.7196	C7_03860W_A		1.80289
orf19.7323	CR_09270C_A	CBP1	1.81501
orf19.4216	C5_02110W_A		1.84271
orf19.922	C5_00660C_A	ERG11	1.84364
orf19.6660	C5_03510C_A		1.85297
orf19.6214	C1_06940C_A	ATC1	1.87721
orf19.3131	C4_06780C_A	OYE32	1.88185
orf19.2969	C1_02810W_A	RAD16	1.88255
orf19.553	CR_04620C_A		1.8829
orf19.2132	C6_04480C_A		1.8842
orf19.6349	C1_12730W_A	RVS162	1.8913
orf19.4407	C4_05970W_A		1.89154
orf19.6138	CR_07300W_A		1.90399
orf19.6263	C1_06510C_A		1.90565
orf19.2337	C1_10800C_A	ALP1	1.90882
orf19.2107.1	C2_00250W_A	STF2	1.91698
orf19.1416	C4_04310W_A	COX11	1.92136
orf19.6998	C3_05630W_A	GTT1	1.92195
orf19.1395	C2_09590C_A		1.92568
orf19.5801	C2_03010C_A	RNR21	1.93122
orf19.7479	CR_00560W_A	NTH1	1.93891
orf19.3707	CR_07790C_A	YHB1	1.94102
orf19.238	C3_02480C_A	CCP1	1.94978
orf19.2691	C4_03000C_A		1.95153
orf19.2779	C1_07650W_A		1.96573
orf19.7396	C3_06140W_A		1.96871

orf19.5770	C6_03930W_A	OPT8	1.9688
orf19.5902	C3_04480C_A	RAS2	1.96978
orf19.7580	CR_10040W_A		1.97361
orf19.5854	C3_04090W_A	SBP1	1.97438
orf19.4624	C4_01690C_A	HRT2	1.98658
orf19.7437	C3_06490W_A		1.99349
orf19.1867	C2_07580W_A		1.99406
orf19.633	CR_04960C_A	CRG1	1.9947
orf19.5136	C7_03200C_A		1.99712
orf19.1116	C5_03780C_A		2.00155
orf19.6391	CR_08270W_A		2.00667
orf19.1796	C4_05390W_A		2.00856
snR42a	C1_07450W_A		2.00866
orf19.4436	C1_07350C_A	GPX3	2.01343
orf19.4914.1	C1_12850W_A	BLP1	2.01633
orf19.7325	CR_09300C_A	SCO1	2.01756
orf19.951	C5_00390C_A		2.02742
orf19.2069	C2_00580C_A	SMF3	2.03403
orf19.6225.1	C1_06840C_A		2.0368
orf19.1654	C3_01940C_A		2.04236
orf19.904	C2_03260W_A		2.04502
orf19.1290	C4_05300W_A	XKS1	2.05541
orf19.4784	C1_09250W_A	CRP1	2.05637
orf19.2048	C2_00760C_A		2.05697
orf19.3038	C1_03380W_A	TPS2	2.05763
orf19.3325	C1_01360C_A		2.07219
orf19.6200	C1_07040C_A		2.07426
orf19.57	C1_05030C_A	PSF2	2.07461
orf19.5578	C6_02990W_A		2.07863
orf19.4477	C1_04020C_A	CSH1	2.08367
orf19.6065	C1_00420W_A		2.09724
orf19.3160	C5_02080C_A	HSP12	2.09775
orf19.5399	C3_00600W_A	IFF11	2.09818
orf19.4738	C1_08800W_A		2.10461
orf19.3364	C4_03370C_A		2.114
orf19.844	C2_03770C_A	STE11	2.11511
orf19.3656	C6_00720C_A	COX15	2.13504
orf19.3515	CR_05440W_A		2.13793
orf19.6257	C1_06550W_A	GLT1	2.14092
orf19.1159	C1_11610C_A		2.15773
orf19.2324	C1_10930C_A	UBA4	2.1583
orf19.2172	C2_08130W_A	ARA1	2.16867
orf19.2525	CR_01400W_A	LYS12	2.16958
orf19.3117	C4_06920C_A	CSA2	2.17804
orf19.5635	C4_00120W_A	PGA7	2.17907
orf19.7404	C3_06280W_A		2.1845
orf19.5408	C3_00550C_A	HRK1	2.18696
orf19.4099	C2_06170C_A	ECM17	2.21531

orf19.1417	C4_04300C_A		2.21757
orf19.4833	C1_09690W_A	MLS1	2.22008
orf19.6993	C3_05580C_A	GAP2	2.23352
orf19.7395	C3_06130W_A		2.24521
orf19.2601	CR_02000C_A	HEM1	2.24529
orf19.4747	C1_08880W_A	HEM14	2.25951
orf19.1802	C4_05460C_A	OFD1	2.26202
orf19.946	C5_00430W_A	MET14	2.26273
orf19.3915	C5_04300C_A		2.26441
orf19.1287	C5_04030W_A		2.271
orf19.6078	C1_00290W_A	POL93	2.27175
orf19.1114	C5_03800W_A		2.27201
orf19.744	C4_05140C_A	GDB1	2.27359
orf19.2602	CR_02020C_A	OPT1	2.2857
orf19.2474	C1_05770C_A	PRC3	2.29359
orf19.3749	CR_02220C_A	OPT3	2.29634
ITS2	CR_08800W_A	ITS2	2.31614
orf19.260	C3_02680C_A	SLD1	2.31793
orf19.5729	C6_03610W_A	FGR17	2.33025
orf19.5592	C6_03090W_A		2.3307
orf19.411	C1_05540C_A		2.33918
orf19.7251	C1_14360C_A	WSC4	2.34016
orf19.4737	C1_08790W_A	TPO3	2.34632
orf19.1060	C1_04250C_A		2.35121
orf19.391	C1_08460C_A	UPC2	2.36063
orf19.4928	C1_12970C_A	SEC2	2.37269
orf19.6937	C3_03800W_A	PTR22	2.39089
orf19.1381	C2_09710C_A		2.40566
orf19.4287	C5_02690W_A		2.41073
orf19.6608	CR_09530C_A		2.41312
orf19.6079	C1_00270W_A		2.41788
orf19.1314	C4_03600C_A		2.42006
orf19.5741	C6_03700W_A	ALS1	2.4523
orf19.5323	C2_10480W_A	MDH1-3	2.46003
orf19.1616	C3_02300W_A	FGR23	2.4613
orf19.3278	CR_00780C_A	GSY1	2.4633
orf19.322	C3_03240C_A		2.4682
orf19.6640	CR_05720W_A	TPS1	2.46948
orf19.6450	CR_08740W_A		2.4944
orf19.2841	CR_02820W_A	PGM2	2.50406
orf19.4664	C4_01250W_A	NAT4	2.50565
orf19.434	C1_05300C_A	PRD1	2.51044
orf19.2261	C2_07060W_A		2.51225
orf19.6816	C3_06860C_A		2.51474
orf19.3727	CR_02400W_A	PHO112	2.5179
SNRNAU1	CR_03190C_A		2.52188
orf19.2619	CR_02180W_A	PHO113	2.52483
orf19.5911	C3_04550C_A	CMK1	2.5268

orf19.3378	C4_03500C_A		2.54746
orf19.1889	C2_07420W_A		2.55164
orf19.5005	C1_13670W_A	OSM2	2.55645
orf19.4444	C1_07230W_A	PHO15	2.57353
orf19.4170	C4_00720W_A	CSP2	2.57555
orf19.1027	C1_03820W_A	PDR16	2.58148
orf19.5645	C4_00200C_A	MET15	2.58375
orf19.2941	C1_02520W_A	SCW4	2.61488
orf19.7596	CR_10200W_A		2.61806
orf19.4076	C2_09140C_A	MET10	2.65077
orf19.6559	C7_01650W_A		2.66414
orf19.388	C1_08430W_A	CAF16	2.6721
orf19.6747	C3_07430W_A		2.6741
orf19.1614	C3_02310W_A	MEP1	2.68707
orf19.1169	C1_11510C_A		2.70055
orf19.2584	CR_01860W_A	OPT9	2.70148
orf19.4048	C5_05480W_A	DES1	2.70933
orf19.2335	C1_10820C_A		2.71364
orf19.4842	C1_09770W_A		2.74499
orf19.550	CR_04590C_A	PDX3	2.78014
orf19.1275	C4_05880W_A	GAT1	2.78482
orf19.489	CR_04060C_A	DAP1	2.79443
orf19.6540	C7_01800C_A	PFK2	2.81584
ITS1	CR_08780W_A	ITS1	2.81912
orf19.5078	C1_08060W_A	OFR1	2.81943
orf19.333	C3_03360W_A	FCY2	2.83162
orf19.1563	C2_02400W_A	ECM3	2.83948
orf19.5713	C6_03480W_A	YMX6	2.84143
orf19.2251	C2_06970W_A	AAH1	2.85287
orf19.542	CR_04510W_A	HXK2	2.89807
orf19.2693	C4_02990C_A	GST2	2.90301
orf19.4581	C4_02100C_A	GPI14	2.91025
orf19.125	C6_01180C_A	EBP1	2.91442
orf19.4580	C4_02110W_A		2.9231
orf19.1067	C1_04320W_A	GPM2	2.92973
orf19.4617	C4_01760W_A		2.94367
orf19.406	C1_08590C_A	ERG1	2.99784
orf19.5113	C1_08330C_A	ADH2	3.00259
orf19.5844	CR_05580C_A		3.02118
orf19.7356	C3_05750C_A		3.02805
orf19.5180	C7_02810W_A	PRX1	3.02903
orf19.5812	C2_02930C_A		3.04856
orf19.347	C3_03490W_A	RSN1	3.04962
orf19.3888	CR_06340C_A	PGI1	3.05295
orf19.5348	C2_10690W_A	TPS3	3.08695
orf19.5079	C1_08070W_A	CDR4	3.09939
orf19.4530.1	C1_01930W_A		3.12208
orf19.3433	C6_01510W_A	OYE23	3.22588

orf19.3820	C4_04620C_A		3.22619
orf19.4836	C1_09720W_A	URA1	3.23238
orf19.2372	C7_02600C_A		3.23934
orf19.5000	C1_13630W_A	CYB2	3.24084
orf19.5228	C1_12360C_A	RIB3	3.25388
orf19.1756	C2_10240W_A	GPD1	3.26412
orf19.3803	C4_04770C_A	MNN22	3.26442
orf19.780	C1_04660W_A	DUR1,2	3.28056
orf19.5820	C2_02850W_A	UGA6	3.34568
orf19.3822	C4_04590W_A	SCS7	3.3526
orf19.2248	C2_06940C_A	ARE2	3.37227
orf19.3442	C6_01420C_A		3.37938
orf19.164	CR_02570C_A		3.38545
orf19.1667.1	C3_01780C_A		3.38737
orf19.7278	C1_14630C_A		3.42468
orf19.6882	C2_05700W_A	OSM1	3.43175
orf19.111	C6_01060C_A	CAN2	3.46165
orf19.5103	C1_08240C_A		3.47884
orf19.6116	CR_07490C_A	GLK4	3.51053
orf19.1048	C1_04140W_A	IFD6	3.54866
orf19.2179	C2_08050C_A	SIT1	3.55164
orf19.539	CR_04480C_A	LAP3	3.5524
orf19.2803	C3_04060C_A	HEM13	3.64911
orf19.787.1	C4_03960W_A		3.6546
orf19.5818	C2_02860W_A	SUR2	3.66211
orf19.903	C2_03270W_A	GPM1	3.69222
orf19.5785	C2_03110W_A		3.70117
orf19.6745	C3_07440W_A	TPI1	3.7053
orf19.1862	C2_07630C_A		3.75467
orf19.1034	C1_03750W_A		3.83263
orf19.1691	C3_01540W_A		3.83525
orf19.3651	C6_00750C_A	PGK1	3.86007
orf19.3053	C1_03510C_A		3.86304
orf19.7397.3	C3_06170C_A		3.87162
orf19.4777	C1_09190C_A	DAK2	3.89688
orf19.7227	C1_14190C_A		3.93284
orf19.1433	C2_08390W_A		4.01823
orf19.5842	C2_02650C_A		4.0494
orf19.3967	C5_04810W_A	PFK1	4.07611
orf19.4716	C4_06120W_A	GDH3	4.11977
orf19.5742	C6_03710W_A	ALS9	4.17835
orf19.5811	C2_02940W_A	MET1	4.29477
orf19.4773	C1_09150W_A	AOX2	4.34851
orf19.2762	C4_02410C_A	AHP1	4.42355
orf19.5437	C3_00320W_A	RHR2	4.6099
orf19.2371	C7_02610C_A		4.64274
orf19.5288	CR_05340C_A	IFE2	4.82394
orf19.4612	C4_01800W_A		4.83234

orf19.7676	CR_10840C_A	XYL2		4.89239
orf19.734	CR_07150W_A	GLK1		5.08067
orf19.5784	C2_03120W_A	AMO1		5.33438
orf19.689	C6_01990W_A	PLB1		6.1803
orf19.5674	C4_00450C_A	PGA10		6.33201
orf19.1868	C2_07570W_A	RNR22		6.53408
orf19.3120	C4_06910W_A			6.61001
orf19.7417	C3_06180C_A	TSA1		8.42515
orf19.3868	CR_06130C_A		inf	
